# Improved Acid Resistance of a Metal–Organic Cage Enables Cargo Release and Exchange between Hosts

**DOI:** 10.1002/anie.202001059

**Published:** 2020-03-18

**Authors:** Lin Xu, Dawei Zhang, Tanya K. Ronson, Jonathan R. Nitschke

**Affiliations:** ^1^ Department of Chemistry University of Cambridge Lensfield Road Cambridge CB2 1EW UK; ^2^ Shanghai Key Laboratory of Green Chemistry and Chemical Processes School of Chemistry and Molecular Engineering East China Normal University 3663 N. Zhongshan Road Shanghai 200062 P. R. China

**Keywords:** acid resistance, cargo delivery, cargo exchange, metal–organic cages, supramolecular chemistry

## Abstract

The use of di(2‐pyridyl)ketone in subcomponent self‐assembly is introduced. When combined with a flexible triamine and zinc bis(trifluoromethanesulfonyl)imide, this ketone formed a new Zn_4_L_4_ tetrahedron **1** bearing twelve uncoordinated pyridyl units around its metal‐ion vertices. The acid stability of **1** was found to be greater than that of the analogous tetrahedron **2** built from 2‐formylpyridine. Intriguingly, the peripheral presence of additional pyridine rings in **1** resulted in distinct guest binding behavior from that of **2**, affecting guest scope as well as binding affinities. The different stabilities and guest affinities of capsules **1** and **2** enabled the design of systems whereby different cargoes could be moved between cages using acid and base as chemical stimuli.

## Graphical abstract

**A strategy for improving** the acid resistance of a tetrahedral cage has been developed by incorporating additional free pyridyl units on its vertices. The guest binding properties of the cage are also altered compared to the analogous tetrahedron without these peripheral groups, allowing the functions of complete cargo delivery and exchange between the two capsules by using acid and base as chemical stimuli.

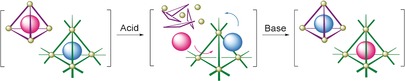

Metal–organic cages^1^ have wide‐ranging applications, including molecular sensing,^2^ catalysis,^3^ guest sequestration,^4^ and stabilization of reactive species.^5^ Compared to non‐dynamic covalent cages,^6^ however, the coordination bonds of metal–organic capsules render them sensitive to opening in the presence of acids or bases,^7^ potentially limiting their practical applications. Strategies to render these capsules more robust to environmental changes could thus lead to broader usefulness.

The reversible nature of their coordination bonds7a, 8 also provides metal–organic cages with potentially useful stimuli‐responsive properties.^9^ Such stimuli‐responsive hosts,^10^ capable of trapping and releasing guests in a controlled manner, are finding new uses.^11^ Systems consisting of multiple hosts and guests together allow for complex functions to be designed,^12^ such as functional mimicry of multi‐enzyme systems.^13^ Within such a system, cargo delivery from the cavity of one molecular container to another would imitate the sequential transformations in the synthesis of natural products,^14^ whereby the intermediate product from one enzymatic transformation becomes the substrate of another enzyme.

We hypothesized that di(2‐pyridyl)ketone might be employed in place of 2‐formylpyridine during the construction of cages by subcomponent self‐assembly.^12^ Such new cages might exhibit enhanced acid stability due to the free basic pyridyl units at their corners. This concept was realized through the synthesis of tetrahedron **1** (Figure 1 a), which was shown to be capable of cargo release and exchange between the cavities of **1** and its analogue **2**, incorporating 2‐formylpyridine residues, using acid and base as chemical stimuli.

**Figure 1:**
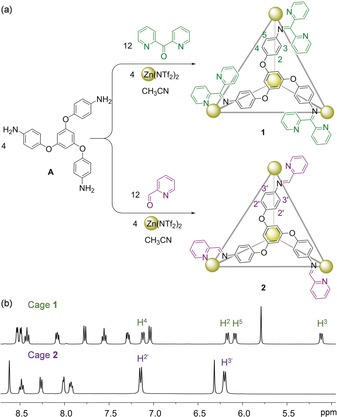
a) Subcomponent self‐assembly of tetrahedra **1** and **2** and b) their ^1^H NMR spectra (CD_3_CN, 400 MHz, 298 K). The peaks of the phenylene rings in each structure are labelled. Full structural assignments are provided in the Supporting Information, Sections 2.1 and 2.2.

The reactions of tritopic amine subcomponent **A** 15 (4 equiv) with zinc(II) bis(trifluoromethanesulfonyl)imide (triflimide, Tf_2_N^−^, 4 equiv) and di(2‐pyridyl)ketone (12 equiv) or 2‐formylpyridine (12 equiv) in acetonitrile afforded tetrahedra **1** and **2** (Figure 1; Supporting Information, Figures S1–S20). The ^1^H NMR spectra of **1** and **2** displayed only one set of ligand signals, consistent with the formation of *T*‐symmetric tetrahedra.15b Interestingly, the phenylene protons of cage **2** showed two signals (H^2′^ and H^3′^ in Figure 1) at 298 K, whereas those of **1** exhibited four distinct signals (H^2^–H^5^ in Figure 1). We infer this different behavior to be due to the steric hindrance caused by the additional free pyridine rings at the corners of **1**, which restrict the rotation of the neighboring phenylene rings and render the cavity of **1** more rigid than that of **2**.

Slow vapor diffusion of diethyl ether into acetonitrile solutions of **1** in the presence of other anions, including BF_4_^−^, TfO^−^, ReO_4_^−^, PF_6_^−^, and SbF_6_^−^, allowed us to obtain crystals of the anion adducts X^−^⊂**1** suitable for X‐ray diffraction studies (Figure 2 a–e).^16^ In each case four ligands bridge four octahedral zinc(II) centers. Each ligand caps a face of the tetrahedron and displays a *C*_3_‐symmetric propeller‐like configuration.^17^ Only one pyridyl unit per di(2‐pyridyl)ketone moiety coordinates to the zinc(II) center, resulting in twelve uncoordinated pyridyl units around the vertices of **1** in each case. These free pyridine rings orient so as to minimize steric clash with their neighboring phenylene rings (Supporting Information, Figure S21). In each case an anion is observed inside the cavity of **1**, with the cavity volume expanding from 134 Å^3^ in [BF_4_^−^⊂**1**] to 148 Å^3^ in [TfO^−^⊂**1**] (Supporting Information, Table S1).

**Figure 2:**
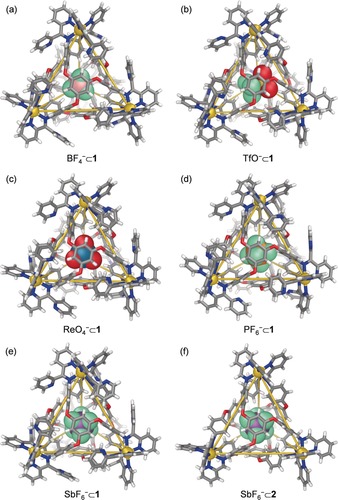
Crystal structures of a) BF_4_^−^⊂**1**, b) TfO^−^⊂**1**, c) ReO_4_^−^⊂**1**, d) PF_6_^−^⊂**1**, e) SbF_6_^−^⊂**1**, and f) SbF_6_^−^⊂**2**. Disorder, unbound counterions, and solvents of crystallization are omitted for clarity.

The crystal structure of [SbF_6_^−^⊂**2**] (Figure 2 f) was obtained in the same way, showing an analogous tetrahedral framework without the twelve peripheral pyridyl units. The cavity volumes of cages **1** and **2** with SbF_6_^−^ bound inside are similar, at 143 Å^3^ and 146 Å^3^, respectively (Supporting Information, Table S1).

The peripheral differences in the structures of **1** and **2** resulted in different guest binding behavior in solution (Supporting Information, Figures S23–S59). The smallest anion investigated, BF_4_^−^, bound within **1** (*K*_a_=2.2×10^3^ m ^−1^), whereas no interaction was observed with cage **2**. Cage **1** also bound all other anions (*K*_a_=10^6^–10^7^ m ^−1^) more strongly than **2** (*K*_a_=10^4^–10^5^ m ^−1^) (Supporting Information, Table S2). In contrast, cage **2** encapsulated neutral guests (Figure 3) more strongly than **1** (Supporting Information, Table S2). Neutral guests containing five‐ and six‐membered rings were bound by both tetrahedra **1** (*K*_a_=5–15 m ^−1^) and **2** (*K*_a_=91–680 m ^−1^), whereas larger guests were observed to bind only within **2** (*K*_a_ <11 m ^−1^). We infer that the restricted rotation of the phenylenes on the faces of **1**, as noted above, prevented its cavity from adapting to bind the larger neutral guests, while the higher rigidity of **1** enhanced its ability to encapsulate the smaller anionic guests.

**Figure 3:**
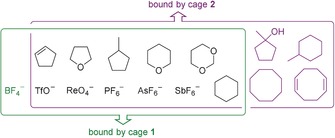
Guests in the overlapping green and purple boxes were observed to bind within cages **1** and **2**, respectively.

Addition of hydrogen bis(trifluoromethanesulfonyl)imide (triflimidic acid, HNTf_2_) to a CD_3_CN solution of tetrahedron **2** led to the progressive disappearance of the cage peaks along with the appearance of new signals corresponding to free 2‐formylpyridine (Supporting Information, Figures S63 and S64), indicating the disassembly of cage **2** upon acid addition. Complete disassembly of **2** was observed following the addition of 10 equivalents of HNTf_2_. In contrast, 30 equivalents of acid were required to induce complete disassembly of cage **1**, with 43 % cage opening observed at 10 equivalents (Supporting Information, Figures S60 and S61), indicating that **1** is more acid‐resistant than **2**. Titration of the base *N*,*N*‐diisopropylethylamine (DIPEA, 0–10 equiv) into the solution of disassembled **2** led to the progressive reformation of the cage, whereas no such regeneration was observed even after the addition of 30 equiv of DIPEA to disassembled **1** (Supporting Information, Figure S62). The salts that build up following the addition of 30 equivalents each of acid and base may thus prevent the reformation of **1**.

The addition of HNTf_2_ (10 equiv) to an equimolar mixture of **1** and **2** in CD_3_CN led to the selective and complete disassembly of **2**, with the ^1^H NMR signals for **1** remaining intact (Supporting Information, Figures S65 and S66). This result further indicates the intrinsically higher stability of **1** towards acid, highlighting the utility of di(2‐pyridyl)ketone in subcomponent self‐assembly. Subsequent addition of DIPEA induced cage **2** to reform (Supporting Information, Figures S67 and S68), regenerating the initial equimolar mixture of the two cages.

Based upon the observations above, we designed the system shown in Figure 4. This system initially consisted of an equimolar mixture of **1** and SbF_6_^−^⊂**2**, prepared separately and then mixed together. As triflimidic acid was progressively added to the mixture (up to 9.0 equiv), the ^1^H NMR signals corresponding to SbF_6_^−^⊂**2** were observed to disappear, accompanied by the appearance of new host–guest peaks of SbF_6_^−^⊂**1** and concomitant disappearance of the peaks for empty **1** (Supporting Information, Figure S69). The addition of acid thus resulted in the selective disassembly of SbF_6_^−^⊂**2** and release of its cargo SbF_6_^−^, which was then encapsulated by cage **1**. Subsequent addition of the base DIPEA (9.0 equiv) generated new ^1^H NMR signals corresponding to empty **2**. The transfer of SbF_6_^−^ from host **2** to host **1** was thus achieved, mediated by acid and base.

**Figure 4:**
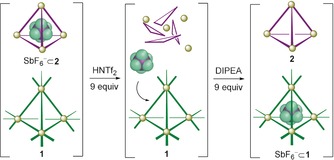
Representation of the transfer of cargo SbF_6_^−^ from cage **2** to cage **1** upon the addition of acid and then base.

The thermodynamically favored cargo transfer from **2** to **1** thus took place over a time scale of minutes via the acid‐base accelerated process shown in Figure 4. The corresponding unassisted process of SbF_6_^−^ delivery from **2** to **1** was slow due to kinetic trapping of the cargo within **2**. After mixing SbF_6_^−^⊂**2** and **1**, only 30 % of the SbF_6_^−^ transferred from **2** into **1** after 3 h, and 80 % of SbF_6_^−^ was delivered after 45 h (Supporting Information, Figure S70). Complete cargo delivery from **2** to **1** thus required the acid–base cycle shown in Figure 4, as the non‐mediated process resulted in kinetic trapping.

The rapid exchange of cargoes between tetrahedra **1** and **2** also occurred between 1,3‐dioxane⊂**1** and SbF_6_^−^⊂**2** (Figure 5). During the progressive addition of triflimidic acid (up to 10 equiv) to the mixture, the ^1^H NMR signals of SbF_6_^−^⊂**2** disappeared as new peaks corresponding to SbF_6_^−^⊂**1** grew in (Supporting Information, Figure S71). The SbF_6_^−^ released from cage **2** following acidification displaced the more‐weakly bound 1,3‐dioxane guest from **1**, forming the new complex SbF_6_^−^⊂**1**. Subsequent addition of DIPEA (10 equiv) brought about the reformation of **2**, which took up the released dioxane from solution to generate 1,3‐dioxane⊂**2**. Thus, by using acid and base as chemical stimuli the cargo SbF_6_^−^ was transferred from cage **2** to cage **1**, and the cargo 1,3‐dioxane was correspondingly moved from the cavity of **1** to that of **2**, with the whole process occurring on a time scale of minutes.

**Figure 5:**
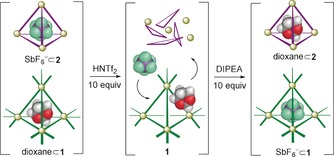
Representation of exchange of cargoes 1,3‐dioxane and SbF_6_^−^ between cages **1** and **2** upon sequential acid–base addition.

This result contrasted with the observation of a control experiment in which no acid–base cycle was carried out. Only 82 % of **1** had taken up SbF_6_^−^ in this case after 81 h, with the other 18 % of **1** binding the original 1,3‐dioxane cargo (Supporting Information, Figure S72).

The strategy for improving the acid resistance of coordination cages developed herein may well allow other functional capsules to be constructed, in turn permitting more complex tasks to be carried out within systems of capsules.^12^ Applications of such systems may include cage‐mediated catalytic processes,3a, 18 wherein the functioning of different cages may be turned on and off without impacting other parts of the system; such processes might be built into complex catalytic relays.^19^ Related systems could also show utility in the selective sequential release of multiple drugs, as well as for new methods of chemical purification involving selective guest uptake and release.

## Conflict of interest

The authors declare no conflict of interest.

## Supporting information

As a service to our authors and readers, this journal provides supporting information supplied by the authors. Such materials are peer reviewed and may be re‐organized for online delivery, but are not copy‐edited or typeset. Technical support issues arising from supporting information (other than missing files) should be addressed to the authors.

Supplementary
