# Supplementary material for: Improved Acid Resistance of a Metal–Organic Cage Enables Cargo Release and Exchange between Hosts
Source: Angew Chem Int Ed Engl. 2020 Mar 18;59(19):7435–8. doi: 10.1002/anie.202001059 (PMC7217015; doi:10.1002/anie.202001059)
Supplement: Supplementary file 1 — Supplementary [file ANIE-59-7435-s001.pdf]

Angewandte Chemie
Eine Zeitschrift der Gesellschaft Deutscher Chemiker

Supporting Information

Improved Acid Resistance of a Metal–Organic Cage Enables Cargo Release and Exchange between Hosts

*Lin Xu[+], Dawei Zhang[+], Tanya K. Ronson, and Jonathan R. Nitschke**

anie_202001059_sm_miscellaneous_information.pdf

Contents

1. Materials and instrumentation 2

2. Synthesis and characterization 3

2.1 Preparation of tetrahedron **1** 3

2.2 Preparation of tetrahedron **2** 9

3. X-ray crystallography 15

4. Volume calculations 23

5. Guest binding studies 24

5.1 Guest binding properties of cage **1** 26

5.2 Guest binding properties of cage **2** 36

6. Acid resistance studies of cages 47

6.1 Acid resistance of cage **1** 47

6.2 Acid resistance of cage **2** 49

6.3 Selective cage disassembly and reassembly among a mixture of cages 50

7. Cargo delivery between cages **1** and **2** 52

8. Cargo exchange between cages **1** and **2** 54

9. References 56

1. Materials and instrumentation

Unless otherwise specified, all reagents were purchased from commercial sources and used as received.

NMR spectra were recorded using a Bruker Avance 400 MHz (^1H, ^{13}C, ^{19}F and 2D experiments) and a Bruker Avance 500 MHz TCI-ATM cryoprobe (^1H NMR) in deuterated solvents. Chemical shifts for ^1H, ^{13}C, and ^{19}F NMR are reported in ppm on the δ scale; ^1H and ^{13}C were referenced to the residual solvent peak. ^{19}F was reported relative to hexafluorobenzene at -164.9 ppm. Coupling constants (J) are reported in Hz. The following abbreviations are used to describe signal multiplicity for ^1H, ^{13}C and ^{19}F NMR spectra: s for singlet, d for doublet, t for triplet, dd for doublet of doublets; dt for doublet of triplets, and m for multiplet.

Centrifugation of cage samples was carried out using a Grant-Bio LMC-3000 low speed benchtop centrifuge. Low resolution electrospray ionization mass spectrometry was undertaken on a Micromass Quattro LC mass spectrometer (cone voltage 10-30 eV; desolvation temp. 40 °C; ionization temp. 40 °C) infused from a Harvard syringe pump at a rate of 10 μL/min. High resolution electrospray ionisation mass spectrometry (ESI-MS) was carried out by the EPSRC UK National Mass Spectrometry Facility at Swansea University on a LTQ Orbitrap XL hybrid ion trap-orbitrap mass spectrometer.

2. Synthesis and characterization

2.1 Preparation of tetrahedron 1

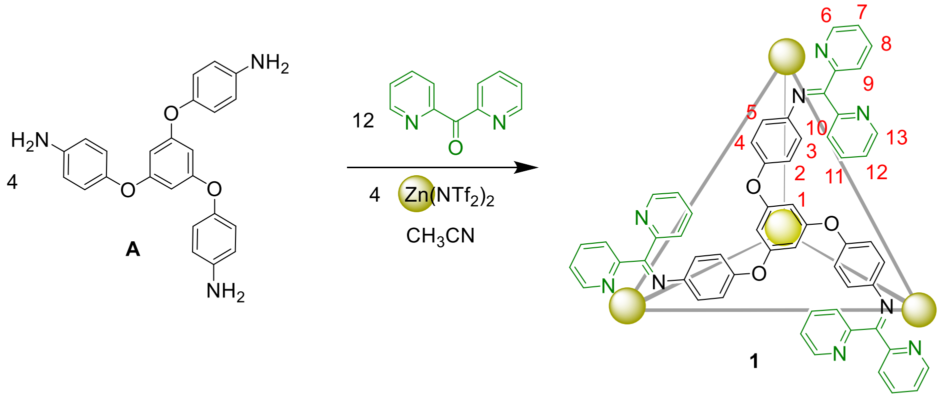

Scheme S1. Subcomponent self-assembly of **1**.

1,3,5-tris(4'-aminophenyloxy)benzene **A**[1] (47 mg, 4.0 equiv), di(2-pyridyl)ketone (66 mg, 12 equiv) and $Zn(NTf_2)_2$ (75 mg, 4.0 equiv.) were combined in CH_3CN (10 mL) and stirred at 70 °C overnight. The solvent was evaporated and diethyl ether was then added, the residue resuspended and then centrifuged and the diethyl ether decanted. This was repeated three times with fresh diethyl ether. The residue was then dried in vacuo to afford the solid product as a fine gray powder (170 mg, 93%). **^{1}H NMR** (CD_3CN, 400 MHz, 298 K): δ (ppm) 8.52 (d, J = 4.8 Hz, 12H, *H13*), 8.48 (d, J = 4.8 Hz, 12H, *H6*), 8.41 (t, J = 8.0 Hz, 12H, *H8*), 8.07 (dd, J = 5.2 Hz, 7.6 Hz, 12H, *H7*), 7.75 (d, J = 7.6 Hz, 12H, *H9*), 7.54 (dt, J = 1.2 Hz, 7.6 Hz, 12H, *H11*), 7.27 (dd, J = 4.8 Hz, 7.6 Hz, 12H, *H12*), 7.10 (dd, J = 2.4 Hz, 8.4 Hz, 12H, *H4*), 7.02 (d, J = 7.6 Hz, 12H, *H10*), 6.15 (dd, J = 2.4 Hz, 8.4 Hz, 12H, *H2*), 6.07 (dd, J = 2.0 Hz, 8.4 Hz, 12H, *H5*), 5.77 (s, 12H, *H1*), 5.10 (dd, J = 2.4 Hz, 8.8 Hz, 12H, *H3*). **^{13}C NMR** (CD_3CN. 100 MHz, 298 K): δ (ppm) 171.6, 162.6, 153.7, 151.1, 151.0, 150.3, 148.1, 144.0, 143.8, 137.7, 132.1, 131.7, 126.2, 125.7, 125.4, 125.1, 124.7, 121.7, 98.9. **^{19}F NMR** (CD_3CN, 376 MHz, 298 K, referenced to C_6F_6): δ (ppm) -80.52. **ESI-MS**: *m/z* 481.6 $[M]^{8+}$, 590.3 $[M+1Tf_2N^-]^{7+}$, 735.4 $[M+2Tf_2N^-]^{6+}$, 938.5 $[M+3Tf_2N^-]^{5+}$, 1243.2 $[M+4Tf_2N^-]^{4+}$.

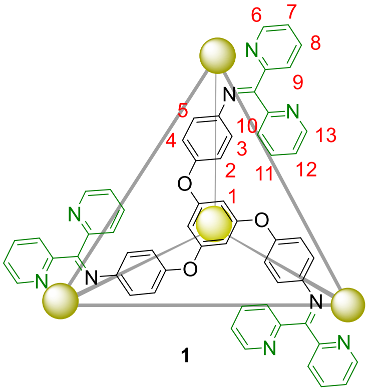

1

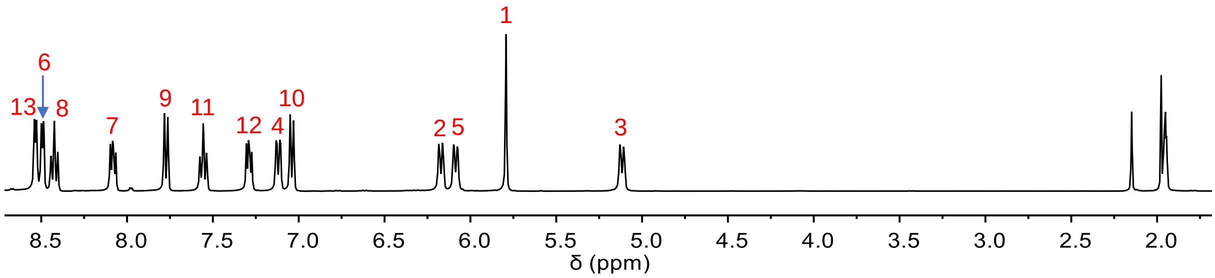

Figure S1. ^{1}H NMR spectrum of (CD_3CN, 400 MHz, 298 K) **1**.

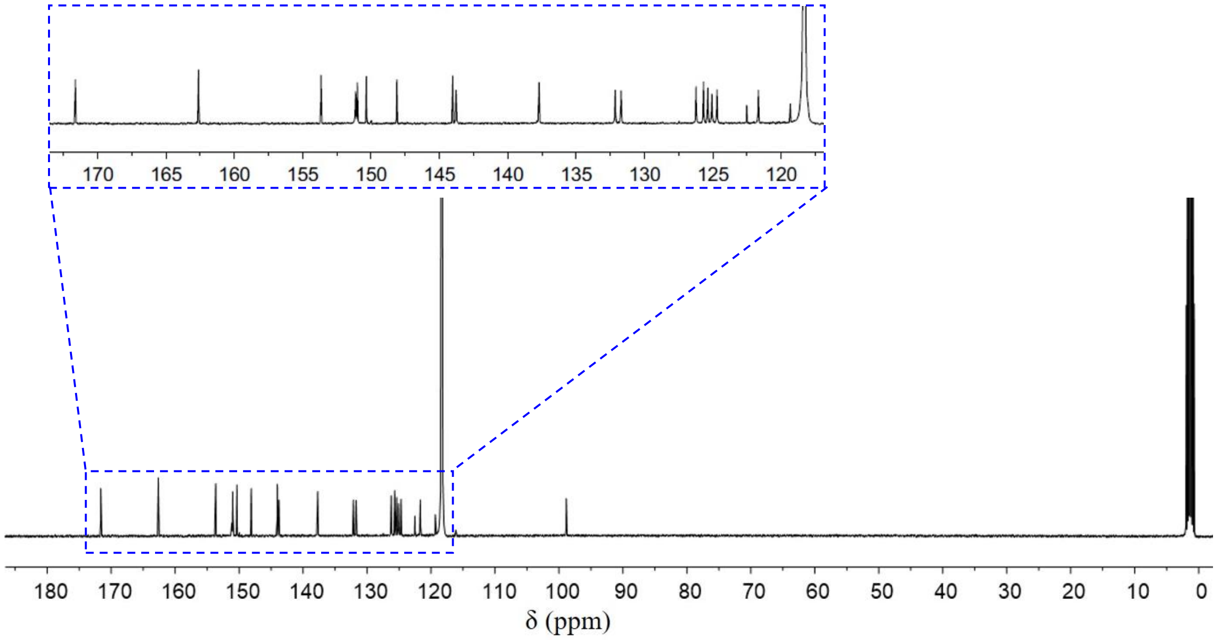

Figure S2. ^{13}C NMR spectrum (CD_3CN, 100 MHz, 298 K) of **1**.

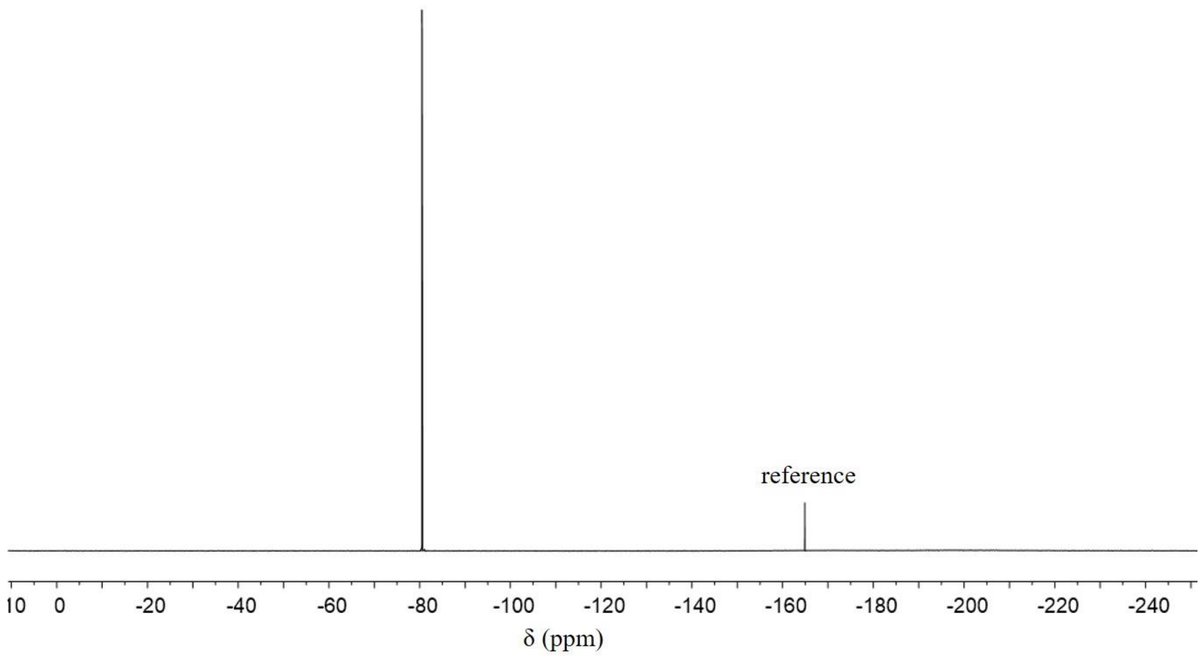

Figure S3. ^{19}F NMR spectrum (CD_3CN, 376 MHz, 298 K, referenced to C_6F_6) of **1**.

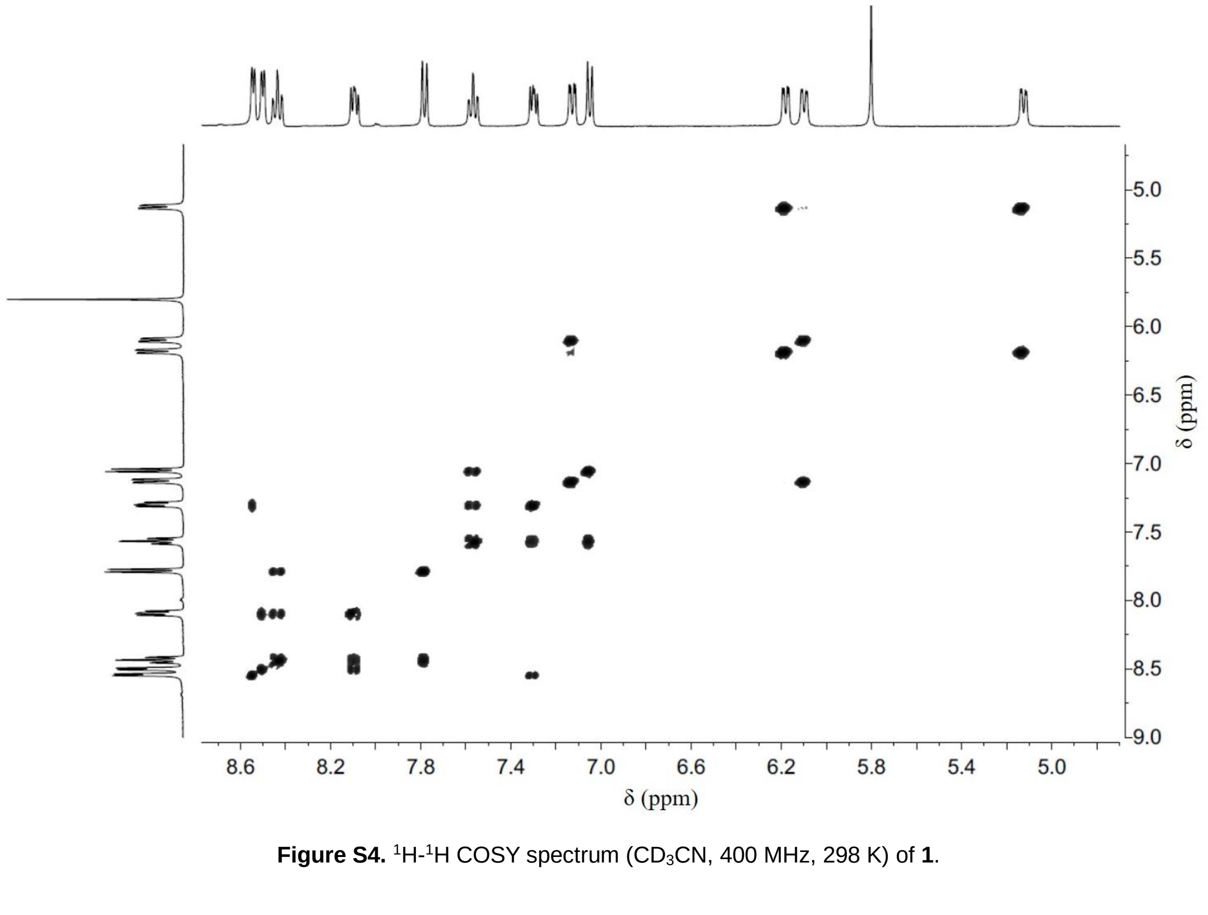

Figure S4. ^{1}H-^{1}H COSY spectrum (CD_3CN, 400 MHz, 298 K) of **1**.

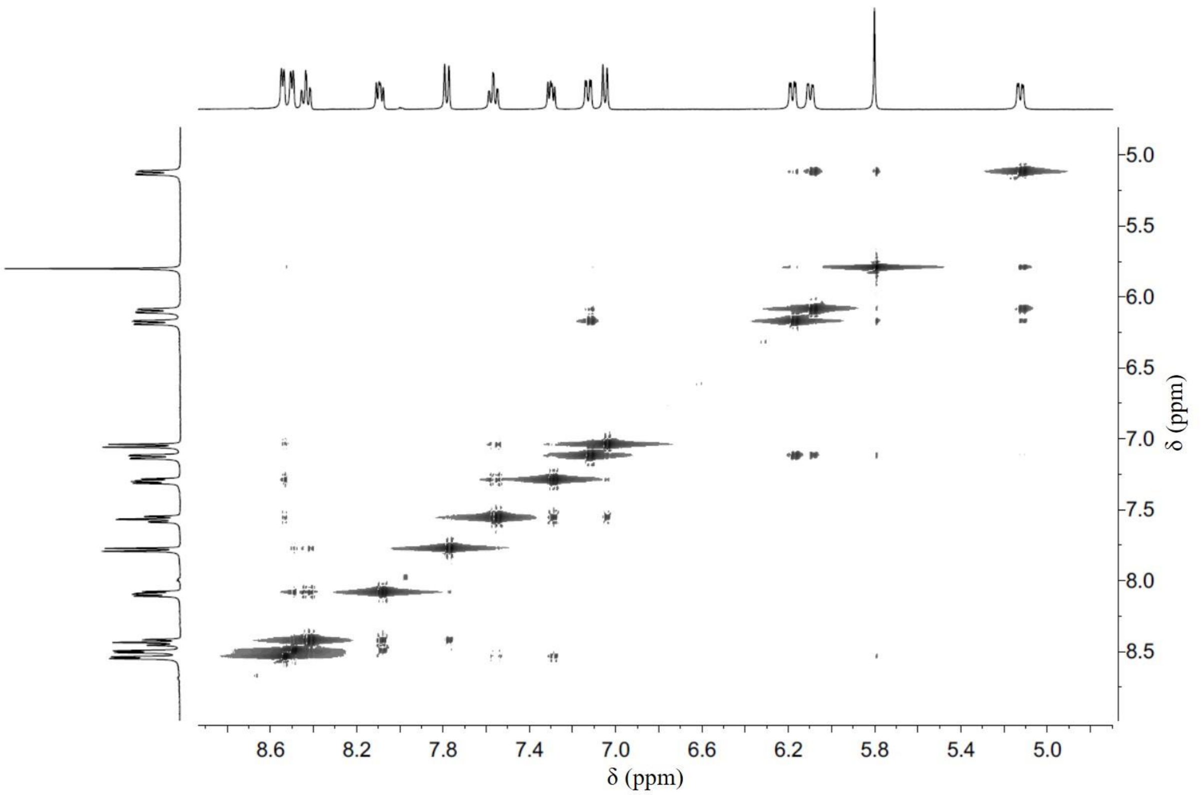

Figure S5. ^{1}H-^{1}H NOESY spectrum (CD_3CN, 400 MHz, 298 K) of **1**.

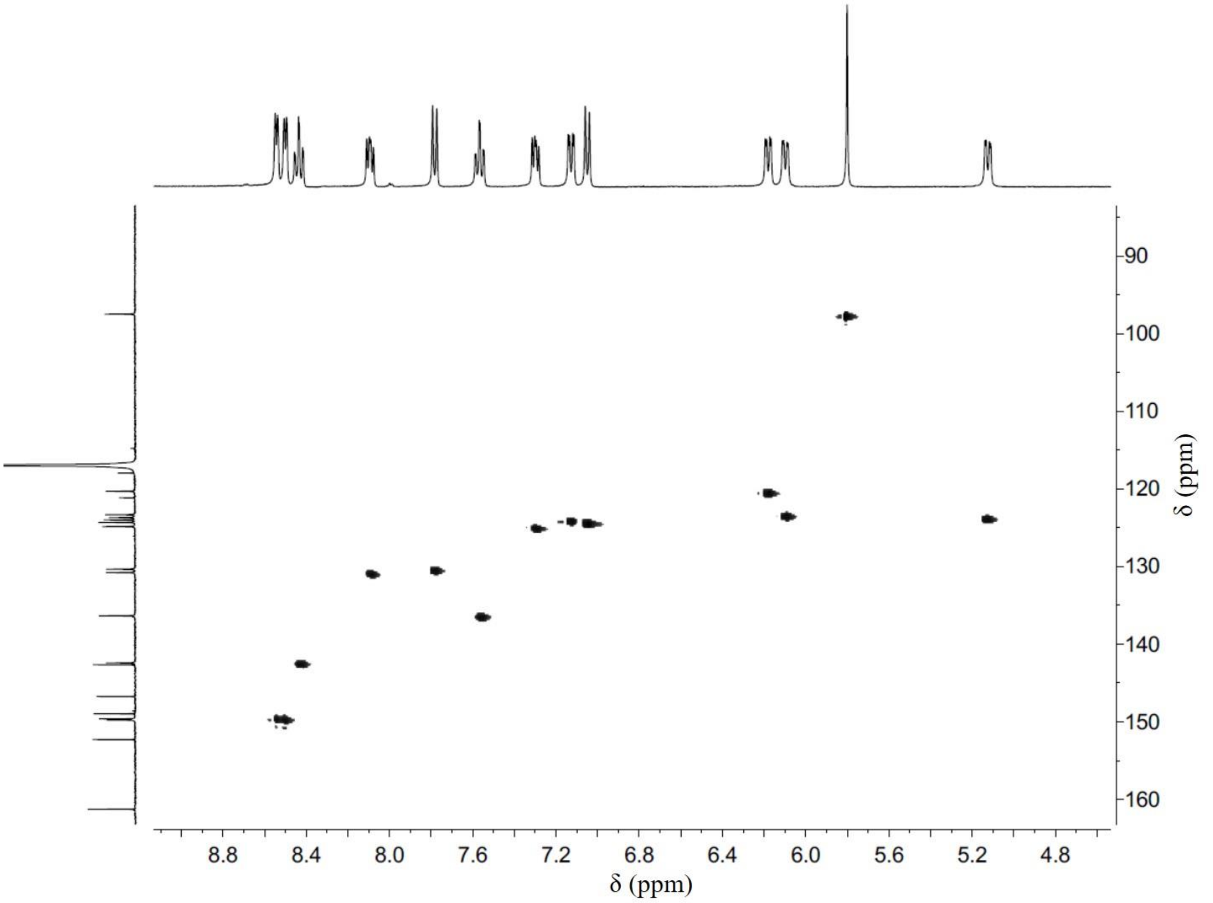

Figure S6. ^{1}H-^{13}C HSQC spectrum (CD_3CN, 400 MHz, 298 K) of **1**.

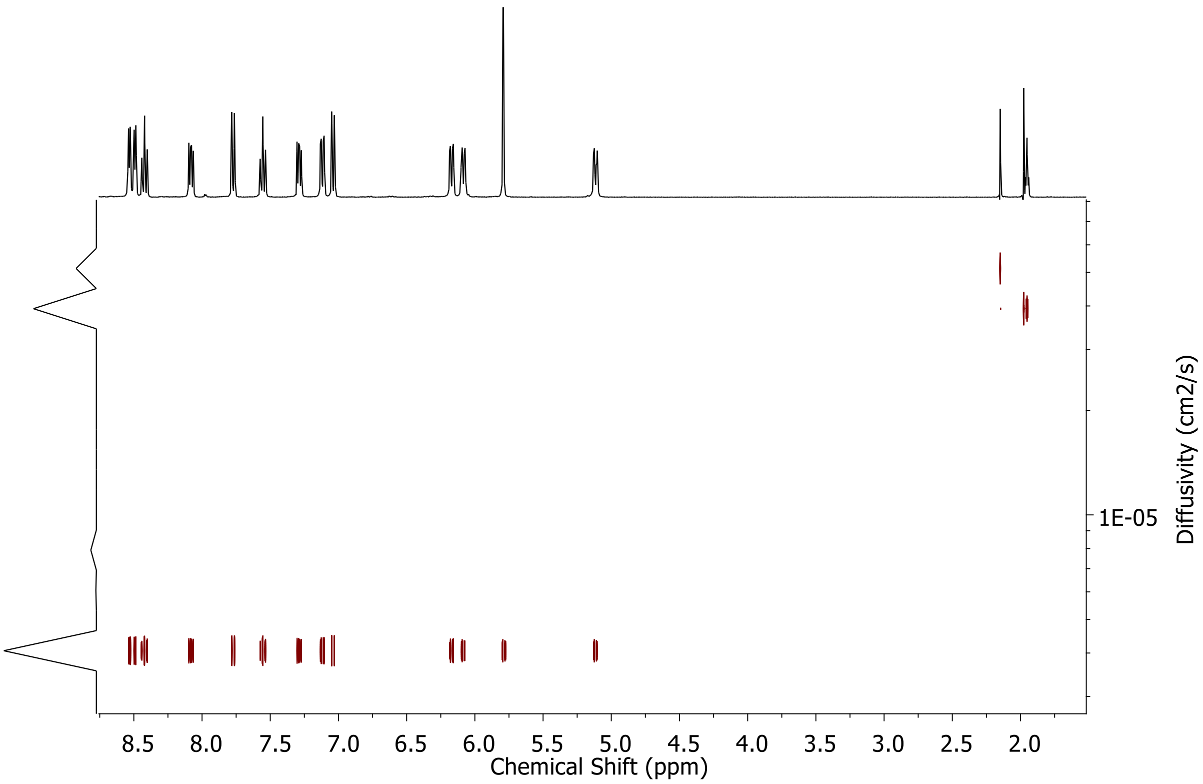

Figure S7. ^{1}H DOSY spectrum (CD_3CN, 400 MHz, 298 K) of **1**. The diffusion coefficient of **1** in CD_3CN was measured to be 4.08×10^{-6} cm^2 s^{-1}.

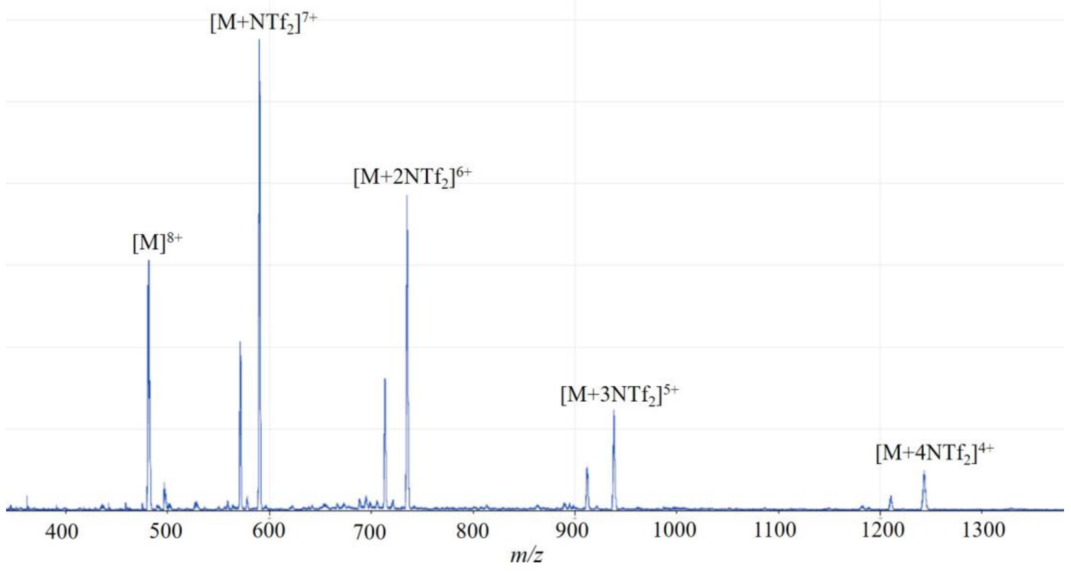

Figure S8. Low-resolution ESI-mass spectrum of **1**.

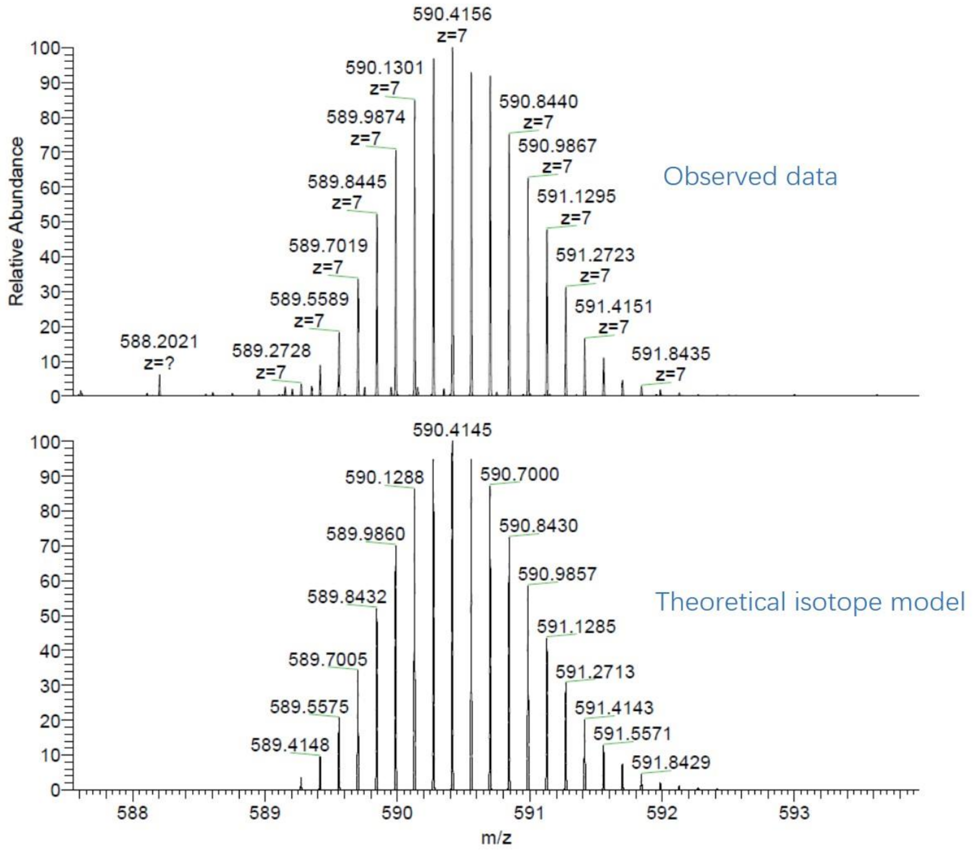

Figure S9. High-resolution ESI-mass spectrometry analysis of **1** showing the +7 peak.

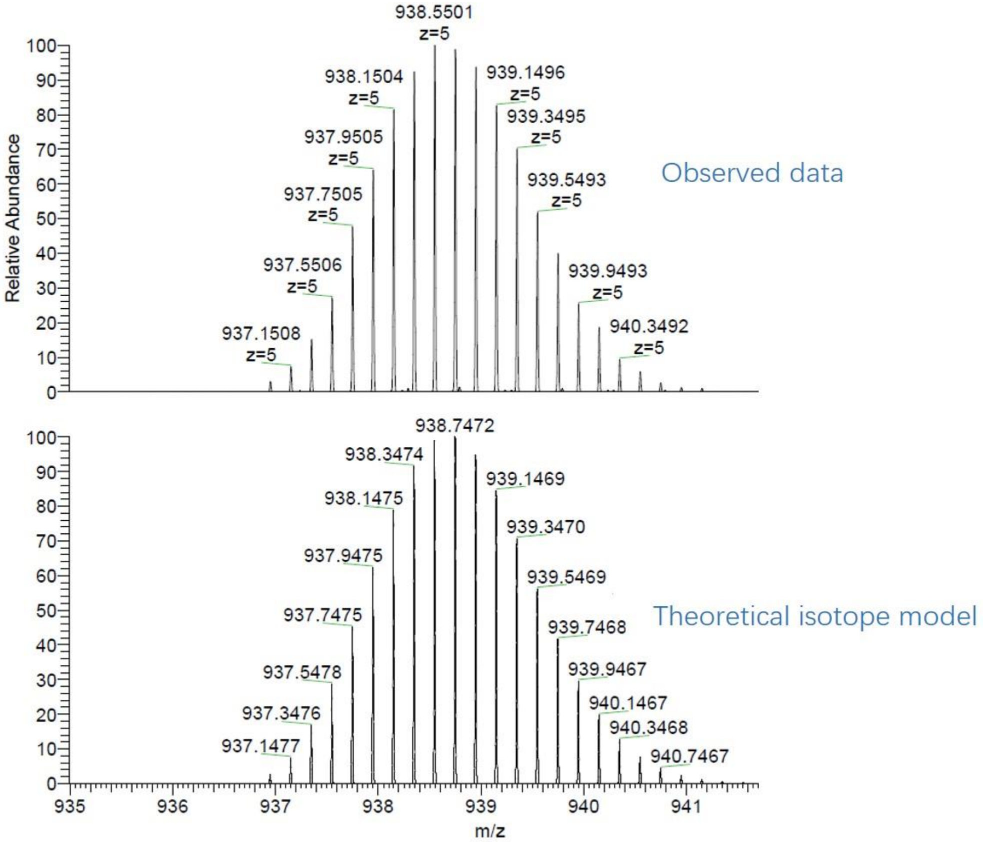

Figure S10. High-resolution ESI-mass spectrometry analysis of **1** showing the +5 peak.

2.2 Preparation of tetrahedron 2

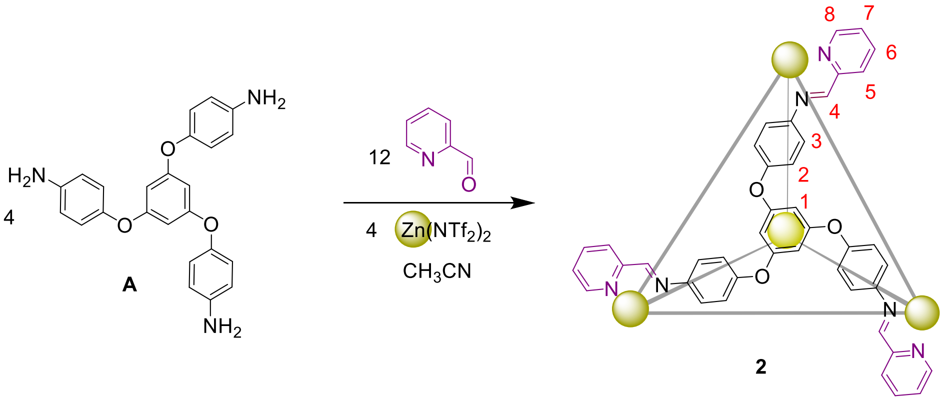

Scheme S2. Subcomponent self-assembly of **2**.

1,3,5-tris(4'-aminophenyloxy)benzene **A** (106 mg, 4.0 equiv), 2-formylpyridine (85 mg, 12 equiv) and $Zn(NTf_2)_2$ (166 mg, 4.0 equiv) were combined in CH_3CN (20 mL) and stirred at 70 °C overnight. The solvent was evaporated and diethyl ether was then added. The residue resuspended and then centrifuged, and the diethyl ether decanted. This was repeated three times with fresh diethyl ether. The residue was then dried in vacuo to afford the solid product as a fine yellow powder (302 mg, 88%). **^{1}H NMR** (CD_3CN, 400 MHz, 298 K): δ (ppm) 8.61 (s, 12H, *H4*), 8.47 (t, *J* = 7.6 Hz, 12H, *H7*), 8.25 (d, *J* = 8.0 Hz, 12H, *H8*), 8.01 (d, *J* = 4.4 Hz, 12H, *H5*), 7.92 (t, *J* = 5.6 Hz, 12H, *H6*), 7.13 (d, *J* = 8.0 Hz, 24H, *H2*), 6.31 (s, 12H, *H1*), 6.19 (d, *J* = 8.0 Hz, 24H, *H3*). **^{13}C NMR** (CD_3CN, 100 MHz, 298 K): δ (ppm) 167.0, 161.9, 154.3, 150.6, 147.1, 146.5, 143.8, 132.0, 124.4, 124.0, 122.5, 119.3, 98.3. **^{19}F NMR** (CD_3CN, 376 MHz, 298 K, referenced to C_6F_6): δ (ppm) -80.55. **ESI-MS**: *m/z* 458.3 $[M+1Tf_2N^-]^{7+}$, 581.4 $[M+2Tf_2N^-]^{6+}$, 753.5 $[M+3Tf_2N^-]^{5+}$, 1012.1 $[M+4Tf_2N^-]^{4+}$, 1442.7 $[M+5Tf_2N^-]^{3+}$.

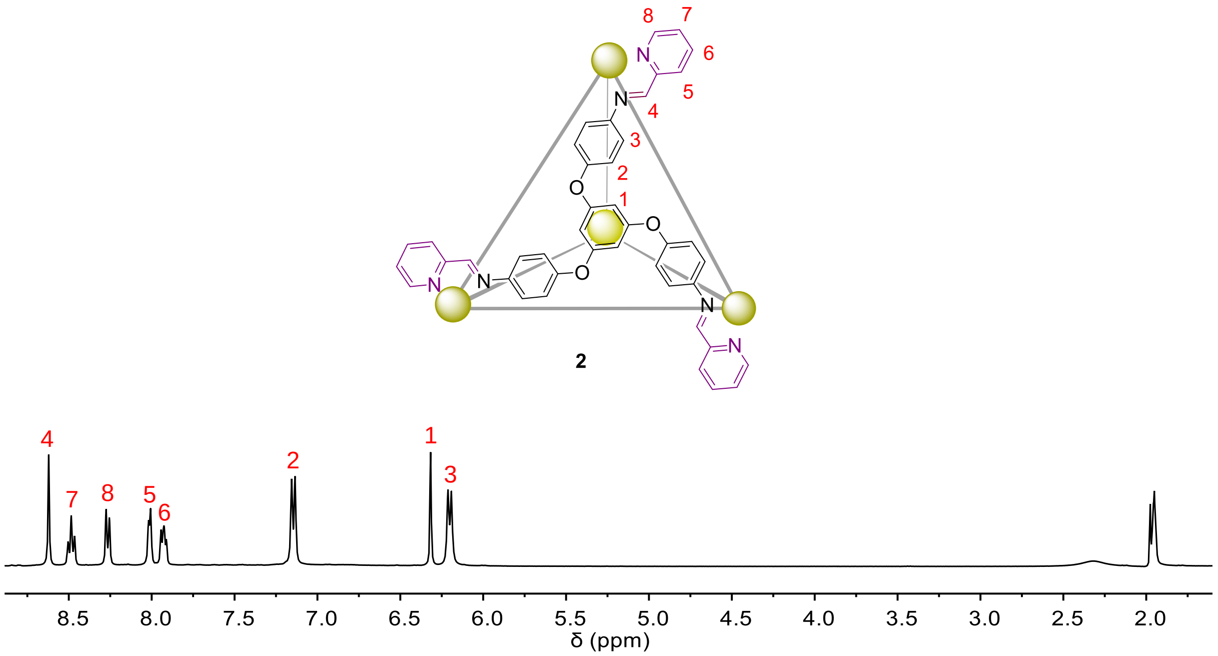

Figure S11. ^{1}H NMR spectrum (CD_3CN, 400 MHz, 298 K) of **2**.

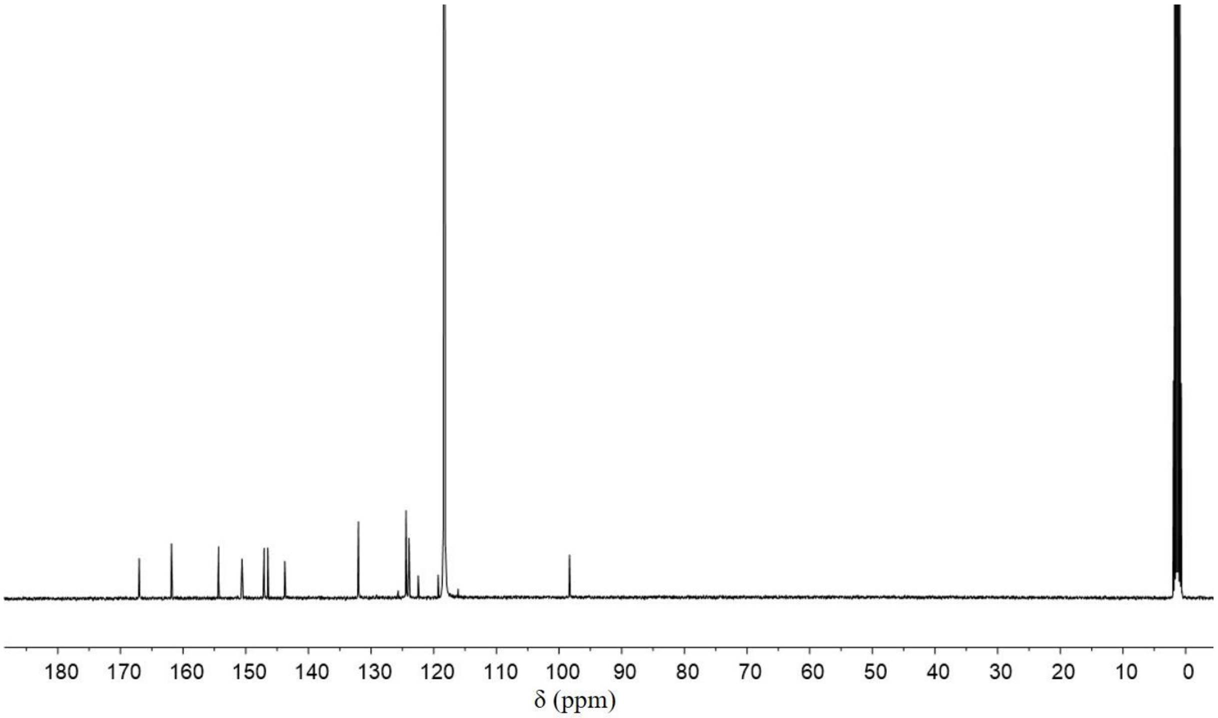

Figure S12. ^{13}C NMR spectrum (CD_3CN, 100 MHz, 298 K) of **2**.

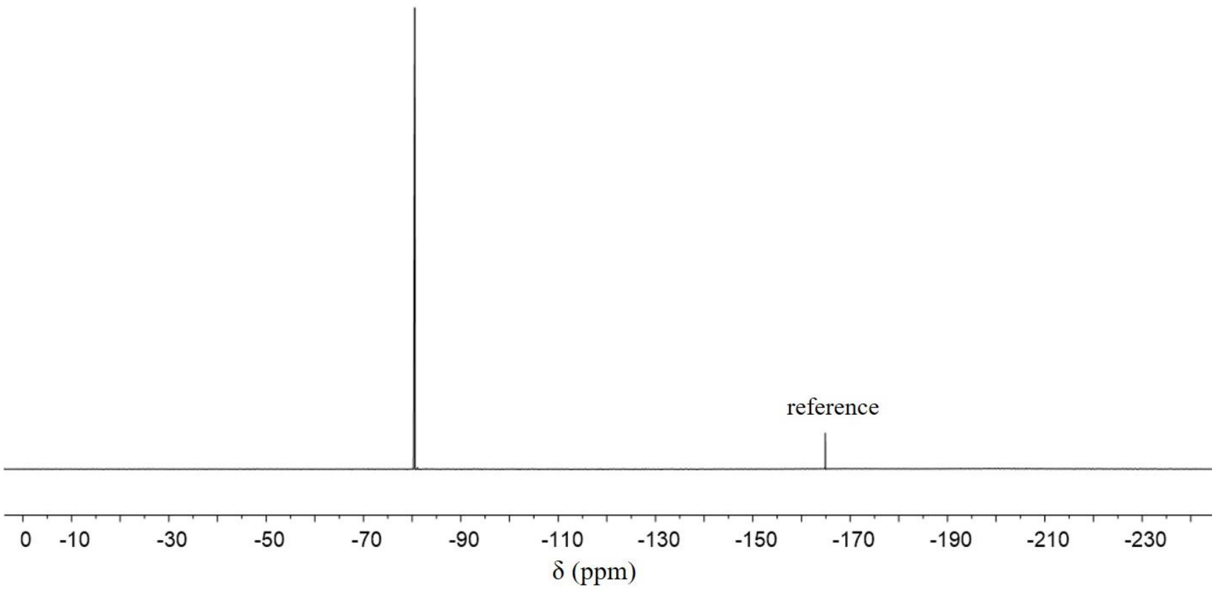

Figure S13. ^{19}F NMR spectrum (CD_3CN, 376 MHz, 298 K, referenced to C_6F_6) of **2**.

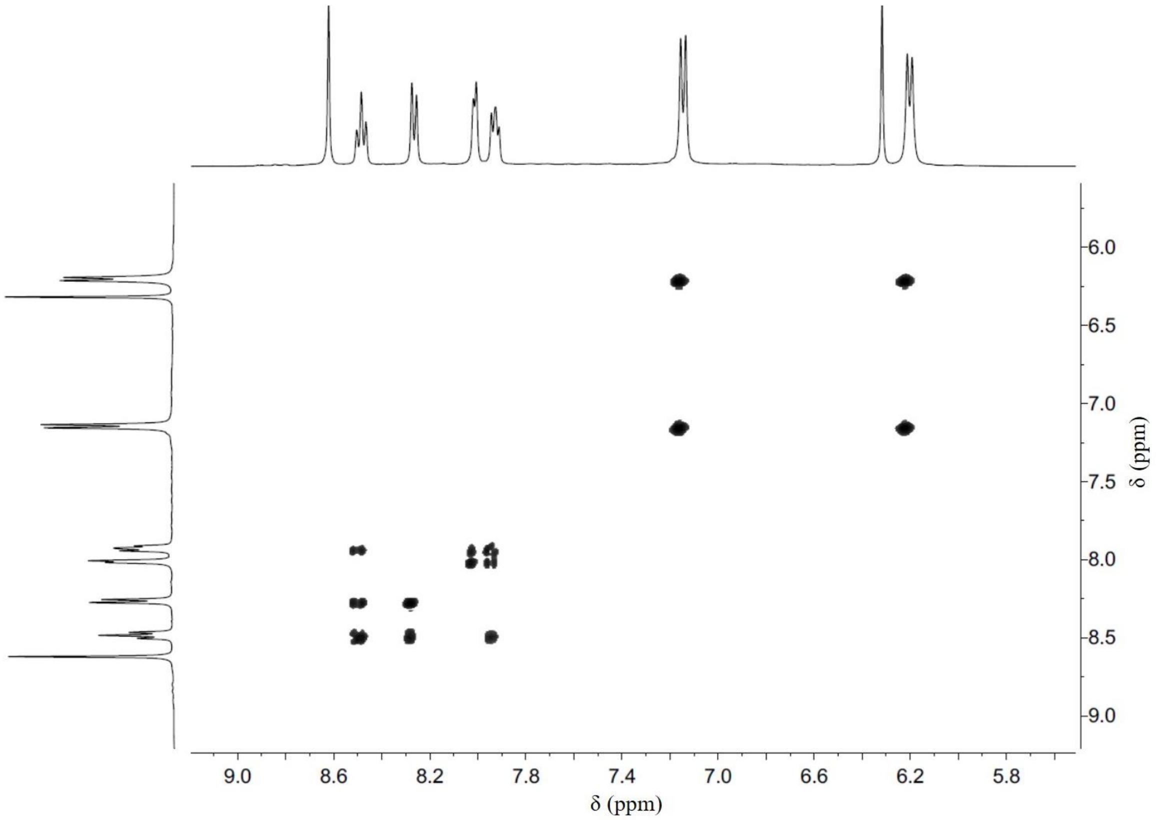

Figure S14. ^{1}H-^{1}H COSY spectrum (CD_3CN, 400 MHz, 298 K) of **2**.

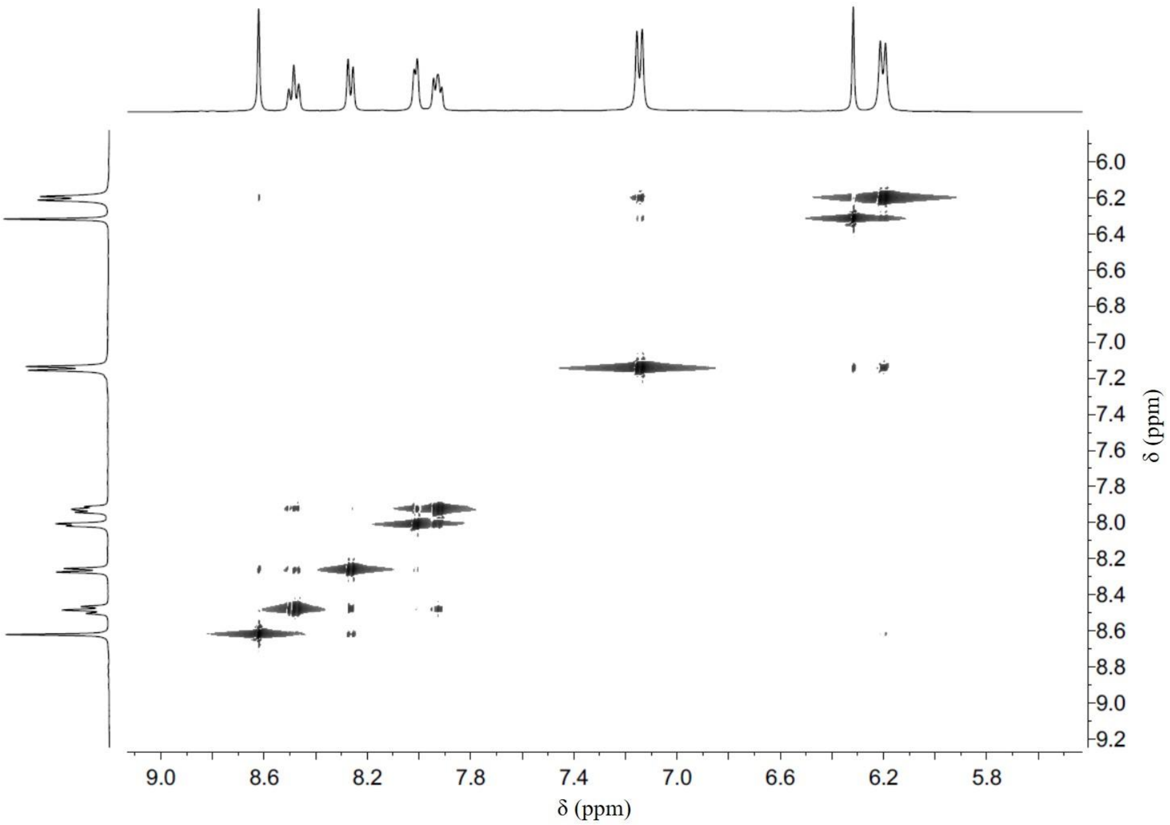

Figure S15. ^{1}H-^{1}H NOESY spectrum (CD_3CN, 400 MHz, 298 K) of **2**.

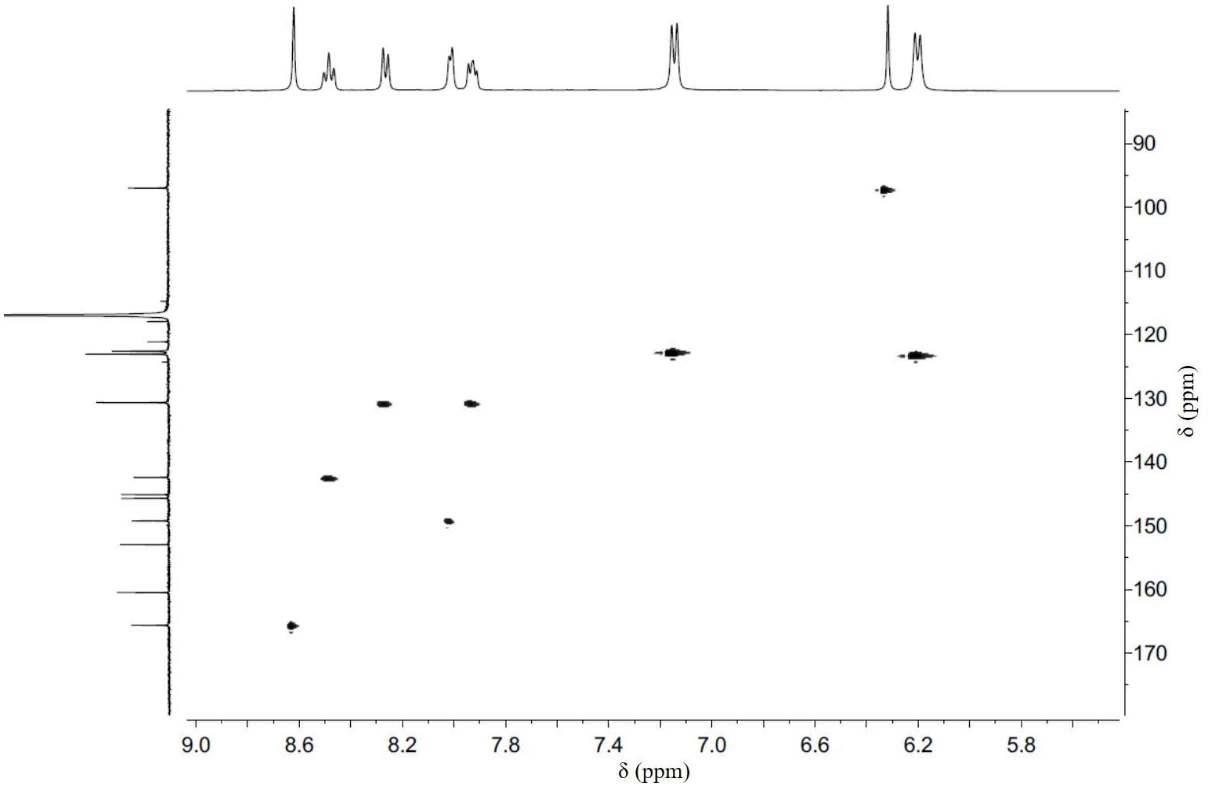

Figure S16. ^{1}H-^{13}C HSQC spectrum (CD_3CN, 400 MHz, 298 K) of **2**.

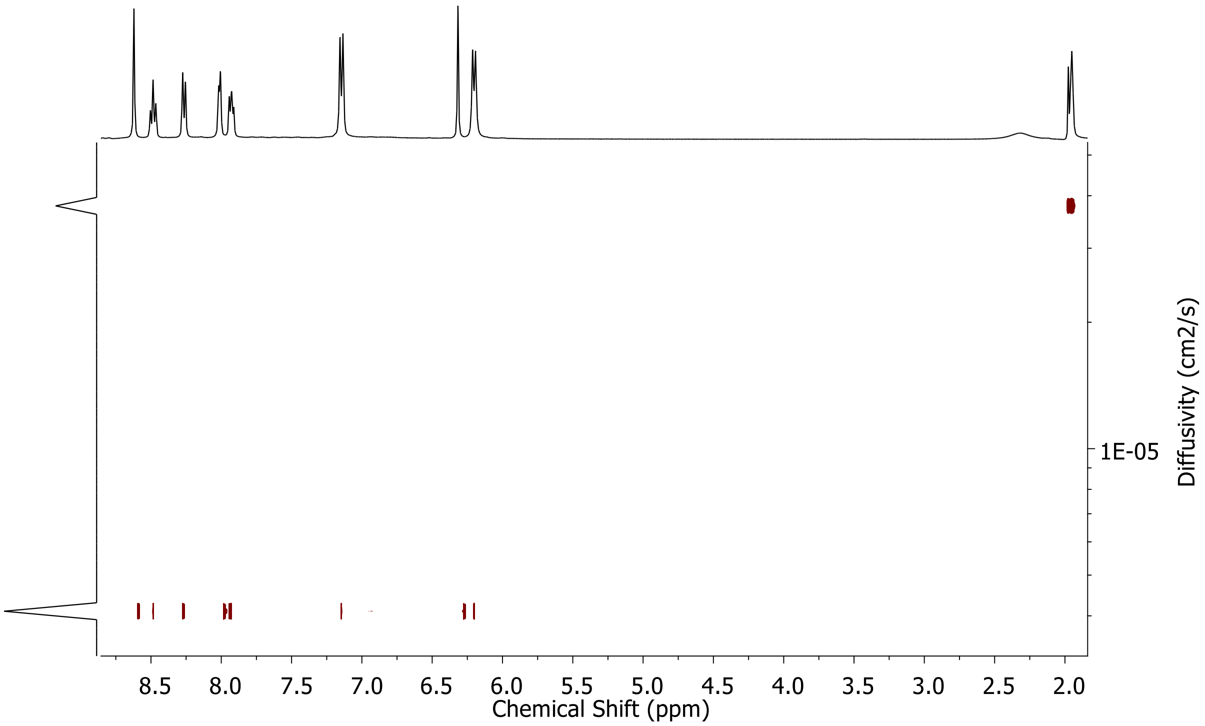

Figure S17. ^{1}H DOSY spectrum (CD_3CN, 400 MHz, 298 K) of **2**. The diffusion coefficient of **2** in CD_3CN was measured to be 4.13×10^{-6} cm^2 s^{-1}.

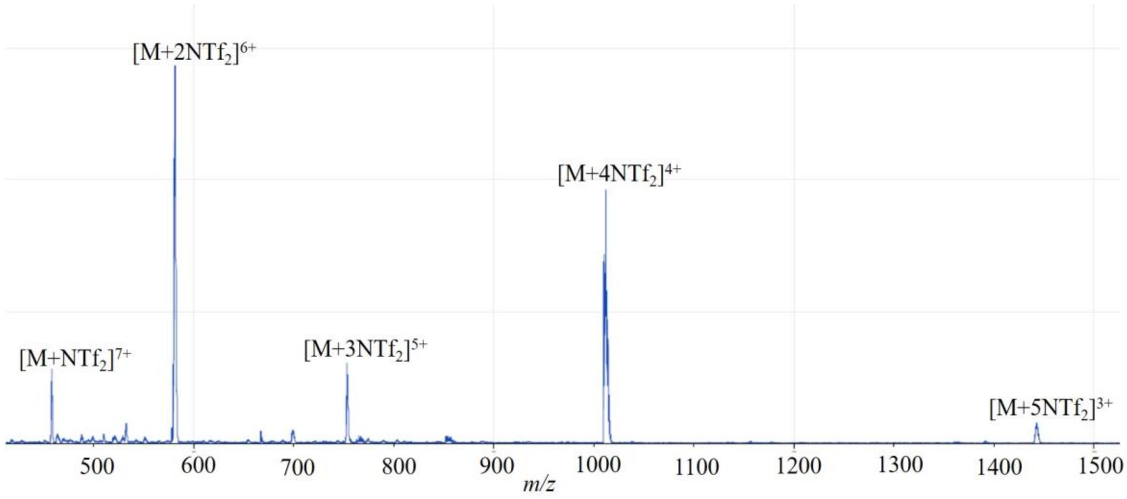

Figure S18. Low-resolution ESI-mass spectrum of **2**.

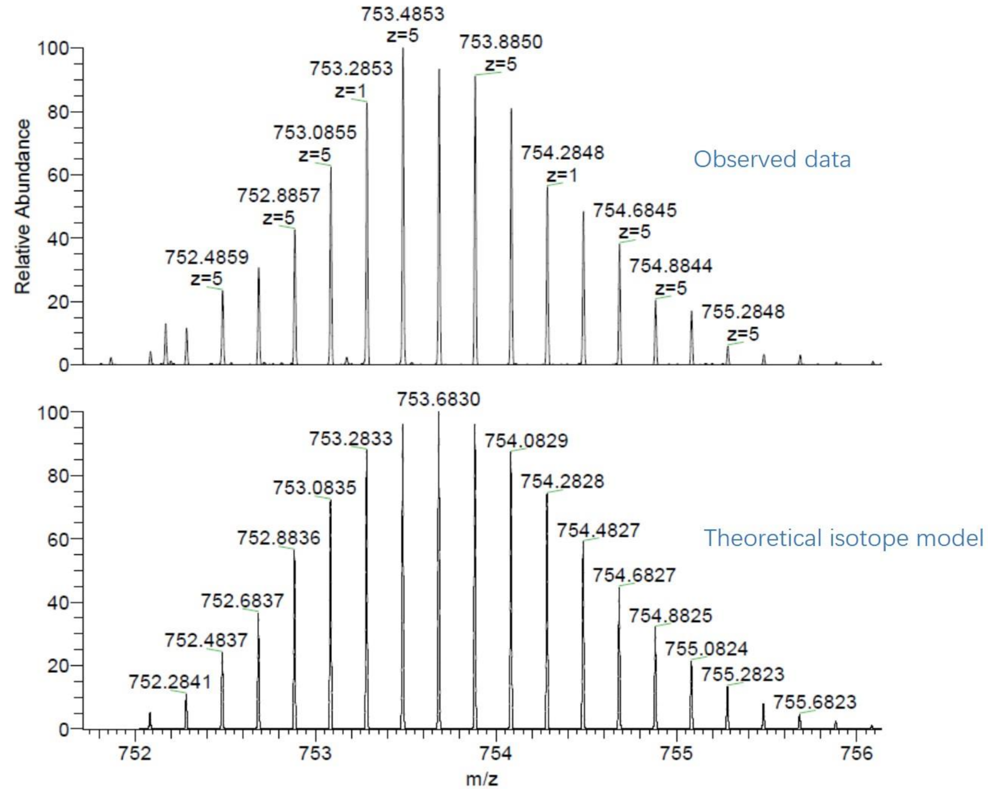

Figure S19. High-resolution ESI-mass spectrometry analysis of **2** showing the +5 peak.

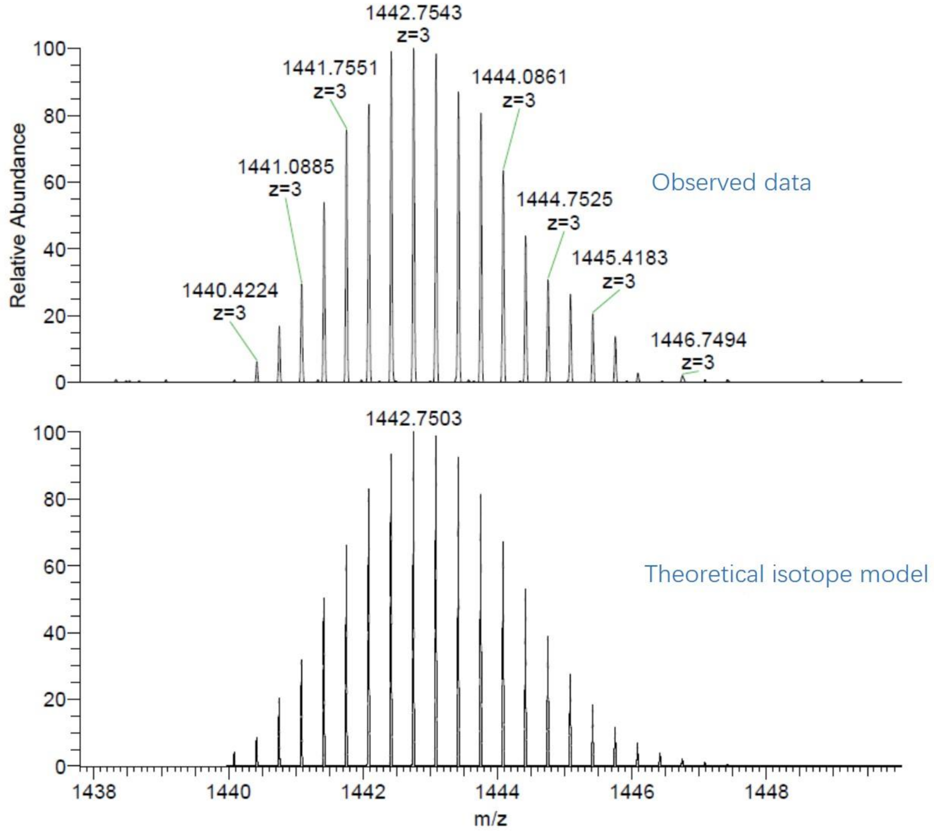

Figure S20. High-resolution ESI-mass spectrometry analysis of **2** showing the +3 peak.

3. X-ray crystallography

Data were with collected either using a Bruker D8 VENTURE equipped with high-brilliance IμS Cu-Kα radiation (1.54178 Å), with ω and ψ scans at 180(2) K or at Beamline I19 of Diamond Light Source employing silicon double crystal monochromated synchrotron radiation (0.6889 Å) with ω scans at 100(2) K.[2] Data integration and reduction were undertaken with Xia2[3] or CrysalisPro.[4] Subsequent computations were carried out using the WinGX-32 graphical user interface.[5] Empirical absorption corrections were applied to the data using the AIMLESS tool[6] in the CCP4 suite.[7] Structures were solved by direct methods using SHELXT-2013[8] then refined and extended with SHELXL-2013.[9] In general, non-hydrogen atoms with occupancies greater than 0.5 were refined anisotropically. Carbon-bound hydrogen atoms were included in idealised positions and refined using a riding model. Disorder was modelled using standard crystallographic methods including constraints, restraints and rigid bodies where necessary. Crystallographic data along with specific details pertaining to the refinement follow. Crystallographic data have been deposited with the CCDC (1585972-1585977).

$[BF_4\subset\mathbf{1}]\cdot 7BF_4\cdot 11.25MeCN\cdot 0.75Et_2O$ [+ solvent]

Formula $C_{253.50}H_{197.25}B_8F_{32}N_{47.25}O_{12.75}Zn_4$, *M* 5065.28, Triclinic, space group P -1 (#2), *a* 23.5643(7), *b* 23.8811(8), *c* 24.3617(7) Å, α 89.309(2), β 70.3080(10), γ 74.099(2)°, *V* 12364.8(7) Å^3, D_c 1.360 g cm^{-3}, *Z* 2, crystal size 0.450 by 0.410 by 0.050 mm, colour yellow, habit plate, temperature 180(2) Kelvin, λ(CuKα) 1.54178 Å, μ(CuKα) 1.236 mm^{-1}, T(SADABS)$_{min,max}$ 0.5647, 0.7524, $2\theta_{max}$ 128.22, *hkl* range -27 27, -27 27, -28 28, *N* 138778, N_{ind} 40527(R_{merge} 0.0569), N_{obs} 29220(I > 2σ(I)), N_{var} 3491, residuals* $R1(F)$ 0.0723, $wR2(F^2)$ 0.2139, GoF(all) 1.015, $\Delta\rho_{min,max}$ -0.760, 1.338 e$^-$ Å^{-3}.

*$R1 = \Sigma||F_o| - |F_c||/\Sigma|F_o|$ for $F_o > 2\sigma(F_o)$; $wR2 = (\Sigma w(F_o^2 - F_c^2)^2/\Sigma(wF_c^2)^2)^{1/2}$ all reflections

$w=1/[\sigma^2(F_o^2)+(0.1075P)^2+28.0515P]$ where $P=(F_o^2+2F_c^2)/3$

Specific refinement details:

The crystals of $[BF_4\subset\mathbf{1}]\cdot 7BF_4\cdot 11.25MeCN\cdot 0.75Et_2O$ were grown by diffusion of diethyl ether into an acetonitrile solution of $[Zn_4L_4]\cdot 8NTf_2$ containing excess of Me_4NBF_4. The crystals immediately lost solvent after removal from the mother liquor and rapid handling prior to flash cooling in the cryostream was required to collect data. These measures and the use of a high intensity laboratory source allowed data to be collected to ca. 0.86 Å resolution. The asymmetric unit was found to contain one complete Zn_4L_4 assembly and associated counterions and solvent molecules. Bond lengths and angles within pairs of chemically identical organic ligands were restrained to be similar to each other and thermal

parameter restraints (SIMU, RIGU) were applied to all atoms except for zinc.

Two of the non-coordinated pyridine rings were modelled as disordered over two or three locations with bond length and thermal parameter restraints applied in order to obtain a reasonable refinement. Several of the anions within the structure also show evidence of disorder. Three tetrafluoroborate anions were modelled as disordered over up to five locations. Several solvent molecules were also modelled as disordered over multiple locations and/or with partial occupancy. Substantial bond length and thermal parameter restraints were applied to facilitate a reasonable refinement of the disordered anions and solvent molecules and most low occupancy disordered moieties were modelled with isotropic thermal parameters.

The SQUEEZE[10] function of PLATON[11] was employed to remove the contribution of the electron density associated with a small amount of highly disordered solvent, which gave a potential solvent accessible void of 230 Å^3 per unit cell (a total of approximately 62 electrons). Since the diffuse solvent molecule(s) could not be assigned conclusively to acetonitrile or diethyl ether only those solvent molecules that could be modelled with discrete atom positions are included in the formula.

[OTf⊂1]·7OTf·9.25MeCN·0.75Et$_2$O·0.5H$_2$O

Formula $C_{257.50}H_{192.25}F_{24}N_{45.25}O_{37.25}S_8Zn_4$, *M* 5490.26, Triclinic, space group P -1 (#2), *a* 24.1901(8), *b* 25.7729(8), *c* 26.3617(8) Å, α 69.9200(10), β 64.6050(10), γ 85.694(2)°, *V* 13894.1(8) Å^3, D_c 1.312 g cm^{-3}, *Z* 2, crystal size 0.420 by 0.310 by 0.190 mm, colour yellow, habit block, temperature 180(2) Kelvin, λ(CuKα) 1.54178 Å, μ(CuKα) 1.717 mm^{-1}, *T*(SADABS)$_{min,max}$ 0.5400, 0.7528, 2θ_{max} 133.51, *hkl* range -28 28, -30 30, -31 18, *N* 132332, N_{ind} 47955(R_{merge} 0.0379), N_{obs} 39212(I > 2σ(I)), N_{var} 3876, residuals* *R*1(*F*) 0.0860, *wR*2(F^2) 0.2451, GoF(all) 1.050, $\Delta\rho_{min,max}$ -0.866, 1.067 e$^-$ Å^{-3}.

$^*R1 = \Sigma||F_o| - |F_c||/\Sigma|F_o|$ for $F_o > 2\sigma(F_o)$; $wR2 = (\Sigma w(F_o^2 - F_c^2)^2/\Sigma(wF_c^2)^2)^{1/2}$ all reflections

$w=1/[\sigma^2(F_o^2)+(0.1125P)^2+38.5273P]$ where $P=(F_o^2+2F_c^2)/3$

Specific refinement details:

The crystals of [OTf⊂**1**]·7OTf·9.25MeCN·0.75Et$_2$O·0.5H$_2$O were grown by diffusion of diethyl ether into an acetonitrile solution of the complex. The crystals immediately lost solvent after removal from the mother liquor and rapid handling prior to flash cooling in the cryostream was required to collect data. The asymmetric unit was found to contain one complete Zn$_4$L$_4$ assembly and associated counterions and solvent molecules. Bond lengths and angles within pairs of chemically identical organic ligands were restrained to be similar to each other and thermal parameter restraints (SIMU, RIGU) were applied to all atoms except for zinc.

The anions within the structure show evidence of substantial disorder. Seven triflate anions were modelled as disordered over two locations and multiple anions were modelled with partial occupancy. Several solvent molecules were also modelled as disordered over multiple locations and/or with partial occupancy. Substantial bond length and thermal parameter restraints were applied to facilitate a reasonable refinement of the disordered triflate anions and solvent molecules and most low occupancy disordered groups were modelled with isotropic thermal parameters.

Further reflecting the solvent loss and poor diffraction properties there is a significant amount of void volume in the lattice containing smeared electron density from disordered solvent. Consequently the SQUEEZE[10] function of PLATON[11] was employed to remove the contribution of the electron density associated with this highly disordered solvent, which gave a potential solvent accessible void of 1233.7 Å^3 per unit cell (a total of approximately 308 electrons). Since the diffuse solvent molecules could not be assigned conclusively to acetonitrile or diethyl ether only those solvent molecules that could be modelled with discrete atom positions are included in the formula.

[ReO_4⊂1]·7 NTf_2·MeCN [+ solvent]

Formula $C_{244}H_{159}F_{42}N_{44}O_{44}ReS_{14}Zn_4$, *M* 6105.66, Monoclinic, space group P 21/c (#14), *a* 38.5393(14), *b* 21.5957(8), *c* 36.5384(13) Å, β 110.477(2), *V* 28488.7(18) Å^3, D_c 1.424 g cm^{-3}, *Z* 4, crystal size 0.330 by 0.270 by 0.050 mm, colour yellow, habit plate, temperature 180(2) Kelvin, λ(CuKα) 1.54178 Å, μ(CuKα) 3.031 mm^{-1}, T(SADABS)$_{min,max}$ 0.4235, 0.7493, $2\theta_{max}$ 95.02, *hkl* range -36 36, -15 20, -26 34, *N* 79326, N_{ind} 25671(R_{merge} 0.0510), N_{obs} 18676(I > 2σ(I)), N_{var} 3872, residuals* $R1(F)$ 0.1296, $wR2(F^2)$ 0.3743, GoF(all) 1.078, $\Delta\rho_{min,max}$ -0.697, 1.153 e$^-$ Å^{-3}.

*$R1 = \Sigma||F_o| - |F_c||/\Sigma|F_o|$ for $F_o > 2\sigma(F_o)$; $wR2 = (\Sigma w(F_o^2 - F_c^2)^2/\Sigma(wF_c^2)^2)^{1/2}$ all reflections

$w=1/[\sigma^2(F_o^2)+(0.2000P)^2+300.0000P]$ where $P=(F_o^2+2F_c^2)/3$

Specific refinement details:

The crystals of [ReO_4⊂**1**]·7 NTf_2·MeCN were grown by diffusion of diethyl ether into an acetonitrile solution of [Zn_4L_4]·8NTf_2 containing excess of Bu_4NReO_4. The crystals immediately lost solvent after removal from the mother liquor and rapid handling prior to flash cooling in the cryostream was required to collect data. Despite these measures and the use of a high intensity laboratory source few reflections at greater than 1.05 Å resolution were observed. Nevertheless, the quality of the data is far more than sufficient to establish the connectivity of the structure. The asymmetric unit was found to contain one complete Zn_4L_4 assembly and associated counterions and solvent molecules. Bond lengths and angles within pairs of chemically identical organic ligands were restrained to be similar to each other and thermal parameter restraints (SIMU, RIGU) were applied to all atoms except for zinc and rhenium.

There is a significant amount of thermal motion in the extremities of the molecule, notably around the uncoordinated pyridyl groups; a number of restraints were required for the realistic modelling of these groups. Even with these restraints the thermal parameters of some pyridyl rings are larger than ideal resulting in high carbon, hydrogen and nitrogen U_{iso} min/max ratios. Modelling these groups as disordered over multiple discrete positions did not improve the overall model.

The anions within the structure show evidence of substantial disorder. All seven triflimide anions were modelled as disordered over two or three locations and multiple anions were modelled with partial occupancy. Some minor occupancy locations for the disordered anions could not be located in the electron density map and were therefore not modelled. Substantial bond length and thermal parameter restraints were applied to facilitate a reasonable refinement of the disordered anions most low occupancy disordered sites were modelled with isotropic thermal parameters.

Further reflecting the solvent loss and poor diffraction properties there is a significant amount of void volume in the lattice containing smeared electron density from disordered solvent. Consequently the SQUEEZE[10] function of PLATON[11] was employed to remove the contribution of the electron density associated with this highly disordered solvent, which gave a potential solvent accessible void of 3370 Å^3 per unit cell (a total of approximately 864 electrons). Since the diffuse solvent molecules could not be assigned conclusively to acetonitrile or diethyl ether only those solvent molecules that could be modelled with discrete atom positions are included in the formula.

[PF_6⊂1]·5.25PF_6·1.75NTf_2·7.5MeCN·0.5Et_2O [+ solvent]

Formula $C_{248.50}H_{183.50}F_{48}N_{45.25}O_{19.50}P_{6.25}S_{3.50}Zn_4$, *M* 5594.65, Triclinic, space group P -1 (#2), *a* 21.7175(13), *b* 35.407(2), *c* 36.087(2) Å, *a* 89.285(3), *b* 88.082(3), *g* 76.413(3)°, *V* 26957(3) Å^3, D_c 1.378 g cm^{-3}, *Z* 4, crystal size 0.220 by 0.170 by 0.090 mm, colour yellow, habit block, temperature 180(2) Kelvin, *l*(CuKa) 1.54178 Å, *m*(CuKa) 1.883 mm^{-1}, T(SADABS)$_{min,max}$ 0.5670, 0.7488, $2q_{max}$ 89.36, *hkl* range -19 19, -30 32, -32 32, *N* 110872, N_{ind} 41849(R_{merge} 0.0532), N_{obs} 29520(I > 2s(I)), N_{var} 6868, residuals* $R1(F)$ 0.1345, $wR2(F^2)$ 0.3854, GoF(all) 1.032, D$r_{min,max}$ -0.854, 1.327 e$^-$ Å^{-3}.

*$R1 = S||F_o| - |F_c||/S|F_o|$ for $F_o > 2s(F_o)$; $wR2 = (Sw(F_o^2 - F_c^2)^2/S(wF_c^2)^2)^{1/2}$ all reflections

$w=1/[s^2(F_o^2)+(0.1965P)^2+490.3014P]$ where $P=(F_o^2+2F_c^2)/3$

Specific refinement details:

The crystals of [PF_6⊂**1**]·5.25PF_6·1.75NTf_2·7.5MeCN·0.5Et_2O were grown by diffusion of diethyl ether into an acetonitrile solution of [Zn_4L_4]·8NTf_2 containing excess Bu_4NPF_6. The crystals immediately lost solvent after removal from the mother liquor and rapid handling prior to flash cooling in the cryostream

was required to collect data. Despite these measures and the use of a high intensity laboratory source few reflections at greater than 1.1 Å resolution were observed. Nevertheless, the quality of the data is far more than sufficient to establish the connectivity of the structure. The asymmetric unit was found to contain two complete Zn_4L_4 assemblies and associated counterions and solvent molecules. Bond lengths and angles within pairs of chemically identical organic ligands were restrained to be similar to each other and thermal parameter restraints (SIMU, RIGU) were applied to all atoms except for zinc.

One coordinated pyridine ring was modelled as disordered over two locations with bond length and thermal parameter restraints applied in order to obtain a reasonable refinement. In addition there is a significant amount of thermal motion in the extremities of the molecule, notably around the uncoordinated pyridyl rings; a number of restraints were required for the realistic modelling of these groups. Even with these restraints the thermal parameters of some pyridyl rings are larger than ideal resulting in high carbon, hydrogen and nitrogen U_{iso} min/max ratios. Modelling these groups as disordered over multiple discrete positions did not improve the overall model.

Several of the anions within the structure show evidence of disorder. Three hexafluorophosphate anions were modelled as disordered over two or three locations and multiple anions were modelled with partial occupancy. Some minor occupancy locations for the disordered anions could not be located in the electron density map and were therefore not modelled. Several solvent molecules were also modelled as disordered over multiple locations and/or with partial occupancy. Substantial bond length and thermal parameter restraints were applied to facilitate a reasonable refinement of the disordered hexafluorophosphate anions and solvent molecules and most low occupancy disordered groups were modelled with isotropic thermal parameters.

Further reflecting the solvent loss and poor diffraction properties there is a significant amount of void volume in the lattice containing smeared electron density from disordered solvent. Consequently the SQUEEZE[7] function of PLATON[8] was employed to remove the contribution of the electron density associated with this highly disordered solvent, which gave a potential solvent accessible void of 1948 $Å^3$ per unit cell (a total of approximately 606 electrons). Since the diffuse solvent molecules could not be assigned conclusively to acetonitrile or diethyl ether only those solvent molecules that could be modelled with discrete atom positions are included in the formula.

$[SbF_6\subset 1]\cdot 6.2SbF_6\cdot 0.8NTf_2$ [+ solvent]

Formula $C_{229.60}H_{156}F_{48}N_{36.80}O_{15.20}S_{1.60}Sb_{7.20}Zn_4$, *M* 5774.88, Monoclinic, space group C 2/c (#15), *a* 40.248(8), *b* 21.112(4), *c* 36.393(7) Å, β 116.69(3), *V* 27628(12) $Å^3$, D_c 1.388 g cm^{-3}, *Z* 4, crystal size 0.030 by 0.030 by 0.005 mm, colour yellow, habit plate, temperature 100(2) Kelvin, λ(Synchrotron) 0.6889 Å, μ(Synchrotron) 1.035 mm^{-1}, T(Analytical)$_{min,max}$ 0.987832257269, 1.0, $2\theta_{max}$ 39.47, *hkl* range -39 39, -20 20, -35 35, *N* 43587, N_{ind} 13573(R_{merge} 0.0212), N_{obs} 9672(I > 2σ(I)), N_{var} 1394, residuals* $R1(F)$ 0.1029, $wR2(F^2)$ 0.3125, GoF(all) 1.079, $\Delta\rho_{min,max}$

-0.429, 1.226 e^{-} Å^{-3}.

$^{*}R1 = \Sigma||F_o| - |F_c||/\Sigma|F_o|$ for $F_o > 2\sigma(F_o)$; $wR2 = (\Sigma w(F_o^2 - F_c^2)^2/\Sigma(wF_c^2)^2)^{1/2}$ all reflections

$w=1/[\sigma^2(F_o^2)+(0.2000P)^2+40.0000P]$ where $P=(F_o^2+2F_c^2)/3$

Specific refinement details:

The crystals of [SbF_6⊂**1**]·6.2SbF_6·0.8NTf_2 were grown by diffusion of diethyl ether into an acetonitrile solution of **1**·8NTf_2 containing excess $KSbF_6$. The crystals immediately lost solvent after removal from the mother liquor and rapid handling prior to flash cooling in the cryostream was required to collect data. Despite these measures and the use of synchrotron few reflections at greater than 1.02 Å resolution were observed. Nevertheless, the quality of the data is far more than sufficient to establish the connectivity of the structure. The asymmetric unit was found to contain one half of a Zn_4L_4 assembly and associated counterions. Bond lengths and angles within the two chemically identical organic ligands were restrained to be similar to each other and thermal parameter restraints (SIMU, RIGU) were applied to all atoms except for zinc and antimony.

Two non-coordinated pyridine rings were modelled as disordered over two locations with bond length and thermal parameter restraints applied in order to obtain a reasonable refinement. In addition there is a high level of thermal motion throughout the structure, most notably around the other non-coordinated pyridyl rings; a number of restraints were required for the realistic modelling of these groups. Even with these restraints, the thermal parameters of some pyridyl rings are larger than ideal. Modelling further pyridyl rings as disordered over multiple discrete positions did not improve the overall model.

The anions within the structure show evidence of disorder. One anion lattice site was modelled as a disordered mixture of hexafluoroantimonate and triflimide with refined occupancies of 0.6 and 0.4 respectively. Substantial bond length and thermal parameter restraints were applied to facilitate a reasonable refinement of the disordered anion and most low occupancy disordered groups were modelled with isotropic thermal parameters. The encapsulated hexafluoroantimonate, which is located on a special position, shows a high level of thermal motion; attempts were made to model this anion as disordered over multiple discrete positions but this did not improve the overall model.

Further reflecting the solvent loss and poor diffraction properties there is a substantial amount of void volume in the lattice containing smeared electron density from disordered solvent and five anions per Zn_4L_4 assembly (assigned to hexafluoroantimonate in the formula). Despite many attempts to model this region of disorder as a combination of solvent and anion molecules no reasonable fit could be found, even with the use of restraints or rigid body constraints. Consequently the SQUEEZE[10] function of PLATON[11] was employed to remove the contribution of the electron density associated with the highly disordered solvent and anions, which gave a potential solvent accessible void of 8741 Å^3 per unit cell (a total of approximately 3050 electrons). Since the diffuse solvent molecules could not be assigned conclusively to acetonitrile or diethyl ether they are not included in the formula.

[SbF_6⊂2]·7SbF_6·0.5MeCN [+ solvent]

Formula $C_{169}H_{121.50}F_{48}N_{24.50}O_{12}Sb_8Zn_4$, *M* 4834.88, Triclinic, space group P -1 (#2), *a* 26.410(5), *b* 28.142(6), *c* 30.240(6) Å, *α* 76.87(3), *β* 79.94(3), *γ* 85.85(3)°, *V* 21539(8) Å^3, D_c 1.491 g cm^{-3}, *Z* 4, crystal size 0.050 by 0.002 by 0.002 mm, colour colourless, habit needle, temperature 100(2) Kelvin, *λ*(Synchrotron) 0.6889 Å, *μ*(Synchrotron) 1.383 mm^{-1}, T(Analytical)$_{min,max}$ 0.986094687389, 1.0, $2\theta_{max}$ 30.73, *hkl* range -20 20, -21 21, -23 22, *N* 34101, N_{ind} 19553(R_{merge} 0.0867), N_{obs} 9693(I > 2σ(I)), N_{var} 4123, residuals* $R1(F)$ 0.1527, $wR2(F^2)$ 0.3875, GoF(all) 1.257, $\Delta\rho_{min,max}$ -0.682, 2.140 e$^-$ Å^{-3}.

*$R1 = \Sigma||F_o| - |F_c||/\Sigma|F_o|$ for $F_o > 2\sigma(F_o)$; $wR2 = (\Sigma w(F_o^2 - F_c^2)^2/\Sigma(wF_c^2)^2)^{1/2}$ all reflections

$w=1/[\sigma^2(F_o^2)+(0.2000P)^2]$ where $P=(F_o^2+2F_c^2)/3$

Specific refinement details:

The crystals of [SbF_6⊂**2**]·7SbF_6·0.5MeCN were grown by diffusion of diethyl ether into an acetonitrile solution of **2**·8NTf_2 containing excess $KSbF_6$. The crystals immediately lost solvent after removal from the mother liquor and rapid handling prior to flash cooling in the cryostream was required to collect data. The crystals were very small and were subject to rapid beam damage during data collection using synchrotron radiation. Consequently few reflections at greater than 1.3 Å resolution were observed and the quality of the integration is less than ideal. Due to the beam damage only 95% data completeness could be achieved. Nevertheless, the quality of the data is sufficient to establish the connectivity of the structure. The asymmetric unit was found to contain two complete of a Zn_4L_4 assemblies and associated counterions and solvent molecules.

In order to obtain a reasonable model for the organic parts of the structure the GRADE program[12] was employed using the GRADE Web Server[13] to generate a full set of bond distance and angle restraints (DFIX, DANG, FLAT). Due to the thermal motion and less than ideal resolution, thermal parameter restraints (SIMU, RIGU) were applied to all atoms except for iron and antimony to facilitate anisotropic refinement. Even with these restraints some thermal parameters remain larger than ideal as a consequence of the high level of thermal motion throughout the structure. Bond length and thermal parameter restraints were also applied to the hexafluoroantimonate anions.

Further reflecting the solvent loss and poor diffraction properties there is a substantial amount of void volume in the lattice containing smeared electron density from disordered solvent and 5.5 anions per Zn_4L_4 assembly (assigned to hexafluoroantimonate in the formula). Despite many attempts to model this region of disorder as a combination of solvent and anion molecules no reasonable fit could be found, even with the use of restraints or rigid body constraints. Consequently the SQUEEZE[10] function of PLATON[11] was employed to remove the contribution of the electron density associated with the

highly disordered solvent and anions, which gave a potential solvent accessible void of 7547 Å^3 per unit cell (a total of approximately 2533 electrons). Since the diffuse solvent molecule(s) could not be assigned conclusively to acetonitrile or diethyl ether only those solvent molecules that could be modelled with discrete atom positions are included in the formula.

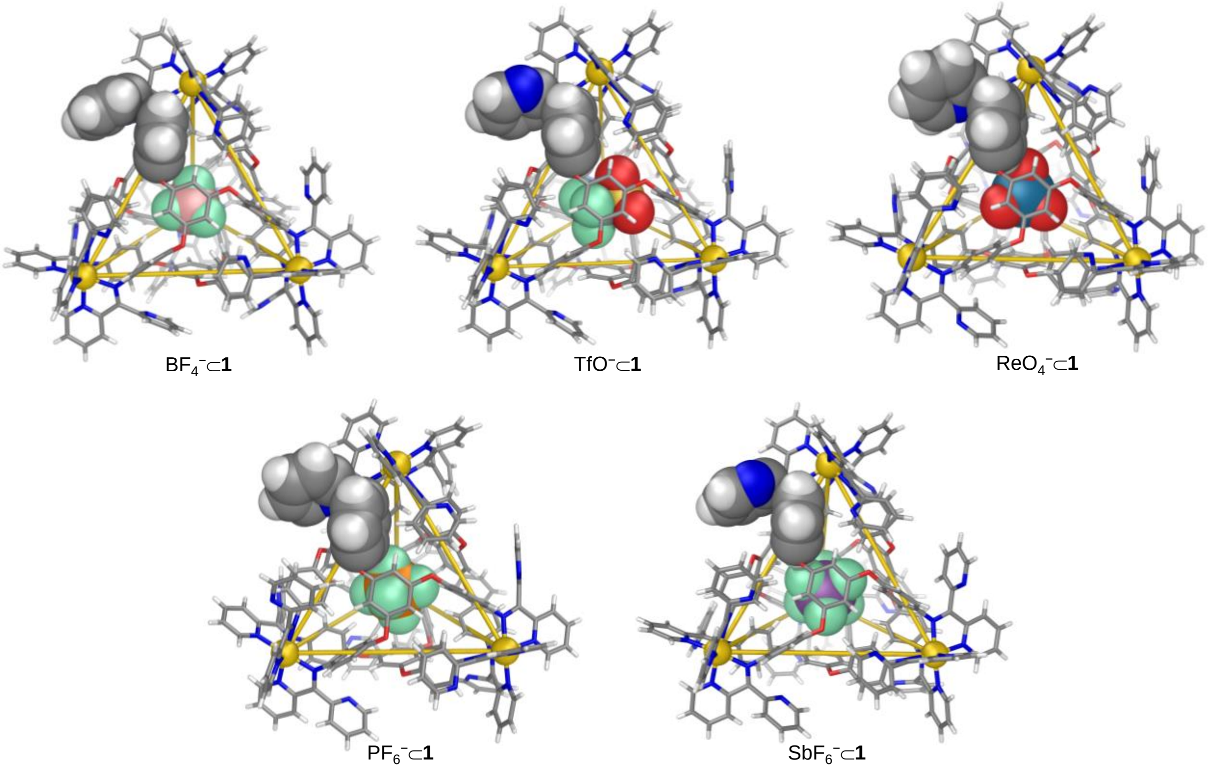

Figure S21. Crystal structures of $BF_4^-\subset\mathbf{1}$, $TfO^-\subset\mathbf{1}$, $ReO_4^-\subset\mathbf{1}$, $PF_6^-\subset\mathbf{1}$, and $SbF_6^-\subset\mathbf{1}$, with a representative free pyridine ring and its neighboring phenylene ring shown in space-filling mode in each case. Disorder, unbound counterions, and solvents of crystallization are omitted for clarity. We infer that the steric hindrance between the free pyridine rings and their neighboring phenylene rings restricts the free rotation of these phenylenes, consistent with the phenomenon observed in the ^{1}H NMR spectrum, which shows four distinct signals for these protons (Figure S1).

4. Volume calculations

In order to determine the available void space within **1** and **2**, VOIDOO calculations[14] based on the crystal structures (with the anionic guests removed) were performed. A virtual probe with a radius of 1.4 Å (set by default, water-sized) was employed, and the standard parameters tabulated below were used.[15] The results are shown in Table S1 and Figure S22.

Maximum number of volume-refinement cycles:	30
Minimum size of secondary grid:	3
Grid for plot files:	0.1
Primary grid spacing:	0.1
Plot grid spacing:	0.1

Table S1. Calculated volumes of cages **1** and **2** within their host-guest complexes.

Complex	Cavity volume (Å^3)
$BF_4^-\subset$**1**	134
$TfO^-\subset$**1**	148
$ReO_4^-\subset$**1**	139
$PF_6^-\subset$**1**	134, 139 (average 136)^a
$SbF_6^-\subset$**1**	143
$SbF_6^-\subset$**2**	145, 147 (average 146)^a

^{a} Structure contains two crystallographically independent cage molecules

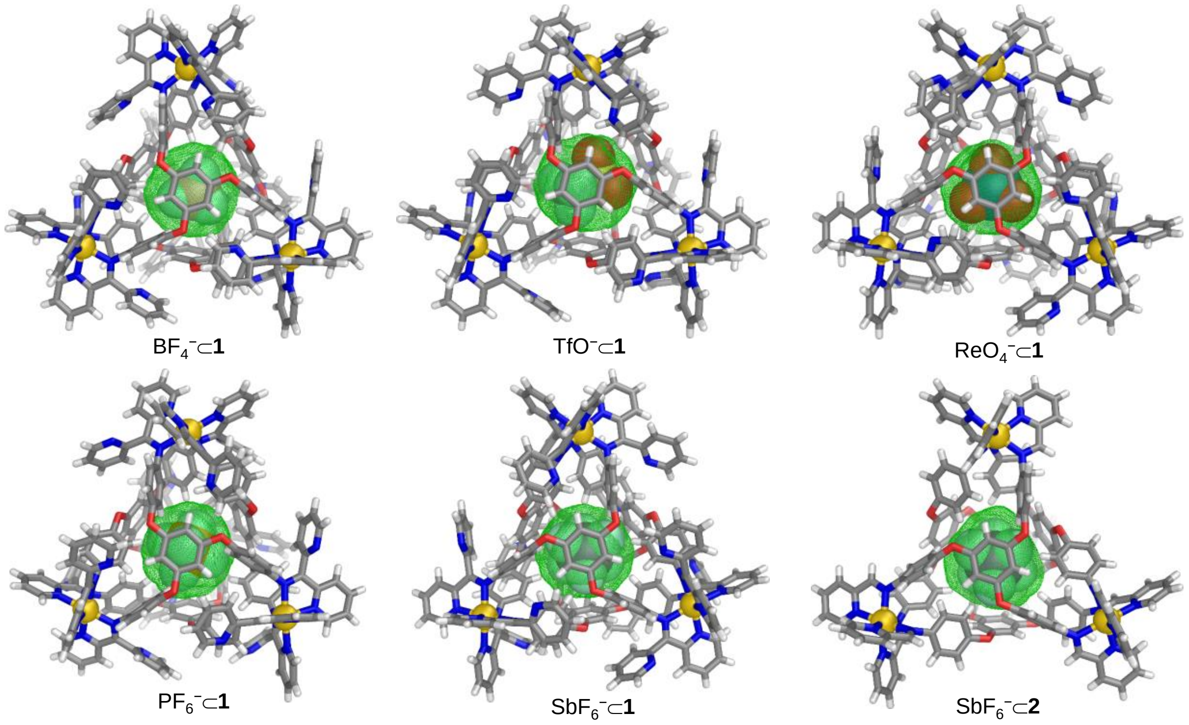

Figure S22. VOIDOO calculated void spaces (green mesh, overlaid with the encapsulated guests) within **1** and **2**. Only one of the two crystallographically independent cages is shown for $PF_6^-\subset$**1** and $SbF_6^-\subset$**2**.

5. Guest binding studies

A series of neutral molecules (Scheme S3), including cyclopentene, tetrahydrofuran, cyclohexane, tetrahydropyran, methylcyclopentane, 1,3-dioxane, methylcyclohexane, 1-methylcyclopentanol, cyclooctane, and 1,5-cyclooctadiene, and anions including TfO^- (TBAOTf, in which TBA represents tetrabutylammonium), PF_6^- ($TBAPF_6$), ReO_4^- ($TBAReO_4$), BF_4^- ($TBABF_4$), AsF_6^- ($KAsF_6$), and SbF_6^- ($KSbF_6$) were investigated as potential guests for the two cages. It was found that only the guests within the green box in Scheme S3 were encapsulated by cage **1**, showing slow-exchange binding on the NMR chemical shift time scale. Except BF_4^-, which was observed to bind within **1**, all other guests within the purple box in Scheme S3 could be bound by cage **2**, showing intermediate-exchange binding for PF_6^- and ReO_4^-, and slow-exchange binding for the rest.

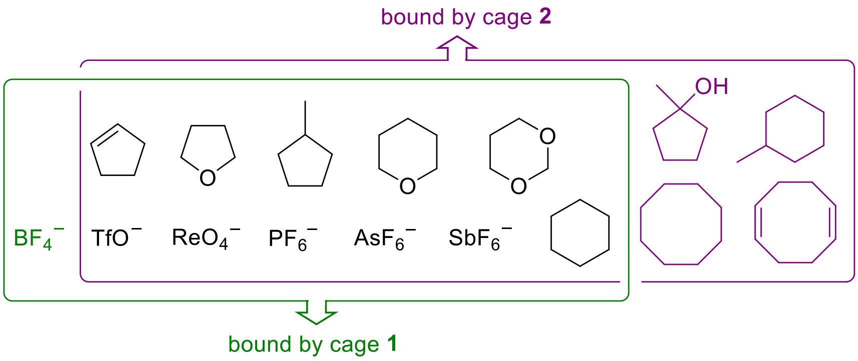

Scheme S3. Guests investigated. Guests in the green and purple boxes were bound within **1** and **2**, respectively.

^{1}H NMR titrations for neutral guests were carried out as follows: a stock solution of the cage was prepared at a concentration of 3 mM in CD_3CN. Different equivalents of guests were added into the NMR tube, which were allowed to stand at room temperature for 5 minutes before acquiring the NMR spectra. The binding constants were determined from the ^{1}H NMR titrations using $[HG]/[H]_0$ ($[HG]$ and $[H]_0$ represent the concentrations of host-guest complex at equilibrium and total host, respectively) as a function of the total concentration of guest added and fitting the data to the 1:1 binding model.[16] However for some particular guests, peak overlap between those of the host and host-guest complex prevented the accurate integration of peaks for each species and therefore the binding constants were not calculated, as detailed in Sections 5.1 and 5.2.

A cage concentration of 0.1 mM, calibrated by the 1,3,5-triethylbenzene internal standard, was used for anionic guests due to the higher binding constants that are too large to accurately measure at 3.0 mM, the concentration used for the neutral guests. Nevertheless only the binding constants of BF_4^- within **1** and TfO^- within **2** could be determined from direct titrations, while binding affinities for other anions were measured by competitive guest displacement based upon the binding constants for BF_4^- and TfO^-. Due to the slow kinetics of guest exchange, after addition of competitive guests to solutions containing preformed host-guest complexes, the mixtures were maintained at 50 °C for 12 h to reach equilibria prior to recording ^{1}H NMR spectra.

The binding constants for BF_4^- within **1** and TfO^- within **2** were determined as following:

$$K_a = \frac{[HG]}{[H]([G]_0 - [HG])}$$

Here the concentrations of the free host, [H], and the host-guest complex, [HG], at equilibrium after addition of a given amount of guest to the host solution could be determined from integrations on the ^{1}H NMR spectrum. As the concentration of the added guest, $[G]_0$, is known, the binding constant K_a can be quantified. Different equivalents of guest were added, and an average value was calculated.

The binding constants for other anions were assessed by competitive guest displacement as following:

$$[HG\mathbf{1}] + [G\mathbf{2}] \rightleftarrows [HG\mathbf{2}] + [G\mathbf{1}]$$

$$K_{rel} = \frac{K_a^2}{K_a^1} = \frac{[HG\mathbf{2}][G\mathbf{1}]}{[HG\mathbf{1}][G\mathbf{2}]} = \frac{[HG\mathbf{2}]([G\mathbf{1}]_0 - [HG\mathbf{1}])}{[HG\mathbf{1}]([G\mathbf{2}]_0 - [HG\mathbf{2}]}$$

Here the concentrations of host-guest complexes, [HG1] and [HG2], at equilibrium after addition of a given amount of competitive guest **G2** to a solution containing preformed host-guest complex **HG1** could be determined from integrations on the ^{1}H NMR spectrum. As the concentrations of the two added guests, $[G1]_0$ and $[G2]_0$, are known, the relative binding constant K_{rel} can be quantified. Different equivalents of competitive guests were added, and an average value of relative binding constant was calculated. As the binding constant for **G1** (K_a^1) has been predetermined, the binding constant for **G2** (K_a^2) can be calculated based upon the obtained K_{rel}.

The binding constants of cages **1** and **2** for the investigated neutral and anionic guests are tabulated in Table S2.

Table S2 Binding constants (K_a) of **1** and **2** for neutral and anionic guests measured at 298 K.

Guests	Cage **1**	Cage **2**
cyclohexane	5 ± 1 M^{-1}	(6.8 ± 0.2) × 10^2 M^{-1}
tetrahydropyran	8 ± 1 M^{-1}	(5.6 ± 0.5) × 10^2 M^{-1}
1,3-dioxane	15 ± 3 M^{-1}	(9.1 ± 0.7) × 10 M^{-1}
methylcyclopentane	4.6 ± 0.1 M^{-1}	(1.6 ± 0.1) × 10^2 M^{-1}
1-methylcyclopentanol	— [a]	10.5 ± 0.2 M^{-1}
methylcyclohexane	— [a]	— [b]
cyclooctane	— [a]	— [b]
1,5-cyclooctadiene	— [a]	— [b]
OTf^-	(3.3 ± 0.2) × 10^6 M^{-1}	(3.1 ± 0.1) × 10^4 M^{-1}
BF_4^-	(2.2 ± 0.3) × 10^3 M^{-1}	— [a]
PF_6^-	(1.7 ± 0.1) × 10^6 M^{-1}	— [c]
ReO_4^-	(1.3 ± 0.2) × 10^7 M^{-1}	— [c]
AsF_6^-	(2.6 ± 0.5) × 10^6 M^{-1}	(3.0 ± 0.1) × 10^4 M^{-1}
SbF_6^-	(9.3 ± 0.6) × 10^5 M^{-1}	(1.8 ± 0.1) × 10^5 M^{-1}

[a] No noticeable binding observed; [b] The binding was too weak to carry out ^{1}H NMR titrations for determination of the binding constant; [c] The binding constant could not be determined due to an intermediate-exchange binding process.

5.1 Guest binding properties of cage 1

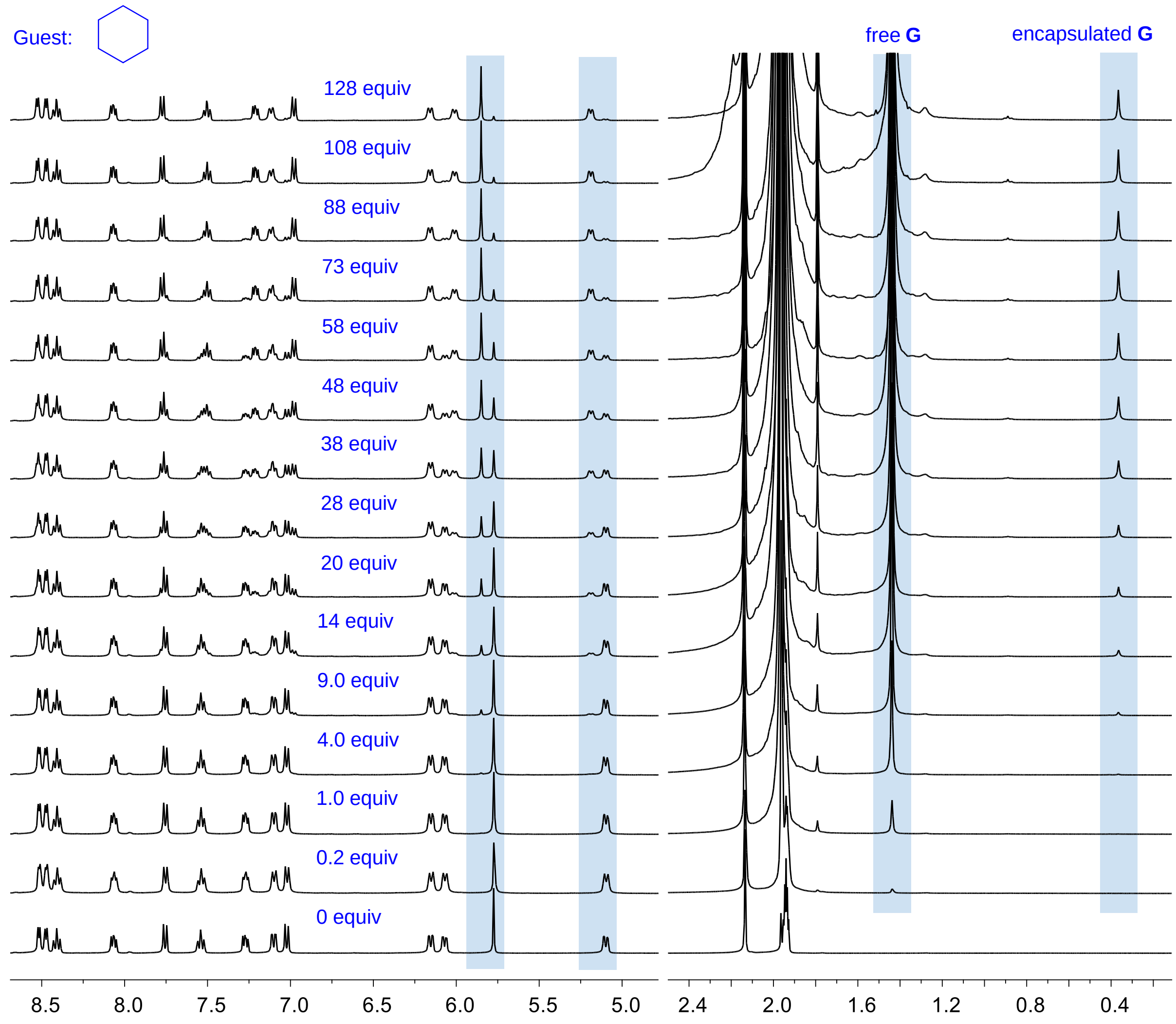

Figure S23. ^{1}H NMR (CD_3CN, 400 MHz, 298 K) titrations of the cyclohexane guest into a solution of **1** (3.0 mM).

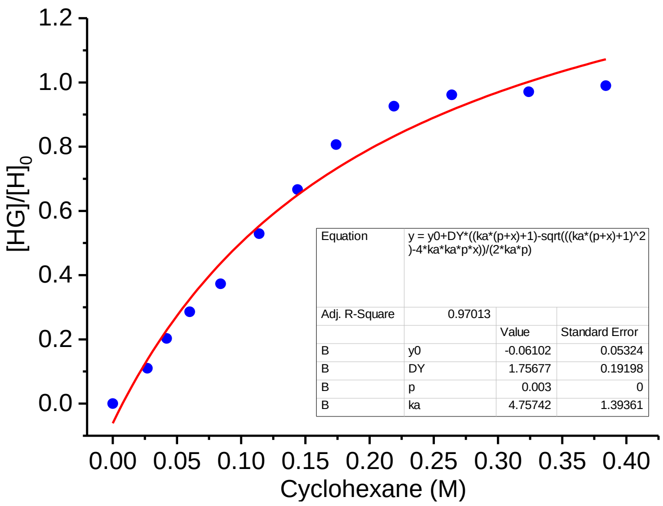

Figure S24. Degree of encapsulation, $[HG]/[H]_0$, as a function of the total concentration of cyclohexane and the corresponding fit to the 1:1 binding model, giving a binding constant of 5 ± 1 M^{-1} for cyclohexane⊂**1**.

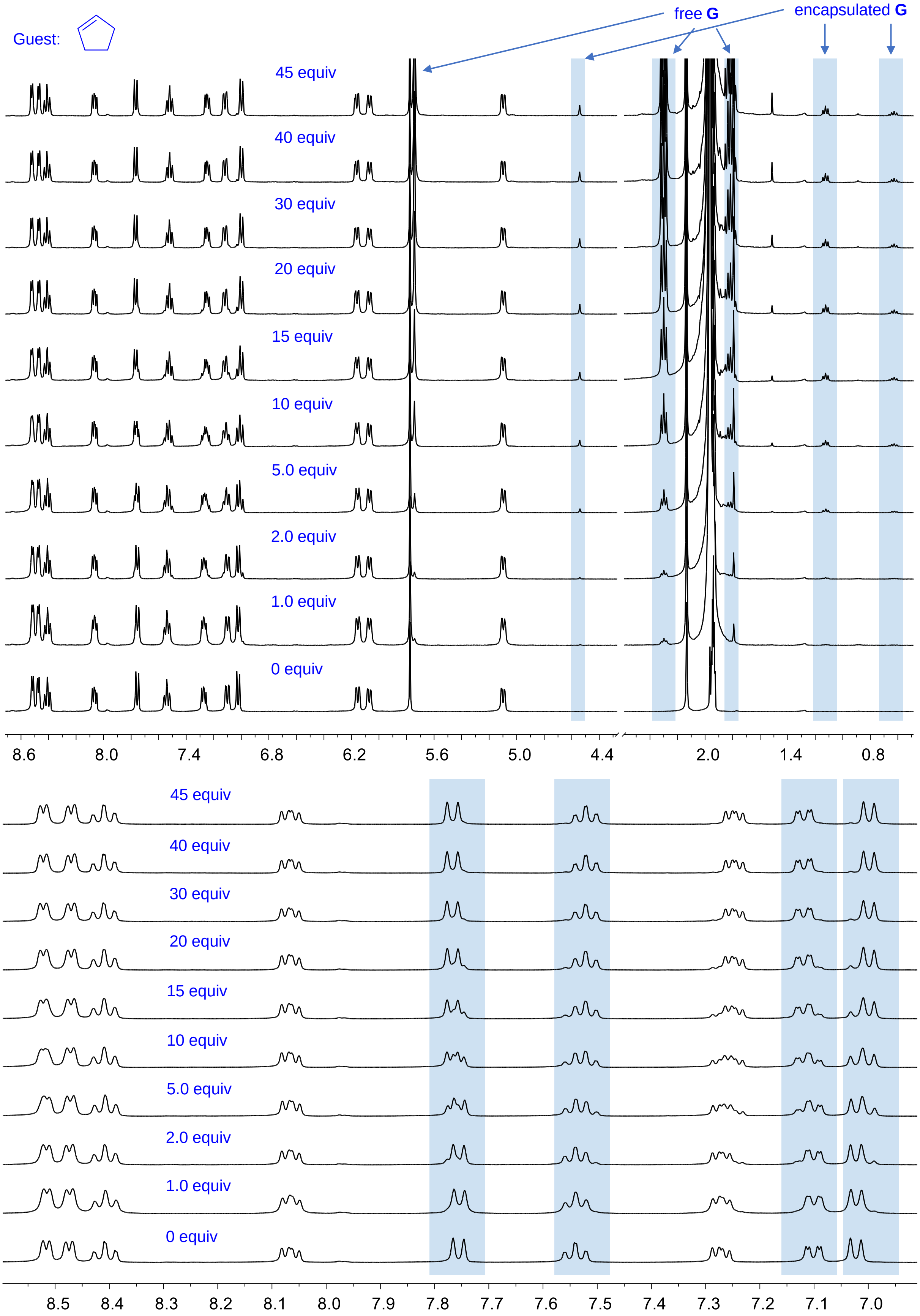

Figure S25. ^{1}H NMR (CD_3CN, 400 MHz, 298 K) titrations of the cyclopentene guest into a solution of **1** (3.0 mM). Peak overlap between those of the host and host-guest complex prevented accurate integration of the peaks for each species and therefore the determination of the binding constant.

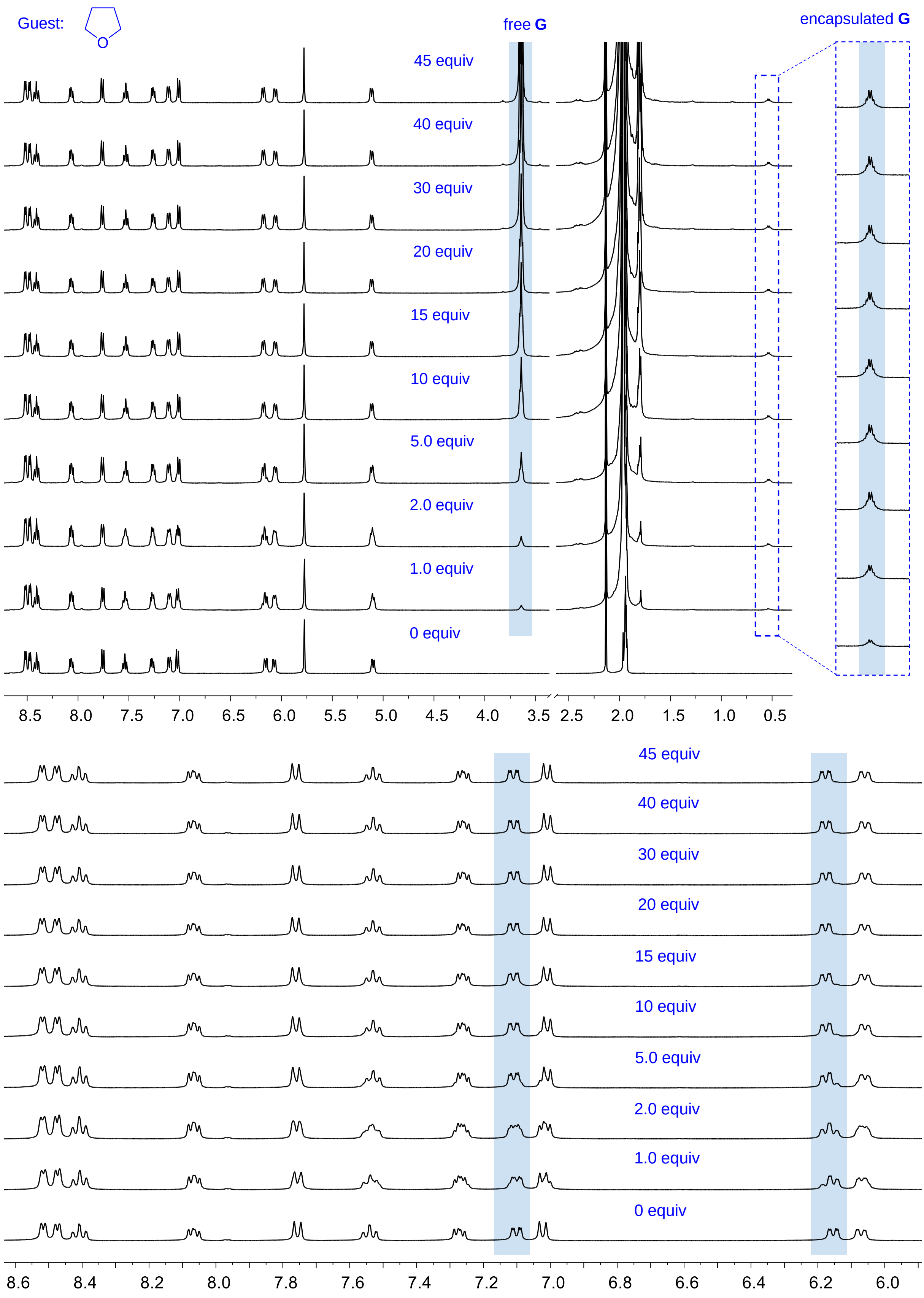

Figure S26. ^{1}H NMR (CD_3CN, 400 MHz, 298 K) titrations of the tetrahydrofuran guest into a solution of **1** (3.0 mM). Peak overlap between those of the host and host-guest complex prevented accurate integration of the peaks for each species and therefore the determination of the binding constant.

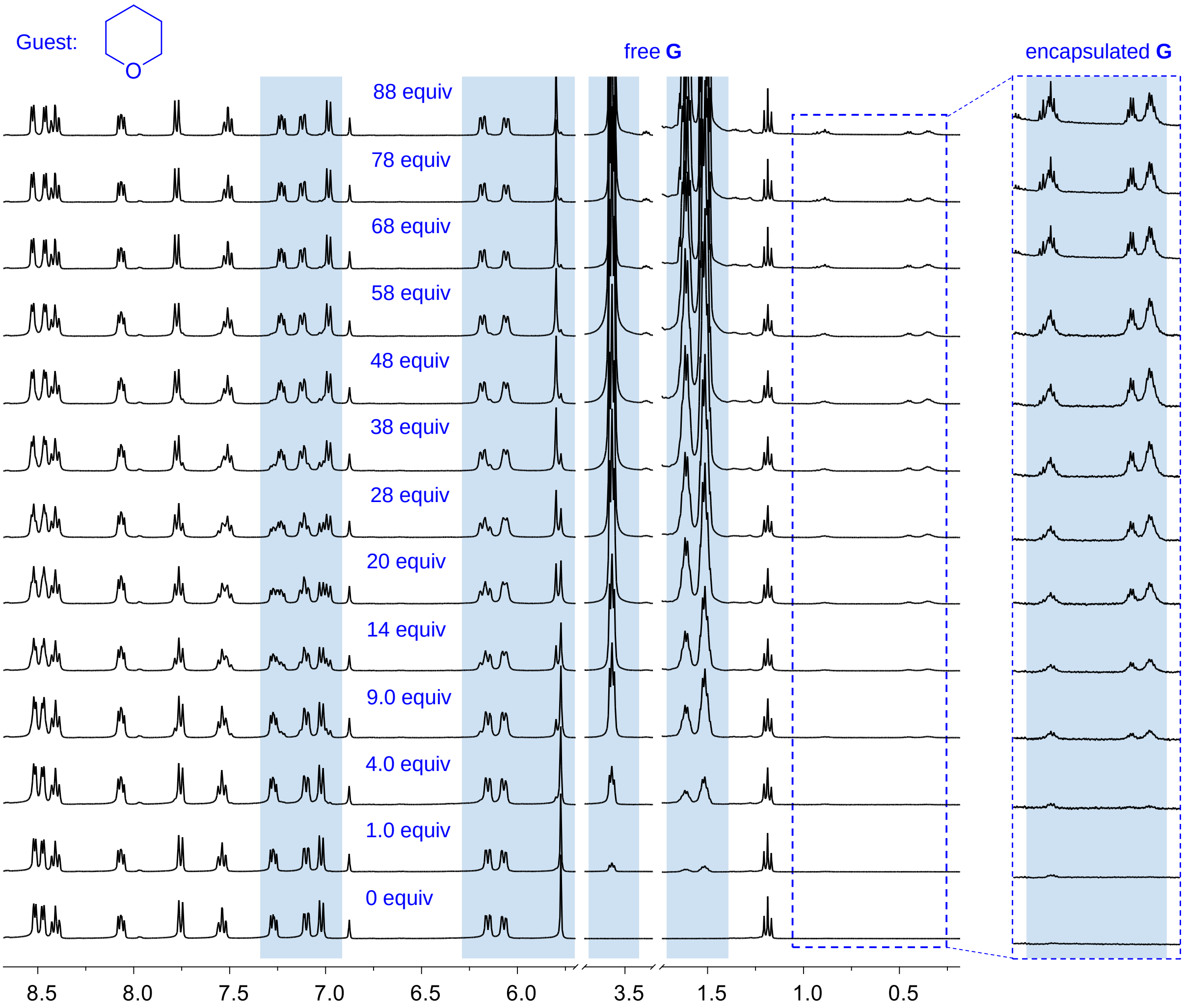

Figure S27. ^{1}H NMR (CD_3CN, 400 MHz, 298 K) titrations of the tetrahydropyran guest into a solution of **1** (3.0 mM).

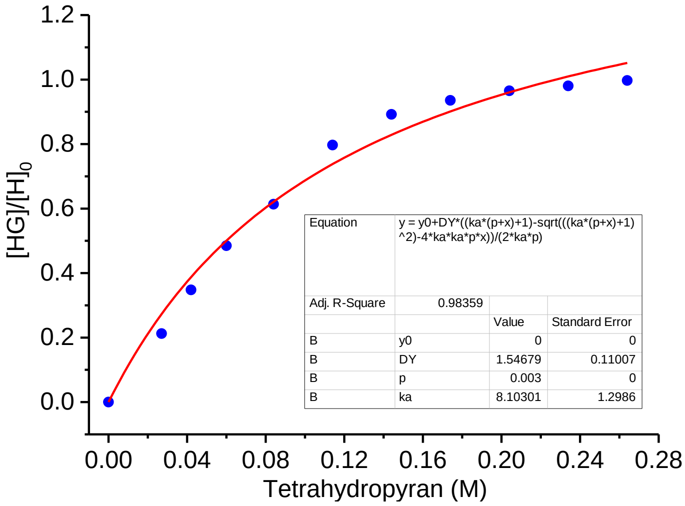

Figure S28. Degree of encapsulation, $[HG]/[H]_0$, as a function of the total concentration of tetrahydropyran present in solution and the corresponding fit of the data to the 1:1 binding model, giving a binding constant of 8 ± 1 M^{-1} for tetrahydropyran⊂**1**.

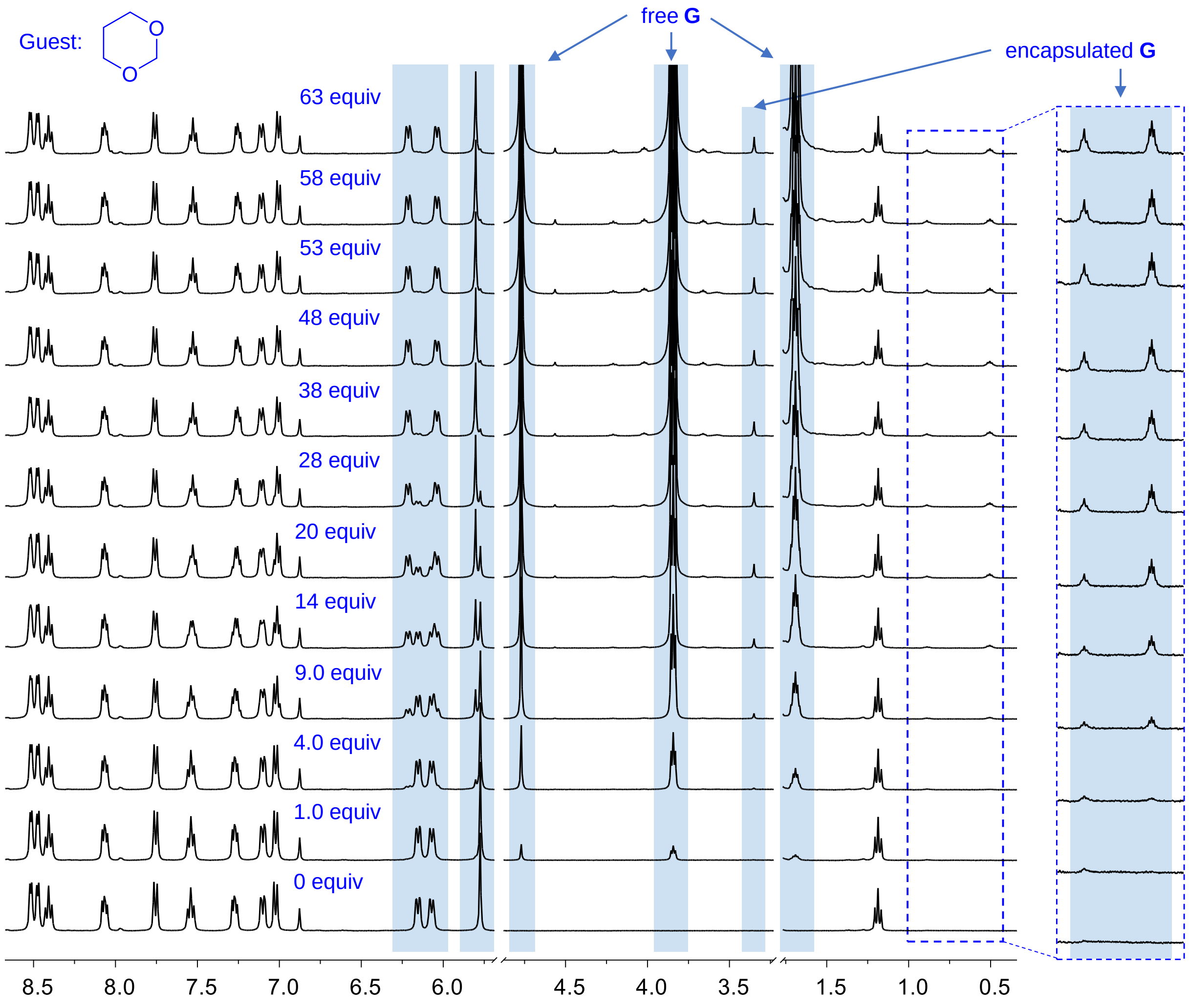

Figure S29. ^{1}H NMR (CD_3CN, 400 MHz, 298 K) titrations of the 1,3-dioxane guest into a solution of **1** (3.0 mM).

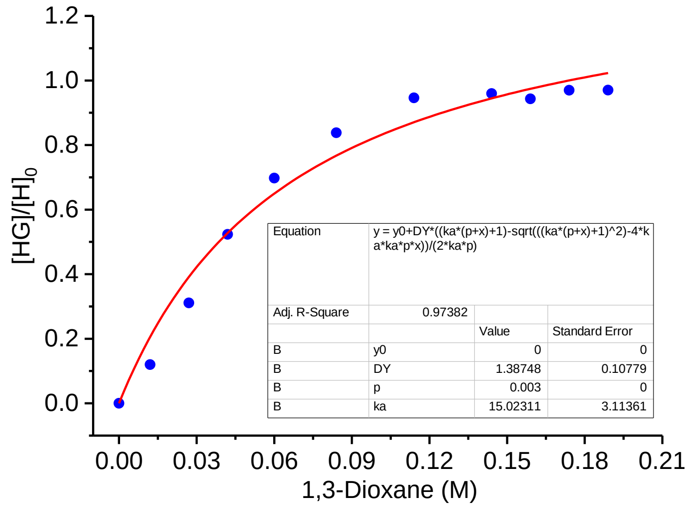

Figure S30. Degree of encapsulation, [HG]/[H]$_0$, as a function of the total concentration of 1,3-dioxane present in solution and the corresponding fit of the data to the 1:1 binding model, giving a binding constant of 15 ± 3 M^{-1} for 1,3-dioxane⊂**1**.

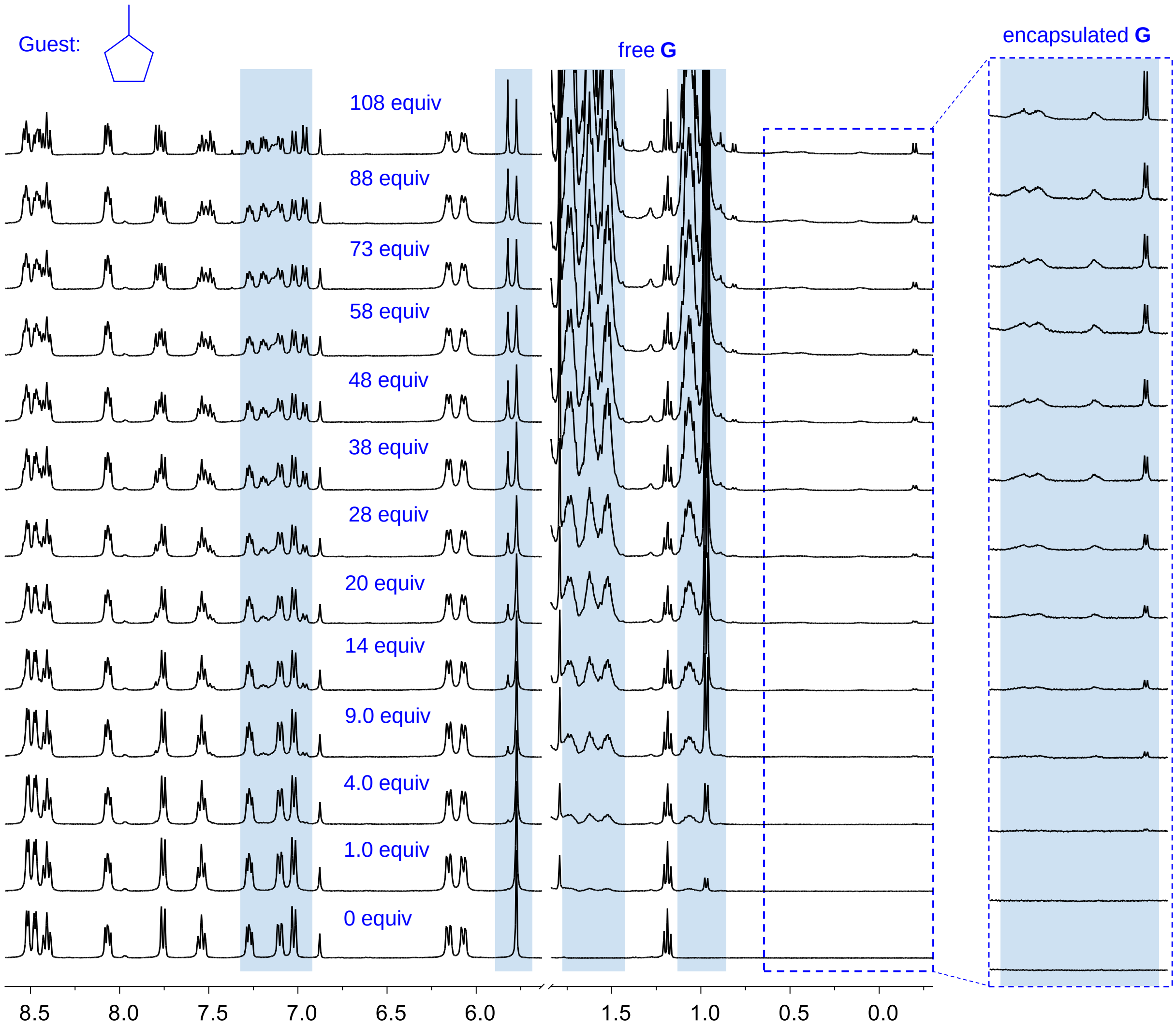

Figure S31. ^{1}H NMR (CD_3CN, 400 MHz, 298 K) titrations of the methylcyclopentane guest into a solution of **1** (3.0 mM).

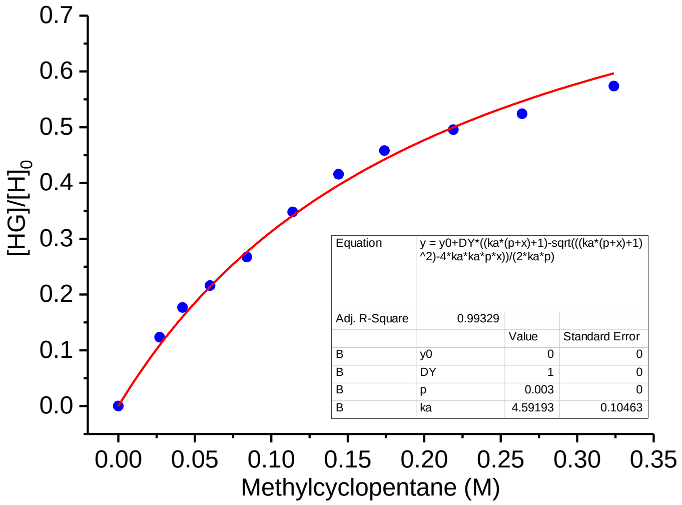

Figure S32. Degree of encapsulation, $[HG]/[H]_0$, as a function of the total concentration of methylcyclopentane present in solution and the corresponding fit of the data to the 1:1 binding model, giving a binding constant of 4.6 ± 0.1 M^{-1} for methylcyclopentane⊂**1**.

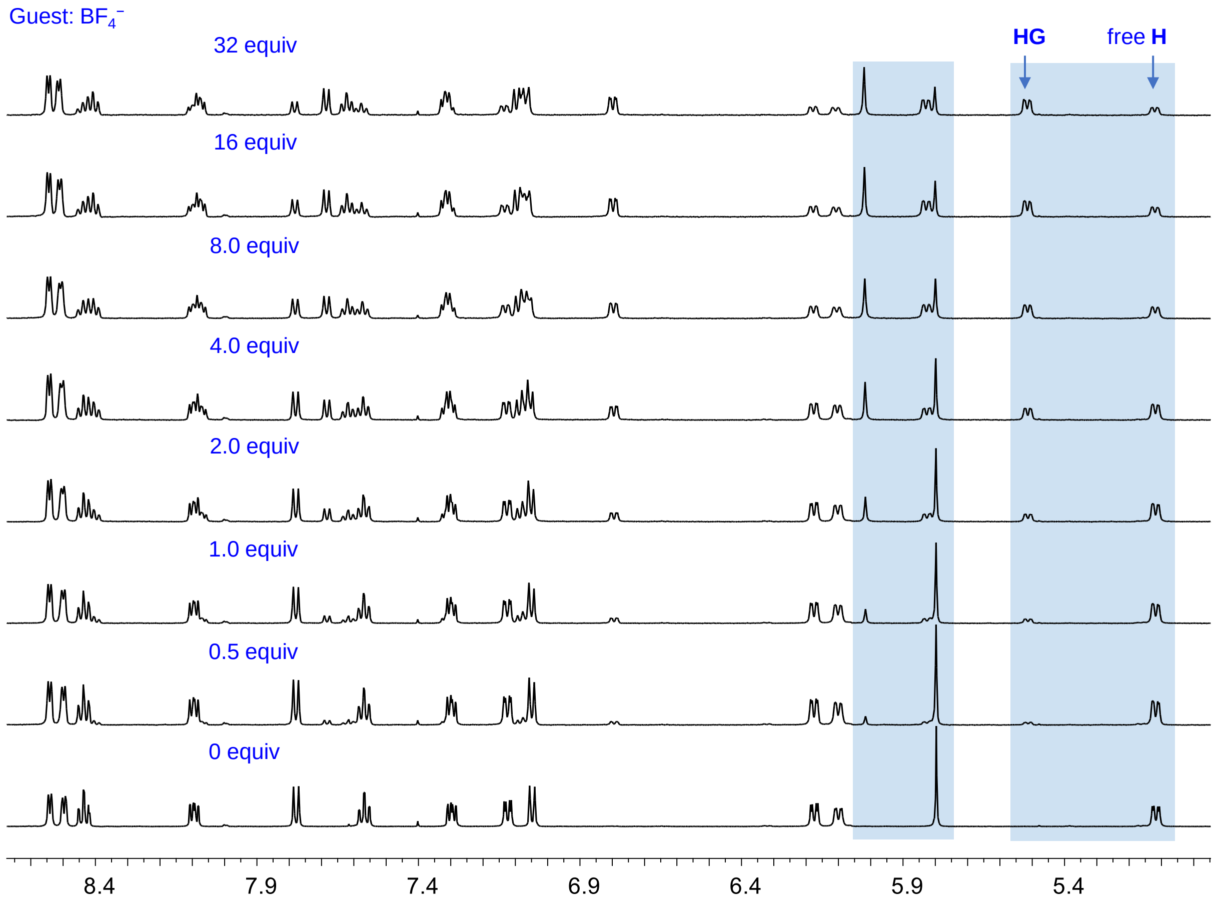

Figure S33. ^{1}H NMR (CD_3CN, 500 MHz, 298 K) titrations of BF_4^- into a solution of **1** (0.10 mM). The binding constant was calculated to be (2.2 ± 0.3) × 10^3 M^{-1} for BF_4^-⊂**1**.

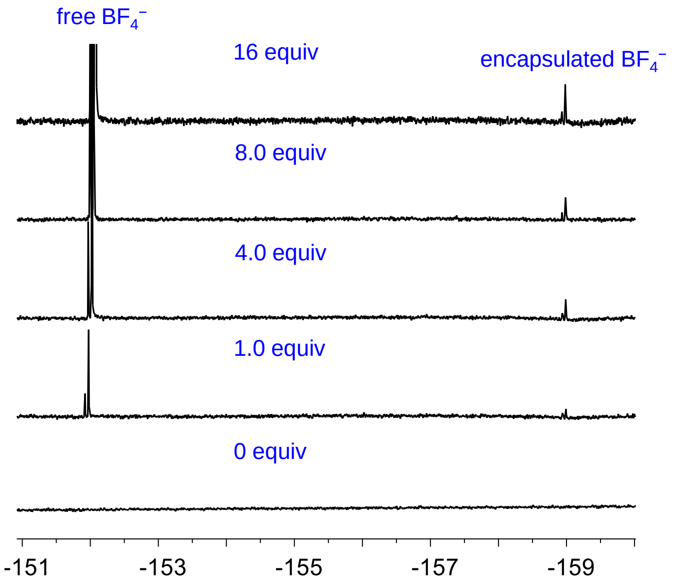

Figure S34. ^{19}F NMR (CD_3CN, 470.4 MHz, 298 K) titrations of BF_4^- into a solution of **1** (0.10 mM).

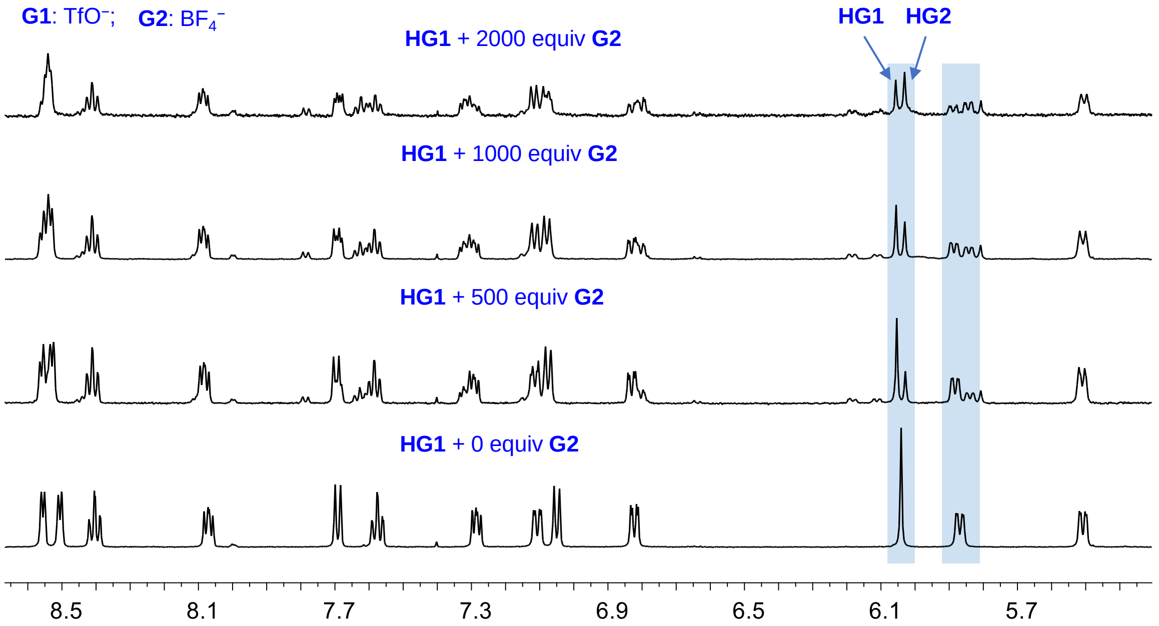

Figure S35. Competitive titrations of BF_4^- into a solution of $TfO^-\subset$**1** (0.10 mM), which was prepared by adding 1.5 equiv TfO^- to **1** in CD_3CN, monitored by ^1H NMR spectroscopy (CD_3CN, 500 MHz, 298K). The relative binding constant for TfO^- versus BF_4^- was estimated to be 1500. The binding constant of **1** for TfO^- was calculated to be $(3.3 \pm 0.2) \times 10^6$ M^{-1}.

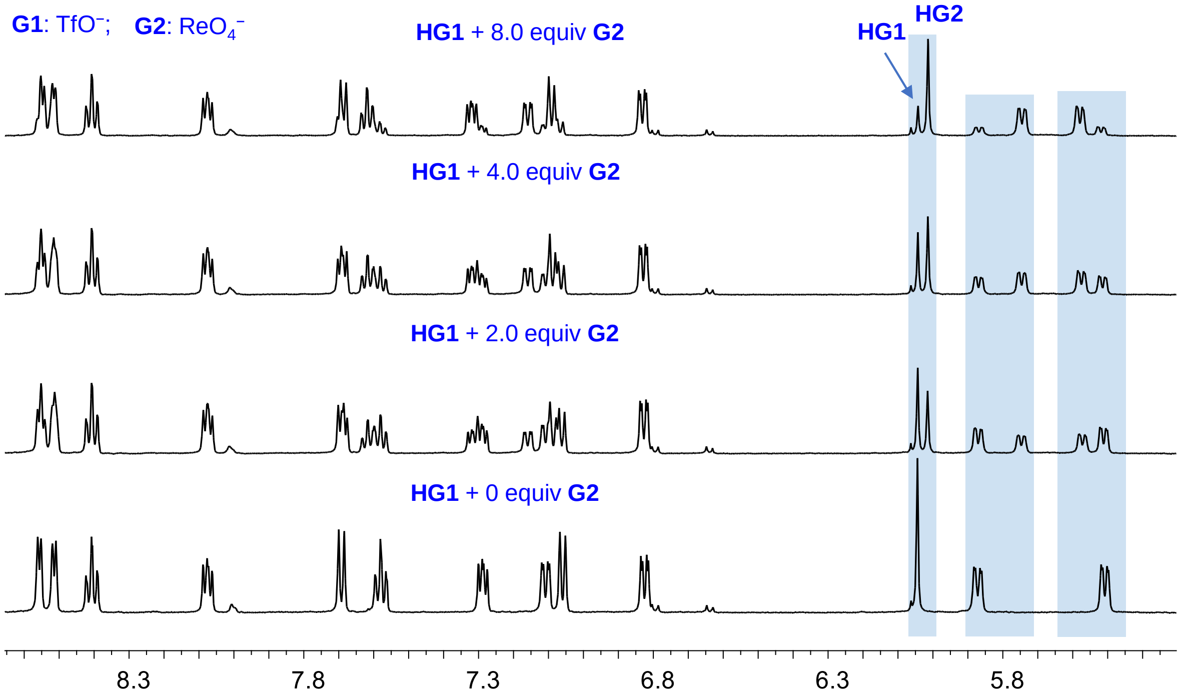

Figure S36. Competitive titrations of ReO_4^- into a solution of $TfO^-\subset$**1** (0.10 mM), which was prepared by adding 8.0 equiv TfO^- to **1** in CD_3CN, monitored by ^1H NMR spectroscopy (CD_3CN, 500 MHz, 298K). The relative binding constant for ReO_4^- versus TfO^- was estimated to be 3.8. The binding constant of **1** for ReO_4^- was calculated to be $(1.3 \pm 0.2) \times 10^7$ M^{-1}.

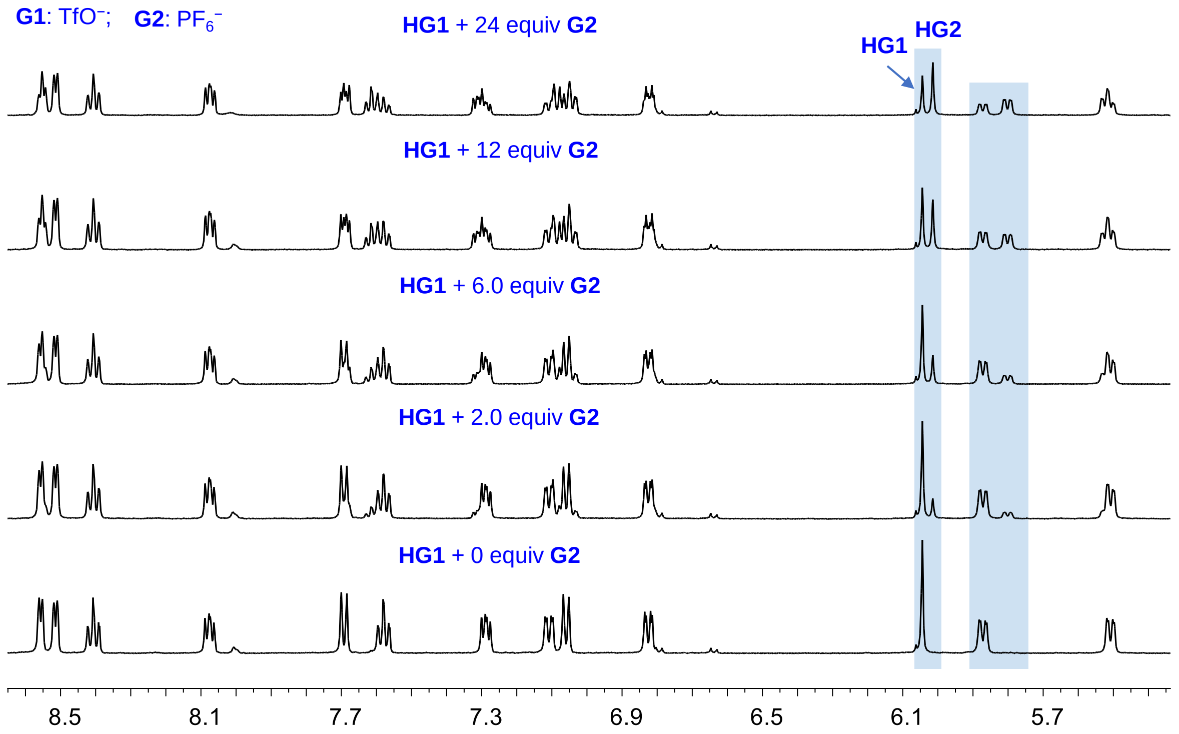

Figure S37. Competitive titrations of PF_6^- into a solution of $TfO^-\subset$**1** (0.10 mM), which was prepared by adding 8.0 equiv TfO^- to **1** in CD_3CN, monitored by ^1H NMR spectroscopy (CD_3CN, 500 MHz, 298K). The relative binding constant for PF_6^- versus TfO^- was estimated to be 0.50. The binding constant of **1** for PF_6^- was calculated to be $(1.7 \pm 0.1) \times 10^6$ M^{-1}.

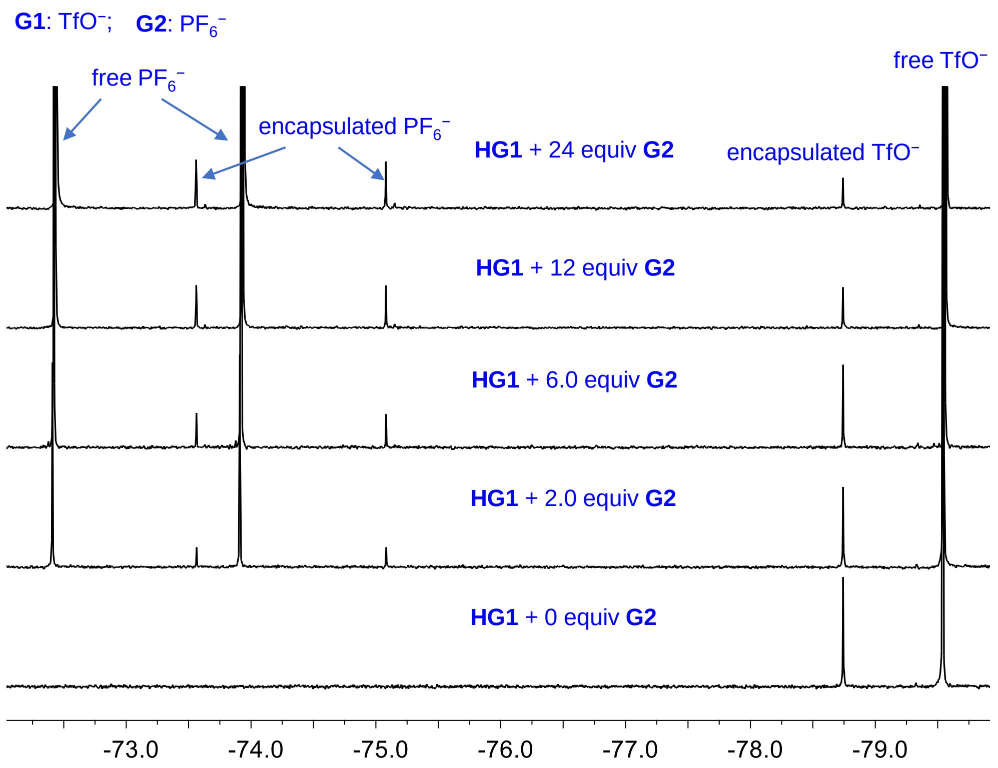

Figure S38. Competitive titrations of PF_6^- into a solution of $TfO^-\subset$**1** (0.10 mM), which was prepared by adding 8.0 equiv TfO^- to **1** in CD_3CN, monitored by ^{19}F NMR spectroscopy (CD_3CN, 470.4 MHz, 298 K).

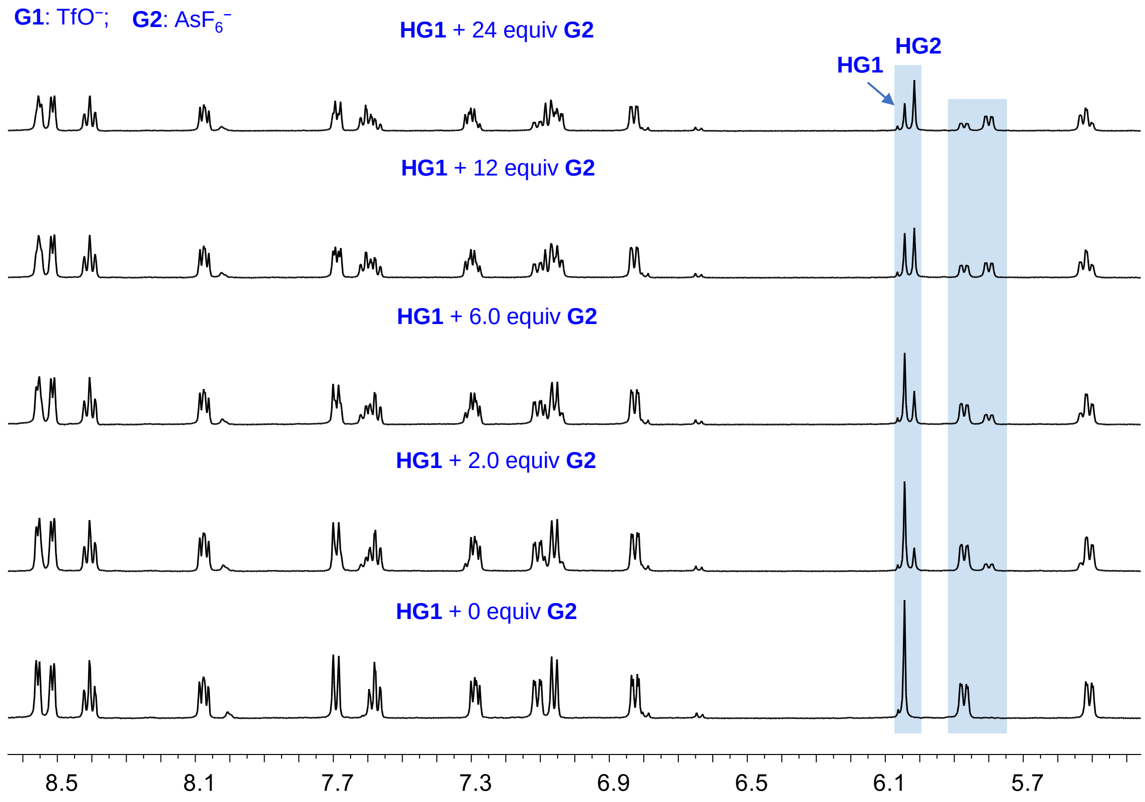

Figure S39. Competitive titrations of AsF_6^- into a solution of TfO^-⊂**1** (0.10 mM), which was prepared by adding 8.0 equiv TfO^- to **1** in CD_3CN, monitored by ^1H NMR spectroscopy (CD_3CN, 500 MHz, 298K). The relative binding constant for AsF_6^- versus TfO^- was estimated to be 0.78. The binding constant of **1** for AsF_6^- was calculated to be $(2.6 \pm 0.5) \times 10^6$ M^{-1}.

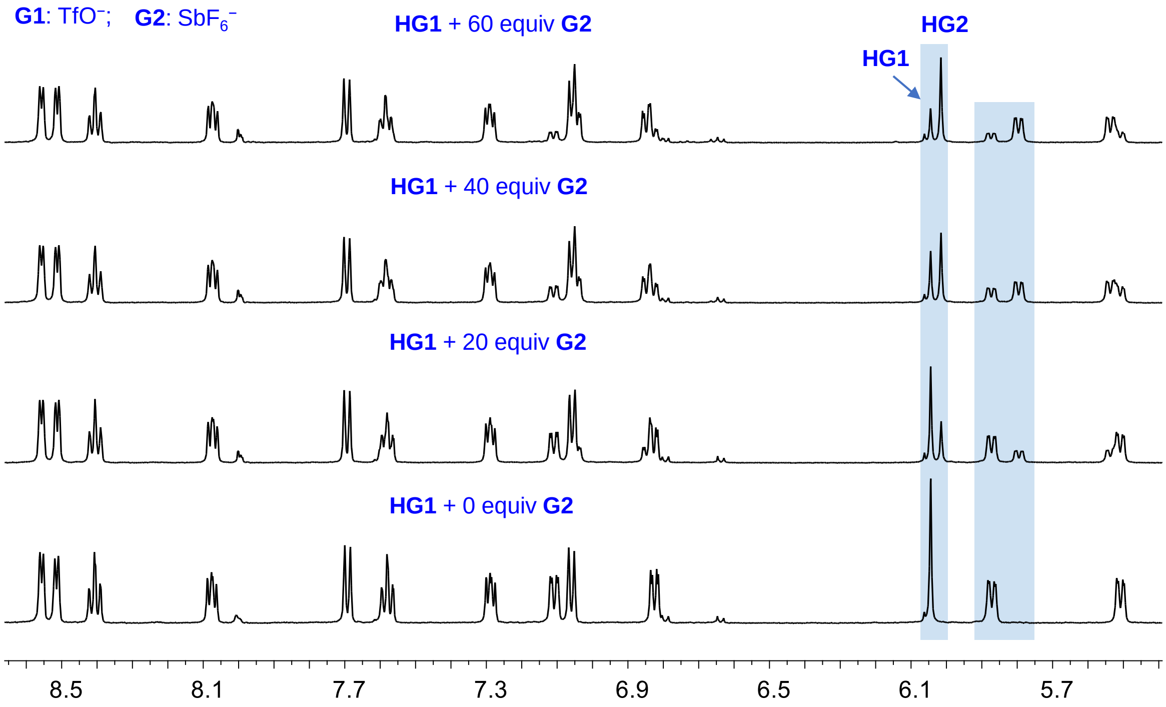

Figure S40. Competitive titrations of SbF_6^- into a solution of TfO^-⊂**1** (0.10 mM), which was prepared by adding 8.0 equiv TfO^- to **1** in CD_3CN, monitored by ^1H NMR spectroscopy (CD_3CN, 500 MHz, 298K). The relative binding constant for SbF_6^- versus TfO^- was estimated to be 0.28. The binding constant of **1** for SbF_6^- was calculated to be $(9.3 \pm 0.6) \times 10^5$ M^{-1}.

5.2 Guest binding properties of cage 2

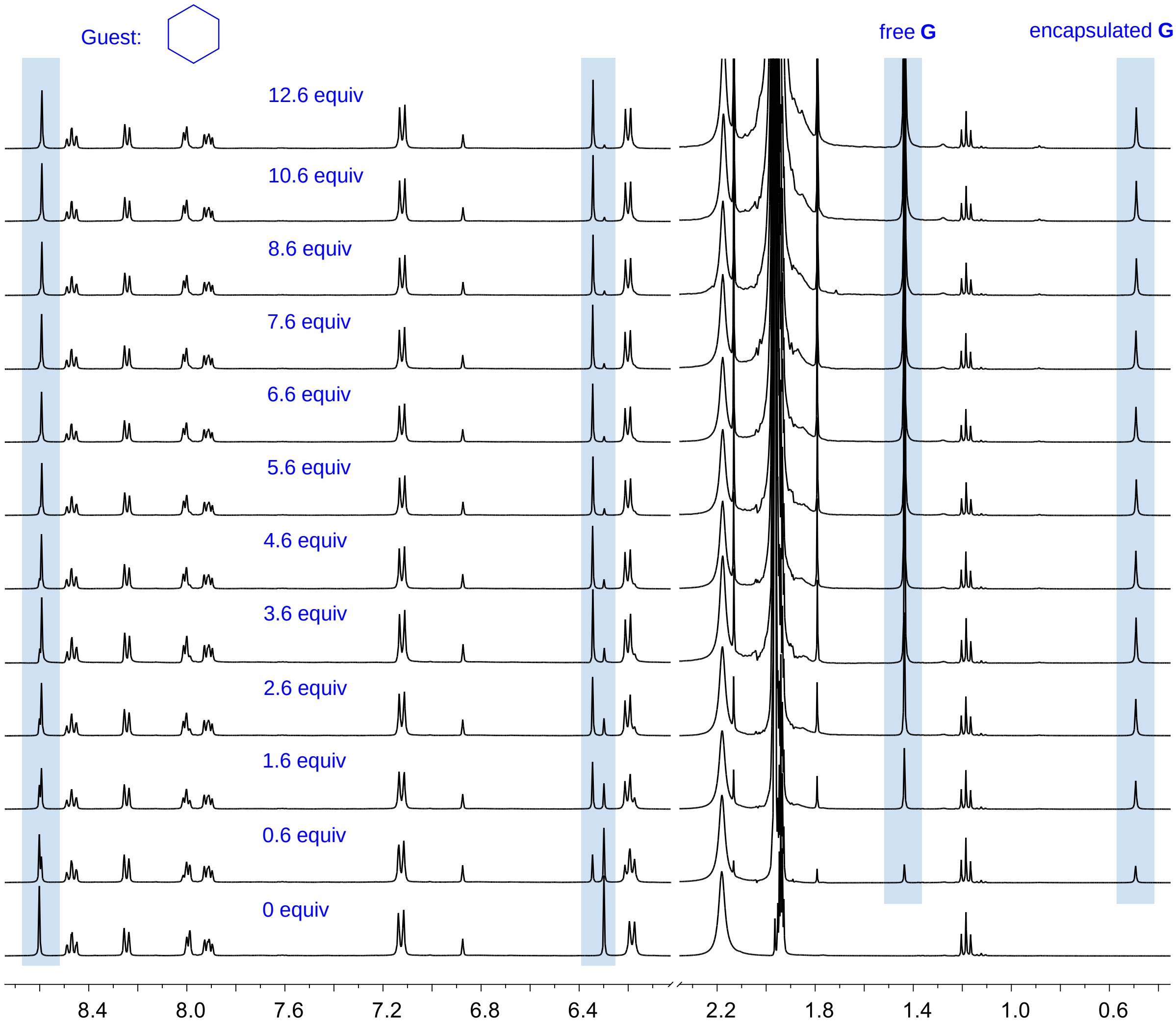

Figure S41. ^{1}H NMR (CD_3CN, 400 MHz, 298 K) titrations of the cyclohexane guest into a solution of **2** (3.0 mM).

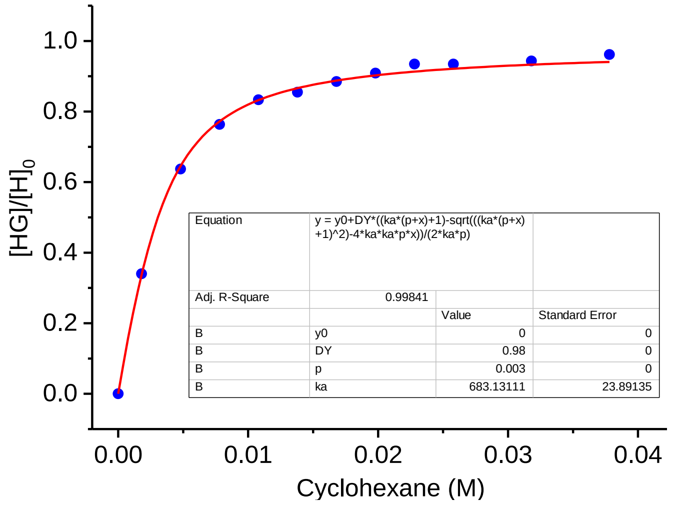

Figure S42. Degree of encapsulation, $[HG]/[H]_0$, as a function of the total concentration of cyclohexane and the corresponding fit to the 1:1 binding model, giving a binding constant of (6.8 ± 0.2) × 10^{2} M^{-1} for cyclohexane⊂**2**.

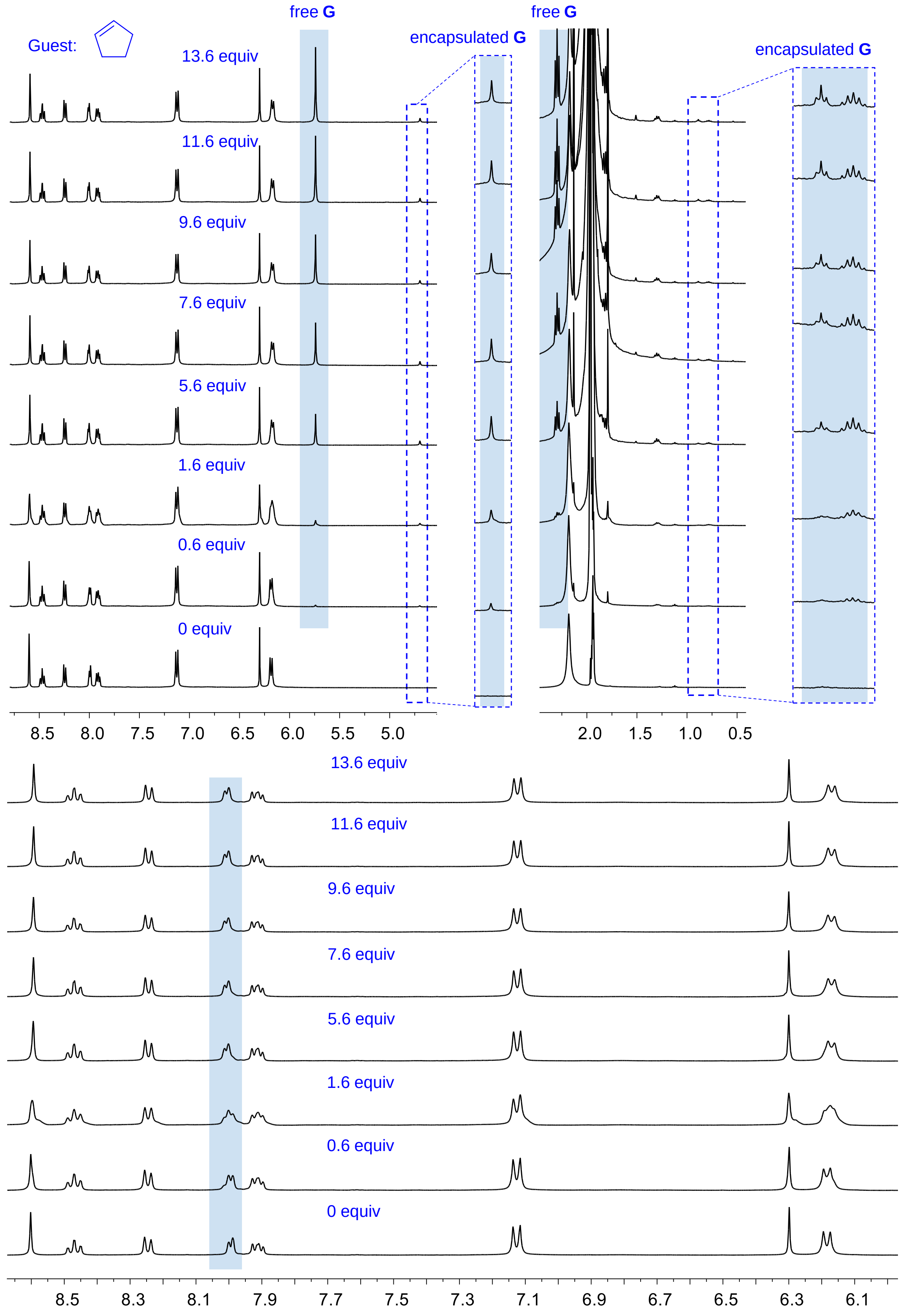

Figure S43. ^{1}H NMR (CD_3CN, 400 MHz, 298 K) titrations of the cyclopentene guest into a solution of **2** (3.0 mM). Peak overlap between those of the host and host-guest complex prevented accurate integration of the peaks for each species and therefore the determination of the binding constant.

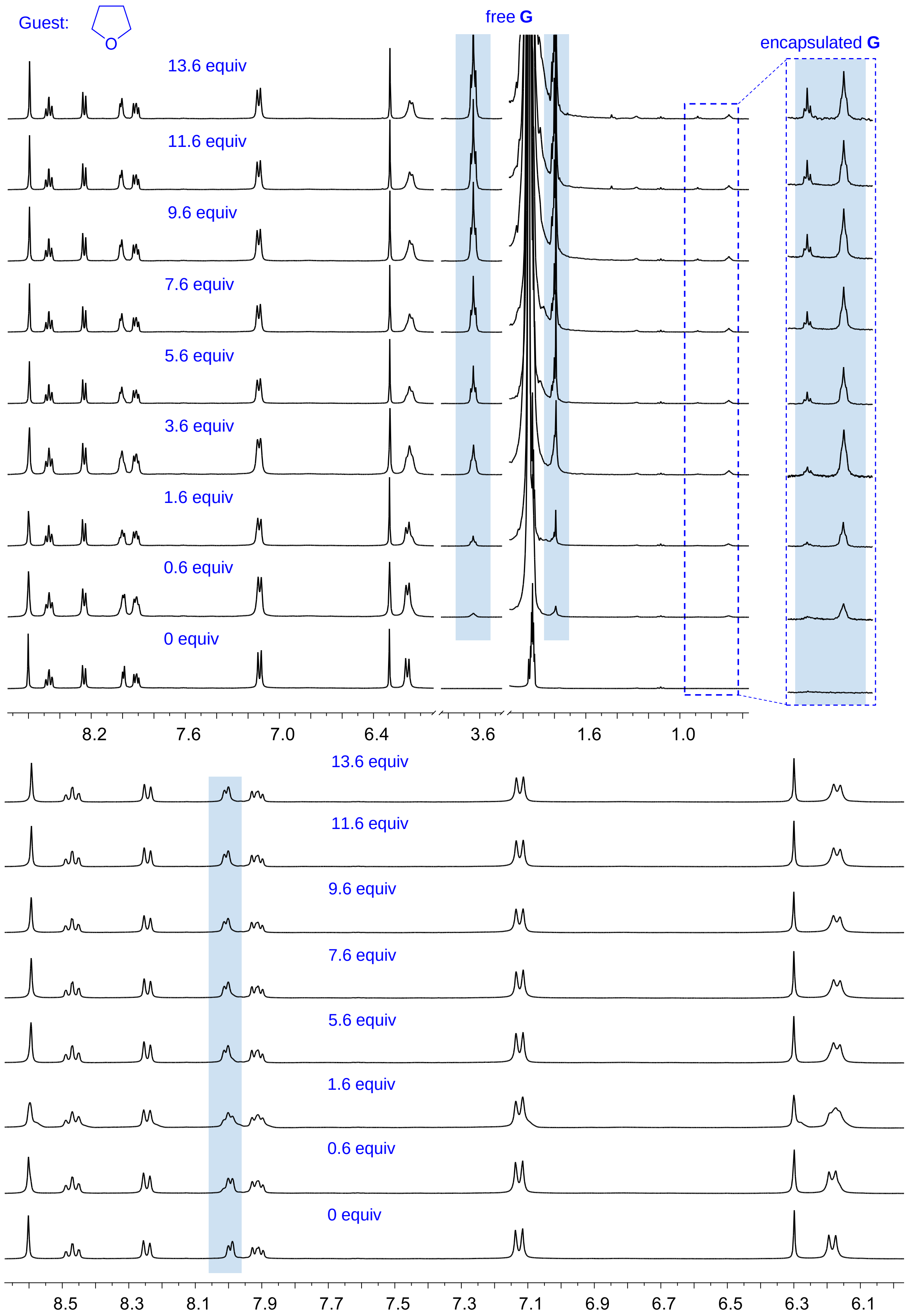

Figure S44. ^{1}H NMR (CD_3CN, 400 MHz, 298 K) titrations of the tetrahydrofuran guest into a solution of **2** (3.0 mM). Peak overlap between those of the host and host-guest complex prevented accurate integration of the peaks for each species and therefore the determination of the binding constant.

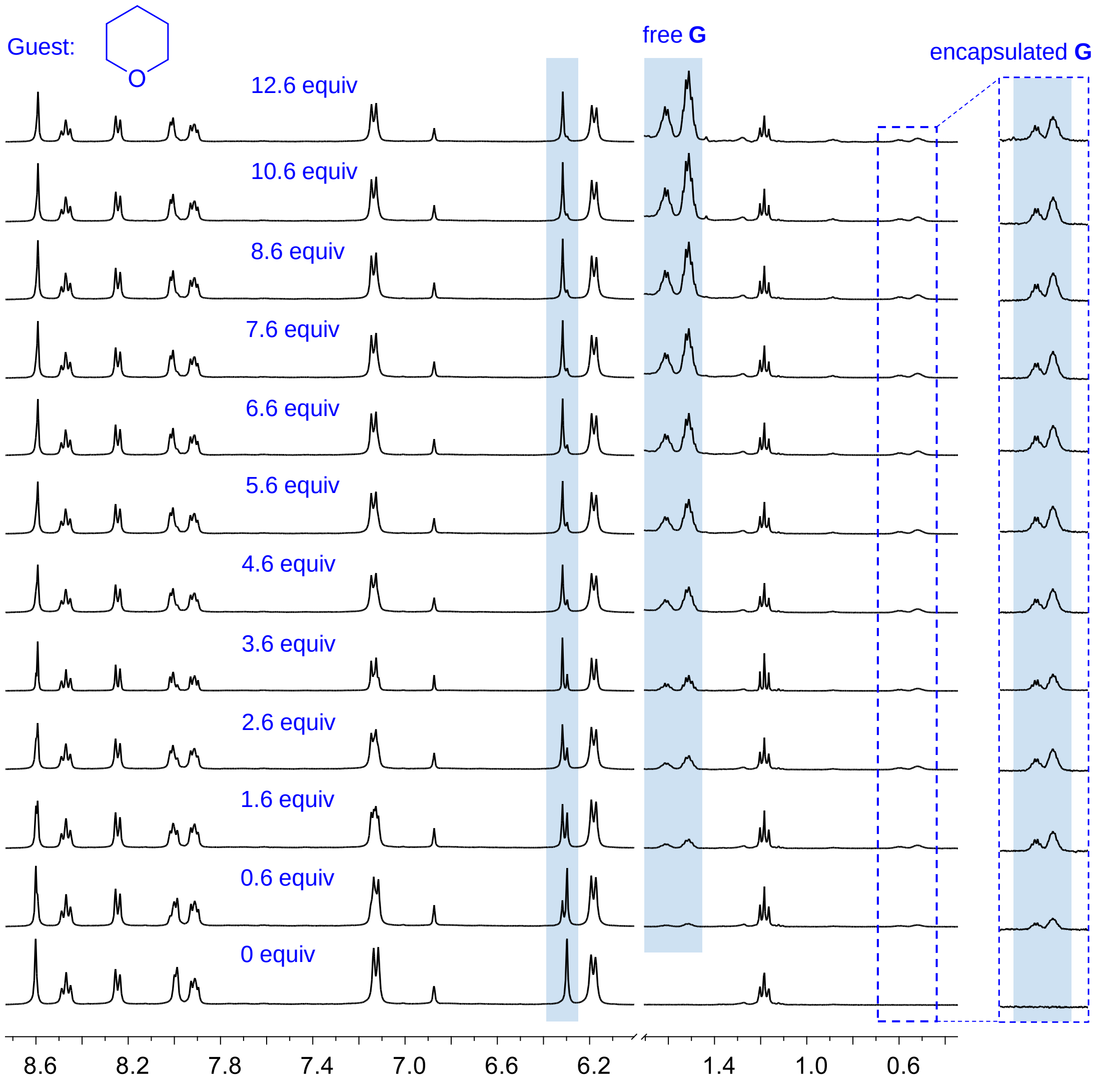

Figure S45. ^{1}H NMR (CD_3CN, 400 MHz, 298 K) titrations of the tetrahydropyran guest into a solution of **2** (3.0 mM).

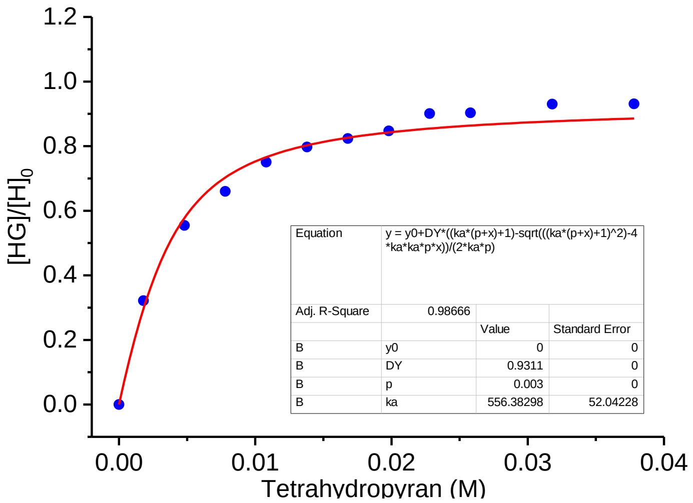

Figure S46. Degree of encapsulation, $[HG]/[H]_0$, as a function of the total concentration of tetrahydropyran present in solution and the corresponding fit of the data to the 1:1 binding model, giving a binding constant of $(5.6 \pm 0.5) \times 10^2$ M^{-1} for tetrahydropyran⊂**2**.

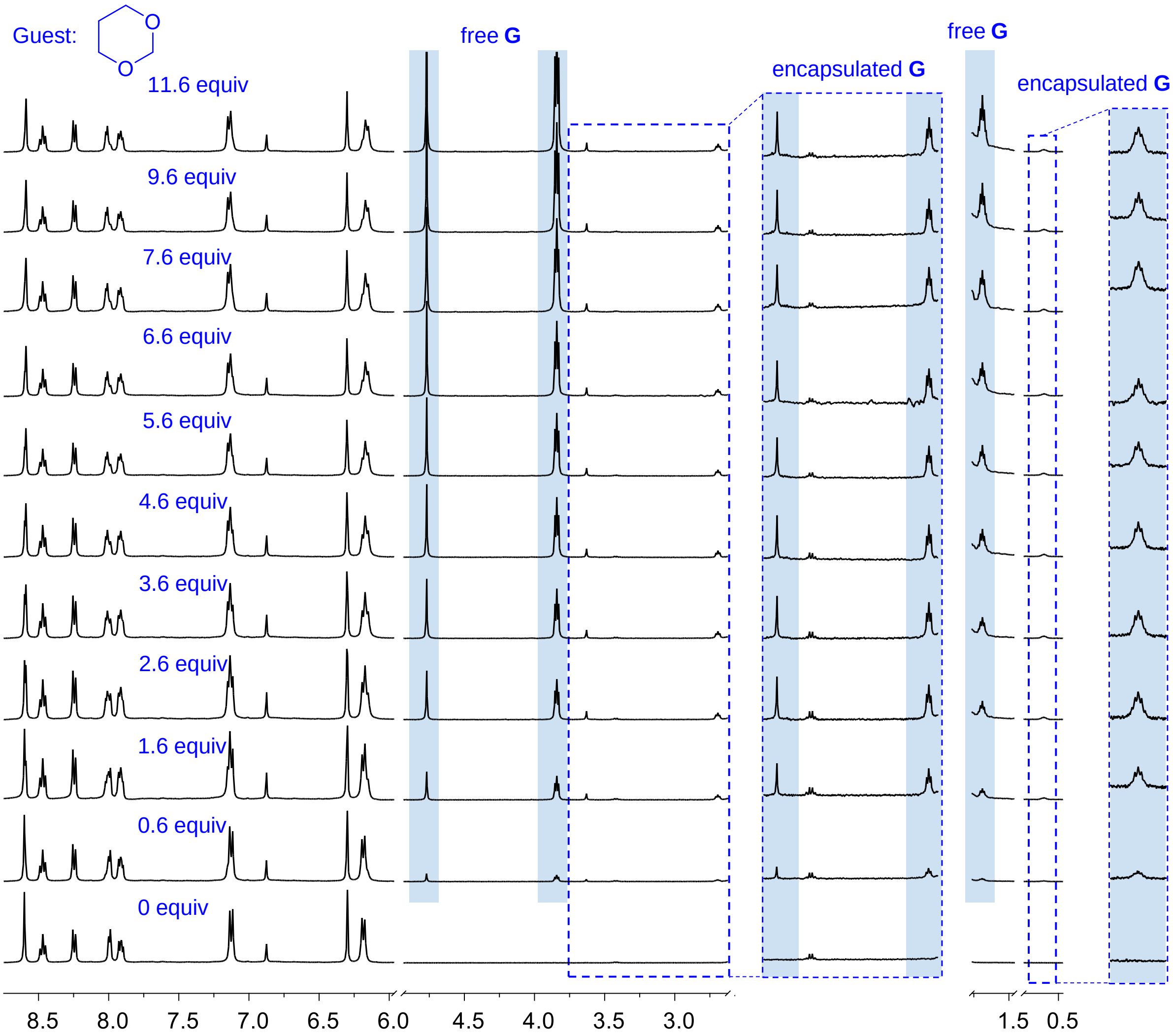

Figure S47. ^{1}H NMR (CD_3CN, 400 MHz, 298 K) titrations of the 1,3-dioxane guest into a solution of **2** (3.0 mM).

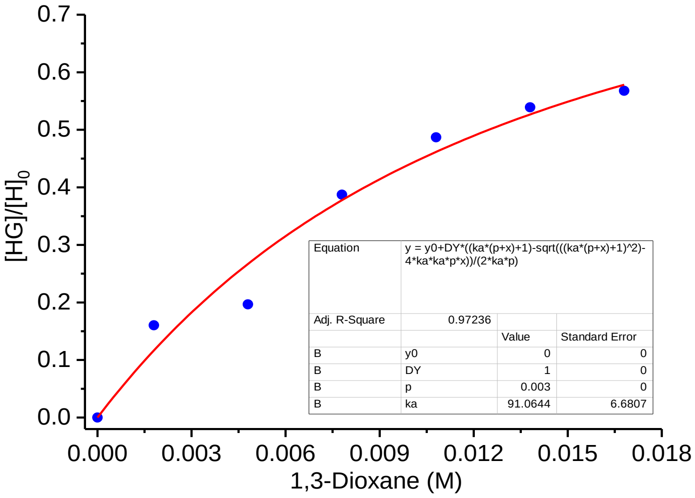

Figure S48. Degree of encapsulation, $[HG]/[H]_0$, as a function of the total concentration of 1,3-dioxane present in solution and the corresponding fit of the data to the 1:1 binding model, giving a binding constant of $(9.1 \pm 0.7) \times 10$ M^{-1} for 1,3-dioxane⊂**2**.

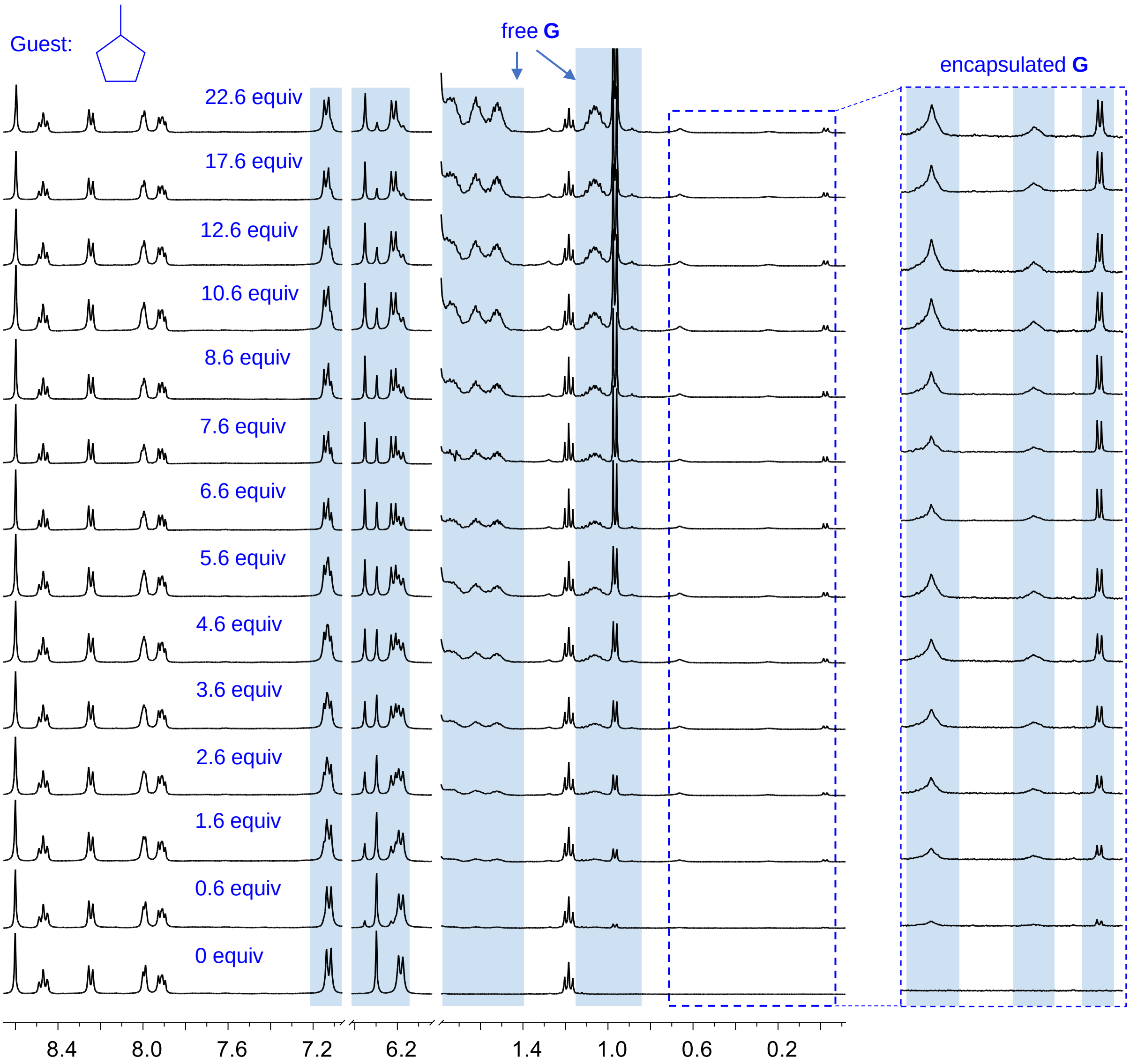

Figure S49. ^{1}H NMR (CD_3CN, 400 MHz, 298 K) titrations of the methylcyclopentane guest into a solution of **2** (3.0 mM).

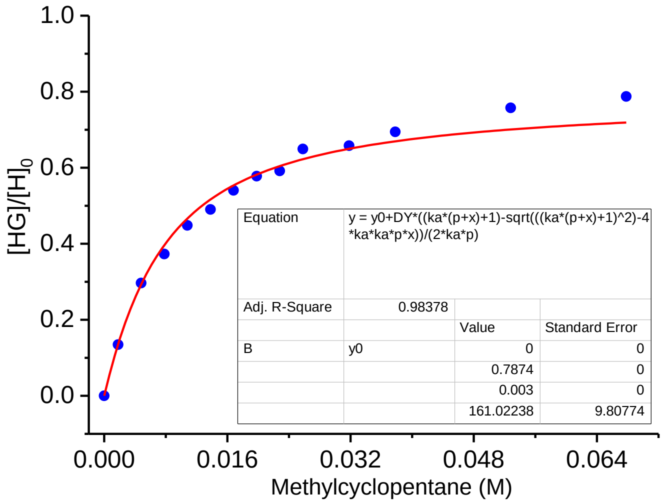

Figure S50. Degree of encapsulation, $[HG]/[H]_0$, as a function of the total concentration of methylcyclopentane present in solution and the corresponding fit of the data to the 1:1 binding model, giving a binding constant of (1.6 ± 0.1) × 10^{2} M^{-1} for methylcyclopentane⊂**2**.

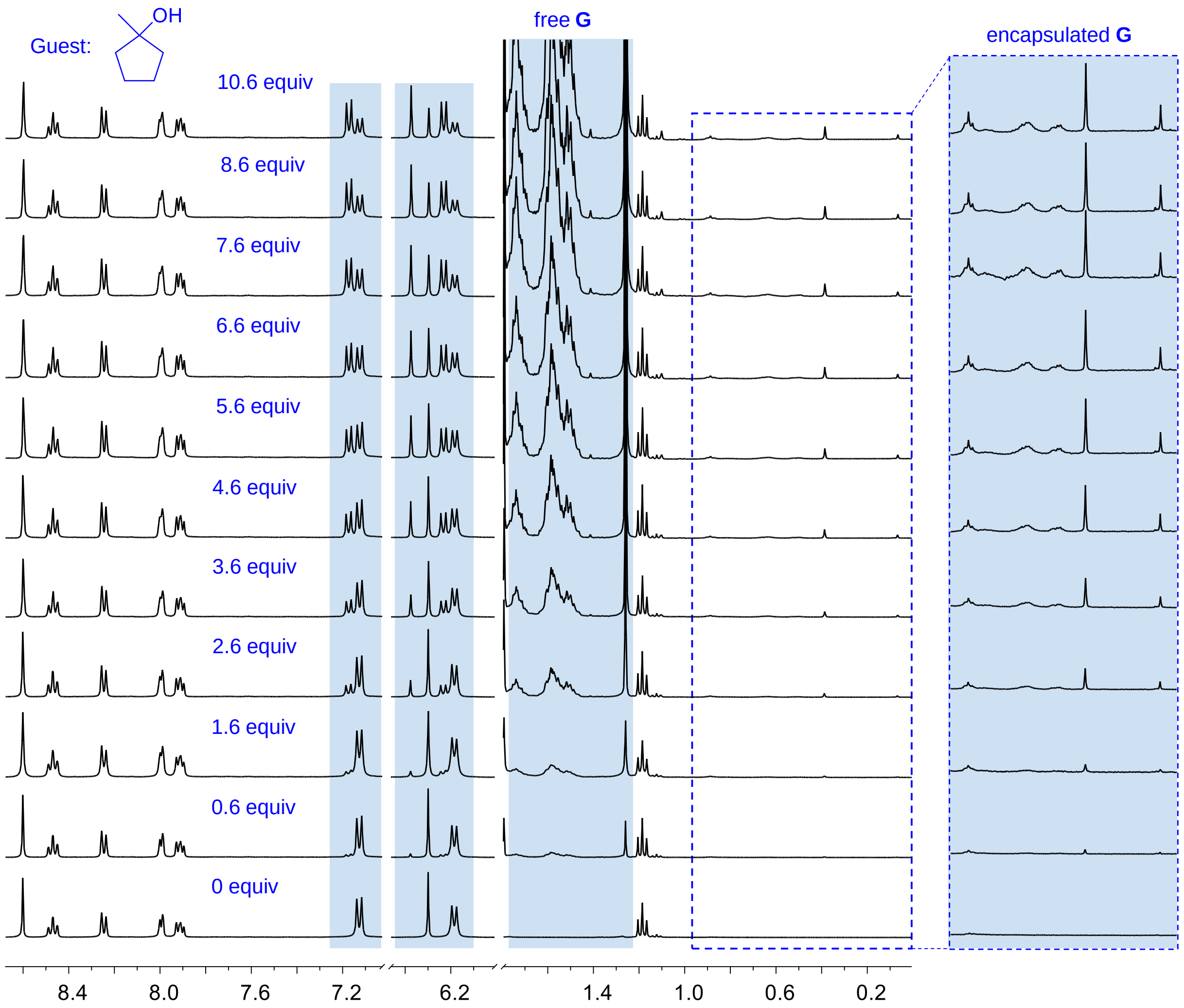

Figure S51. ^{1}H NMR (CD_3CN, 400 MHz, 298 K) titrations of the 1-methylcyclopentanol guest into a solution of **2** (3.0 mM).

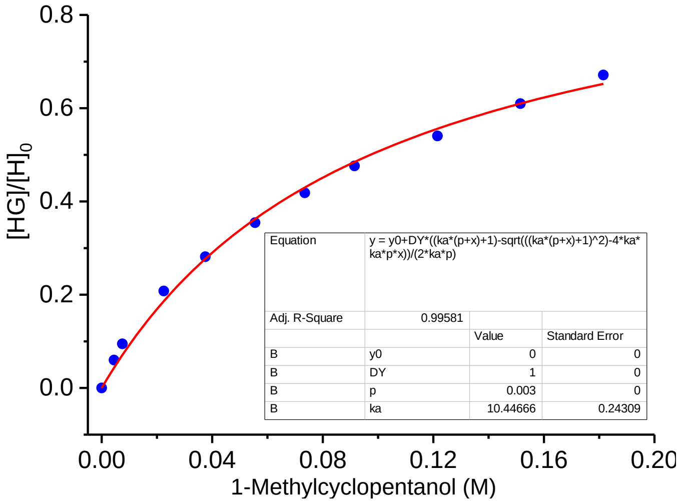

Figure S52. Degree of encapsulation, $[HG]/[H]_0$, as a function of the total concentration of 1-methylcyclopentanol present in solution and the corresponding fit of the data to the 1:1 binding model, giving a binding constant of 10.5 ± 0.2 M^{-1} for 1-methylcyclopentanol⊂**2**.

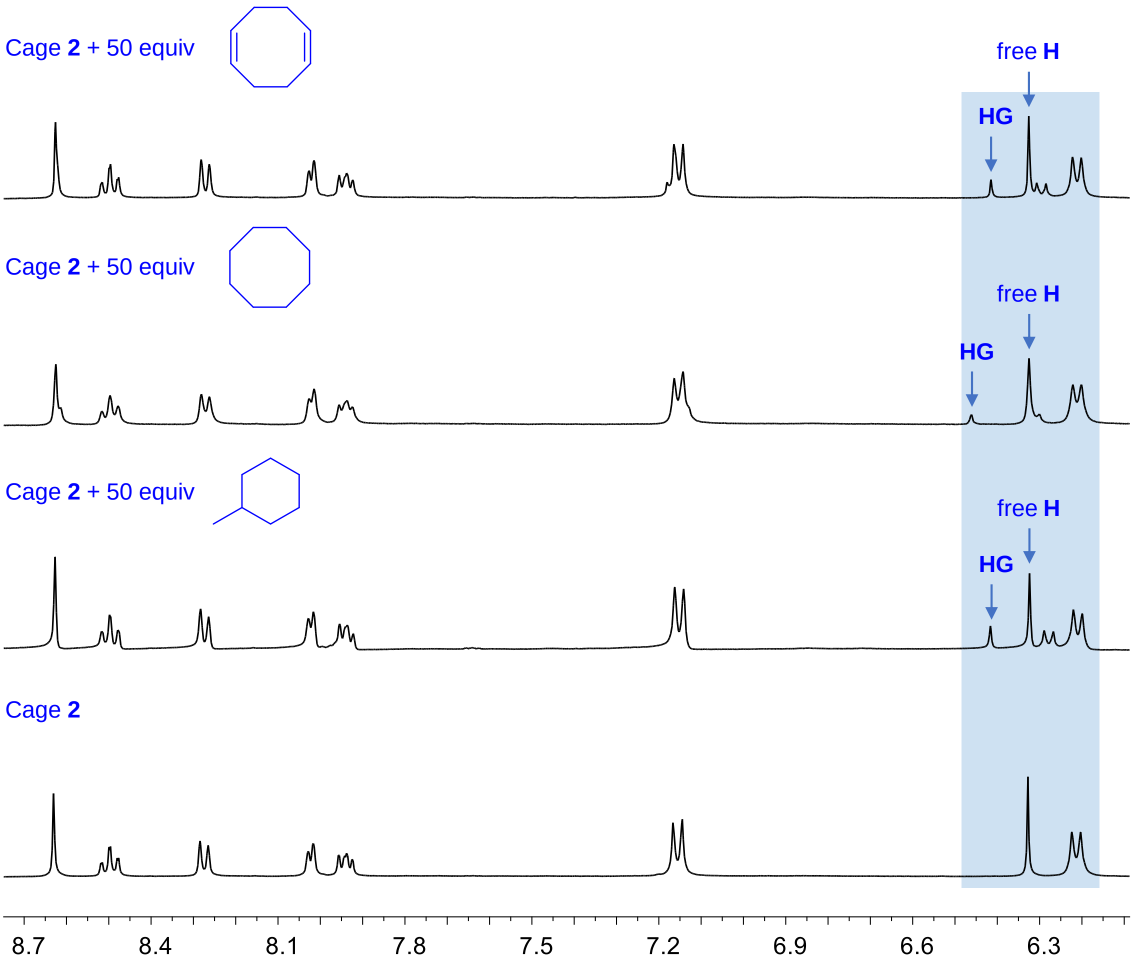

Figure S53. ^{1}H NMR (CD_3CN, 400 MHz, 298 K) spectra of **2** after addition of 50 equiv of methylcyclohexane, cyclooctane, and 1,5-cyclooctadiene, respectively. As the new peaks of the host-guest complexes after addition of 50 equiv of guests are very weak, the ^{1}H NMR titrations were not conducted to determine the binding constants.

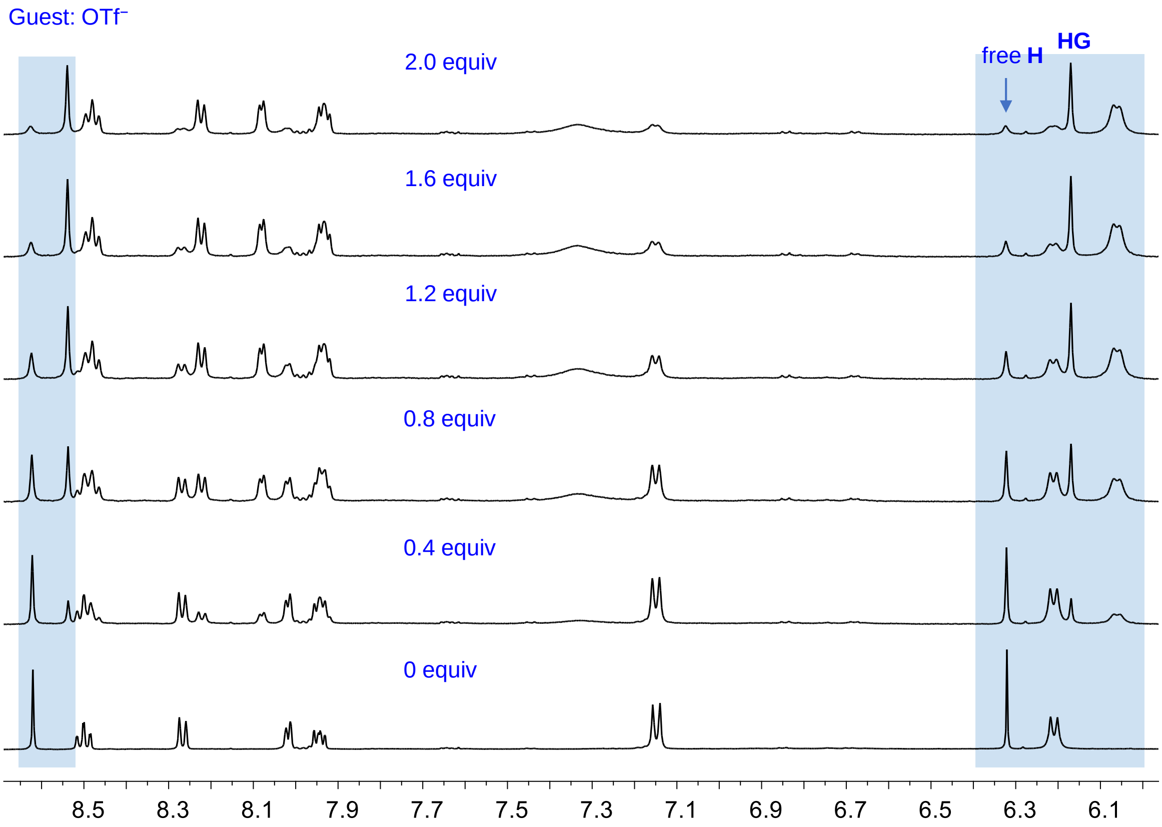

Figure S54. ^{1}H NMR (CD_3CN, 500 MHz, 298 K) titrations of OTf^- into a solution of **2** (0.10 mM). The binding constant was calculated to be (3.1 ± 0.1) × 10^4 M^{-1} for OTf^-⊂**2**.

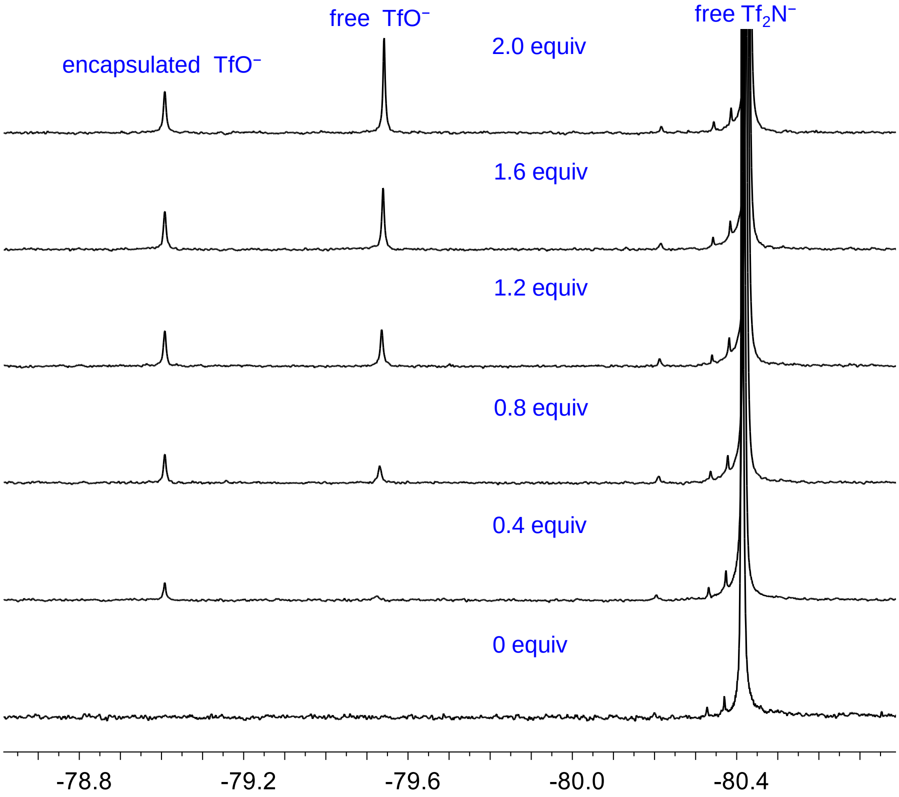

Figure S55. ^{19}F NMR (CD_3CN, 470.4 MHz, 298 K) titrations of TfO^- into a solution of **2** (0.10 mM).

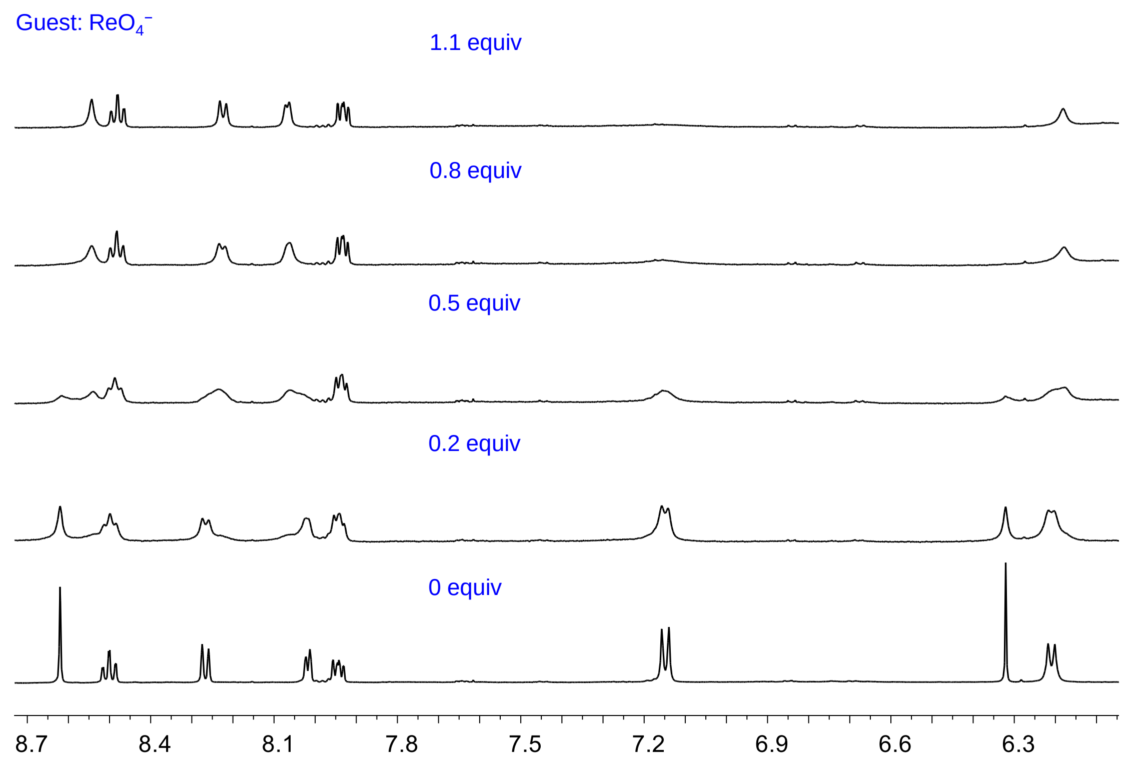

Figure S56. ^{1}H NMR (CD_3CN, 500 MHz, 298 K) titrations of ReO_4^- into a solution of **2** (0.10 mM). Due to the broadening of the peaks during the titrations, the binding constant of **2** for ReO_4^- was unable to calculate.

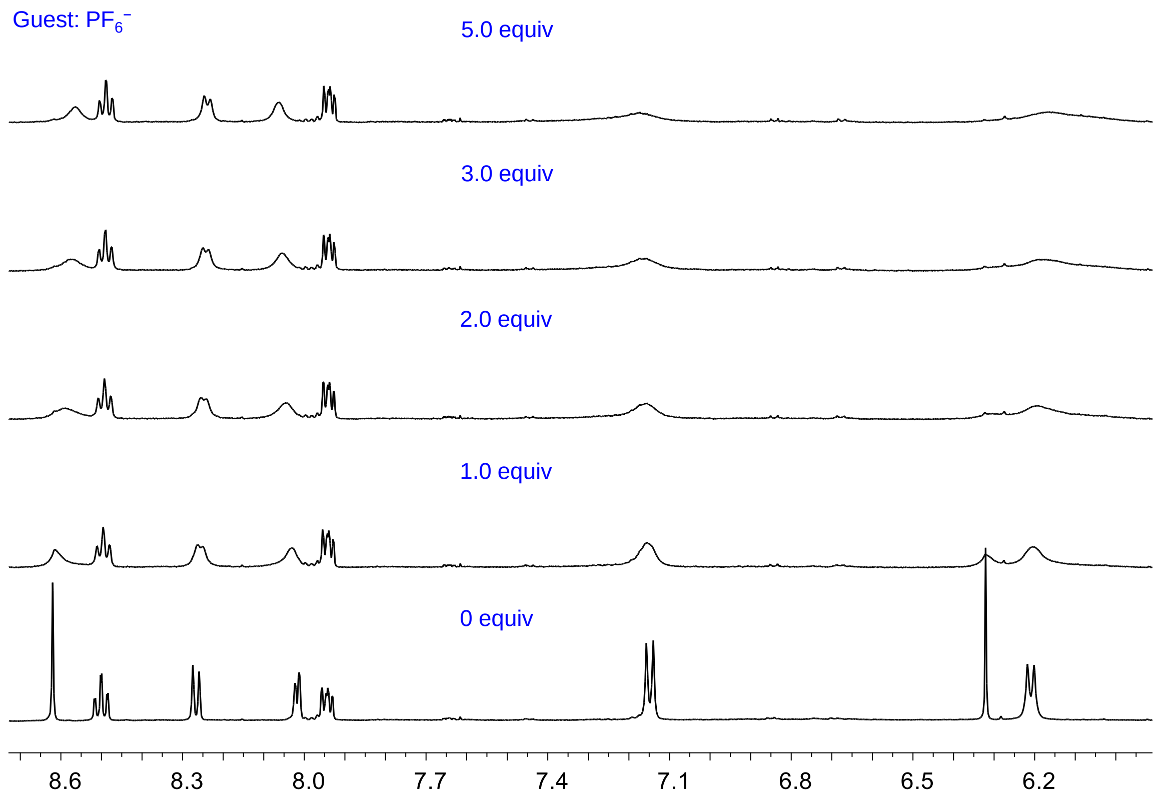

Figure S57. ^{1}H NMR (CD_3CN, 500 MHz, 298 K) titrations of PF_6^- into a solution of **2** (0.10 mM). Due to the broadening of the peaks during the titrations, the binding constant of **2** for PF_6^- was unable to calculate.

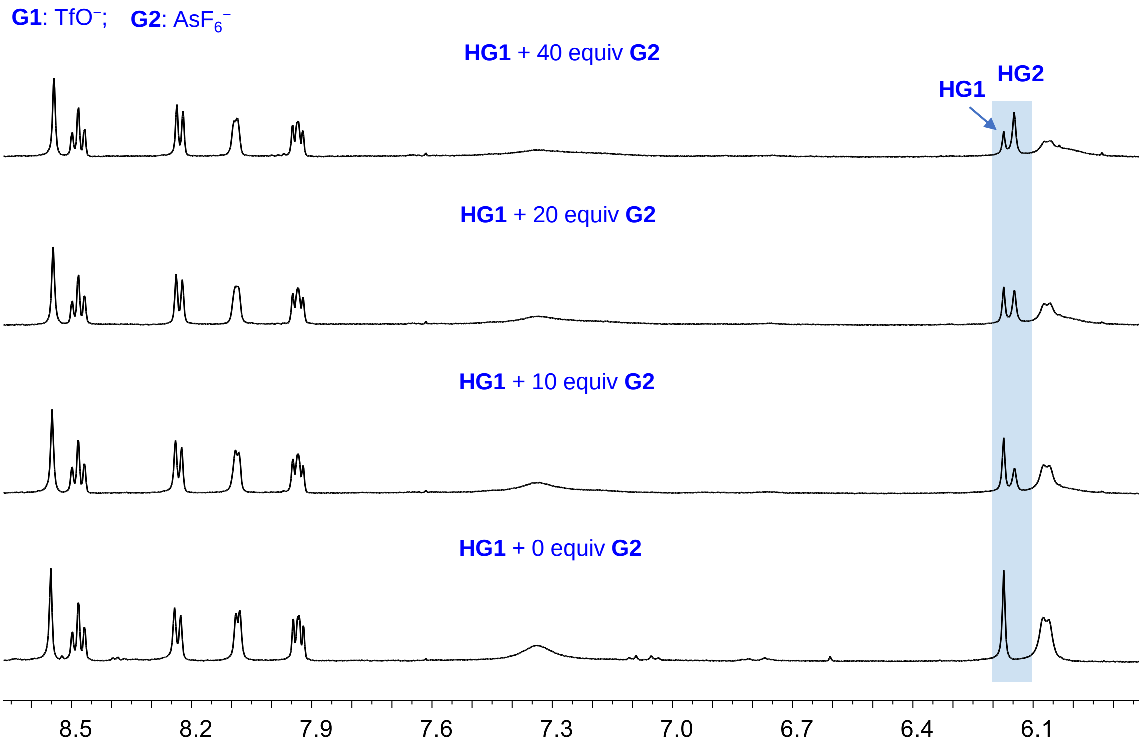

Figure S58. Competitive titrations of AsF_6^- into a solution of $TfO^-\subset$**2** (0.10 mM), which was prepared by adding 16 equiv TfO^- to **2** in CD_3CN, monitored by ^1H NMR spectroscopy (CD_3CN, 500 MHz, 298K). The relative binding constant for AsF_6^- versus TfO^- was estimated to be 0.97. The binding constant of **2** for AsF_6^- was calculated to be $(3.0 \pm 0.1) \times 10^4$ M^{-1}.

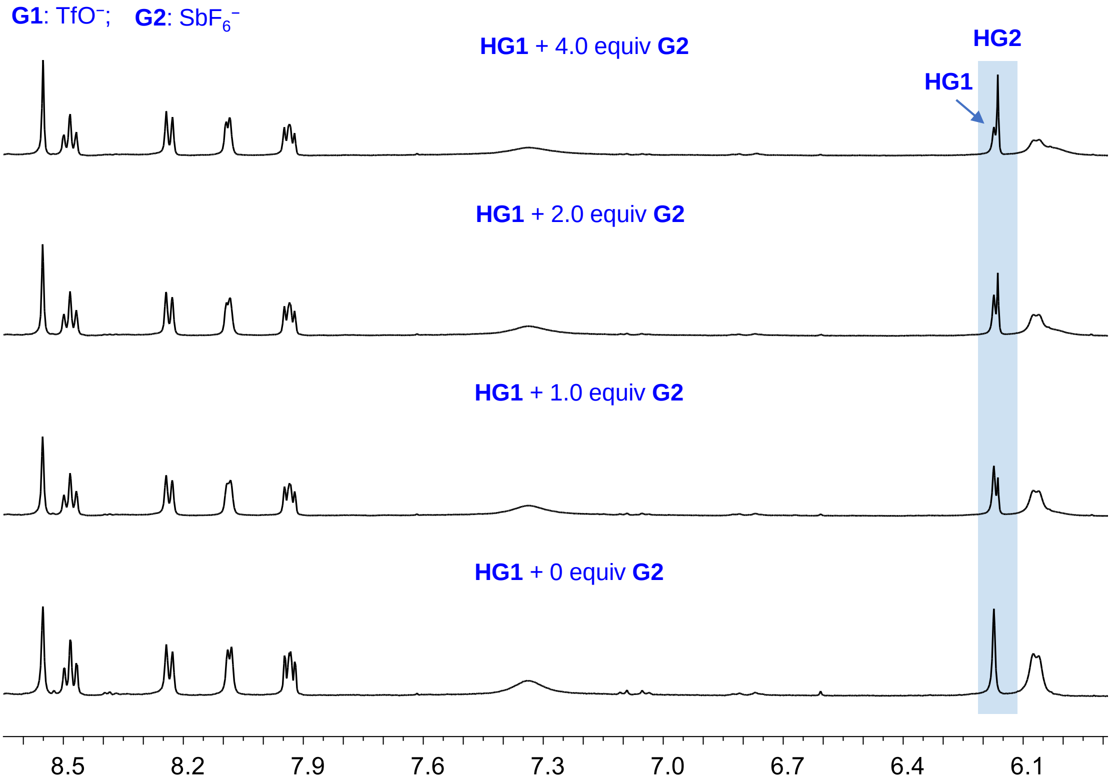

Figure S59. Competitive titrations of SbF_6^- into a solution of $TfO^-\subset$**2** (0.10 mM), which was prepared by adding 16 equiv TfO^- to **2** in CD_3CN, monitored by ^1H NMR spectroscopy (CD_3CN, 500 MHz, 298K). The relative binding constant for SbF_6^- versus TfO^- was estimated to be 5.8. The binding constant of **2** for SbF_6^- was calculated to be $(1.8 \pm 0.1) \times 10^5$ M^{-1}.

6. Acid resistance studies of cages

The studies of the acid resistance ability of cages were investigated in CD_3CN by progressive addition of different amounts of triflimide acid ($HNTf_2$). After each addition of acid to the cage solution, the sample was kept at room temperature for five minutes to reach equilibrium prior to recording the corresponding ^1H NMR spectrum. The amount of the remaining cage was determined by integration of the ^1H NMR spectra with 1,3,5-triethylbenzene as an internal standard. N,N-diisopropylethylamine was used as the base to test the recovery of the cage after its disassembly.

6.1 Acid resistance of cage 1

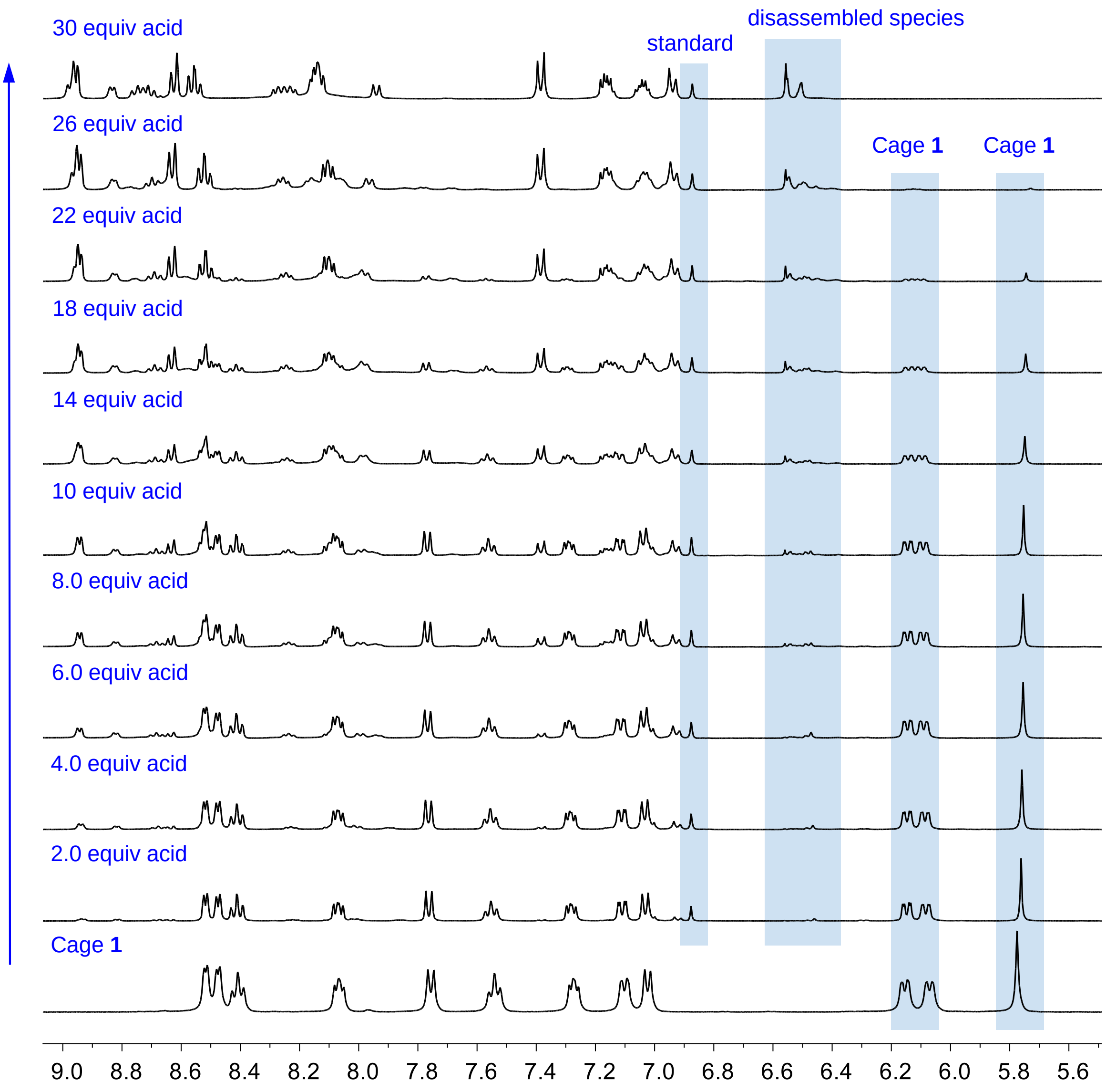

Figure S60. ^1H NMR spectra (CD_3CN, 400 MHz, 298 K) of **1** (3.3 mM) after addition of different equivalents of triflimide acid. Typical peaks from cage **1**, disassembled species, and the 1,3,5-triethylbenzene standard have been labelled.

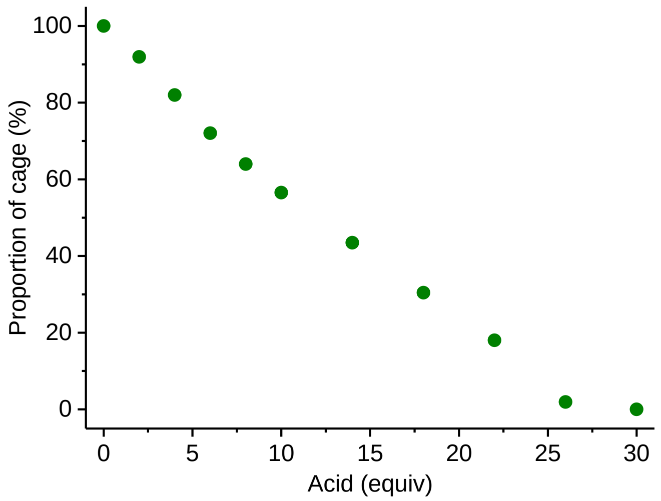

Figure S61. The proportion of cage **1** relative to its initial concentration as a function of the equivalents of the added acid ($HNTf_2$) in Figure S60.

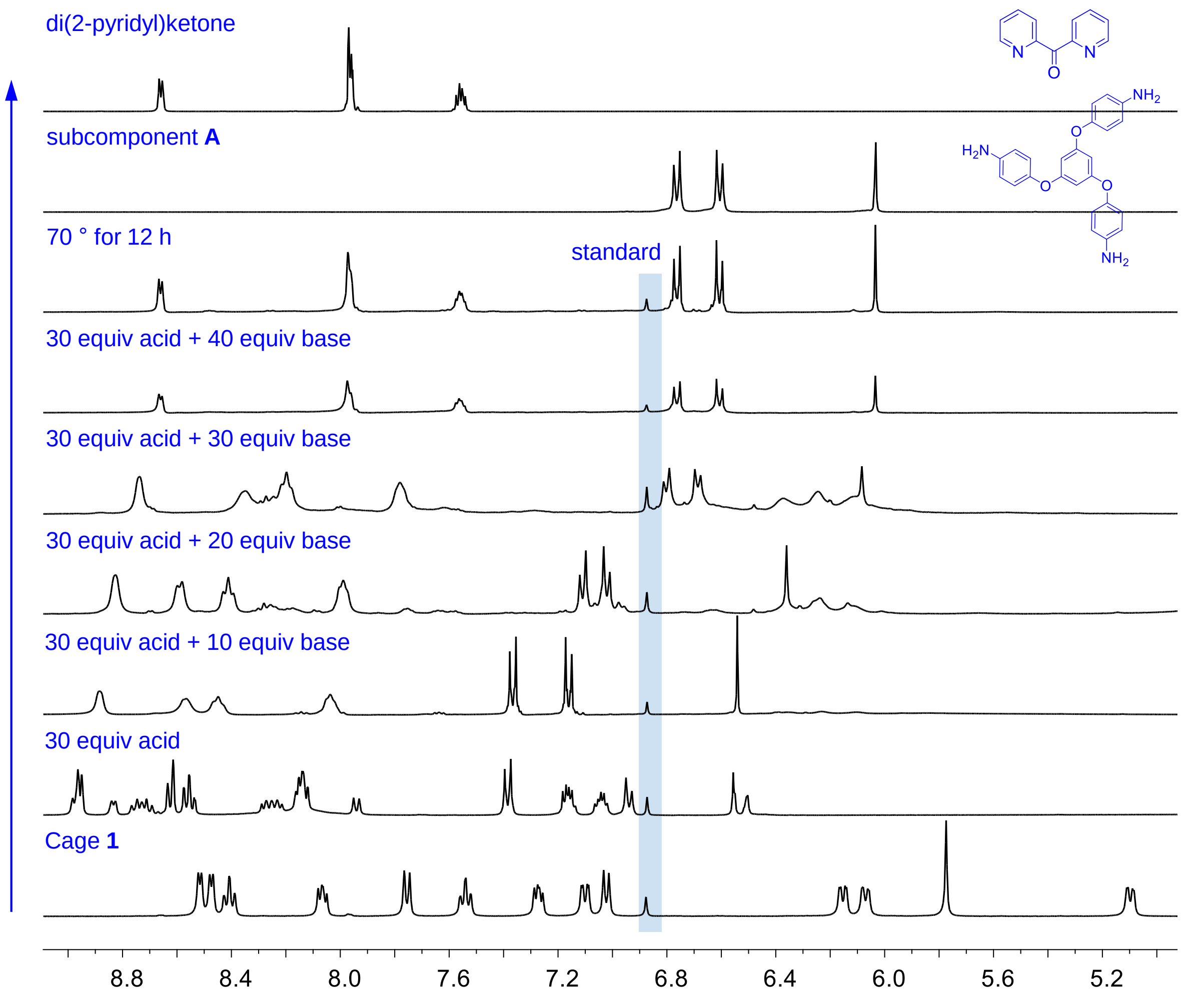

Figure S62. ^{1}H NMR spectra (CD_3CN, 400 MHz, 298 K) of cage **1** (3.3 mM) when in the presence of 30 equiv of acid ($HNTf_2$) and different equivalents of base (N,N-diisopropylethylamine), followed by heating at 70 °C for 12 hours, and the ^{1}H NMR spectra (CD_3CN, 400 MHz, 298 K) of the two subcomponents, 1,3,5-tris(4'-aminophenyloxy)benzene and di(2-pyridyl)ketone.

6.2 Acid resistance of cage 2

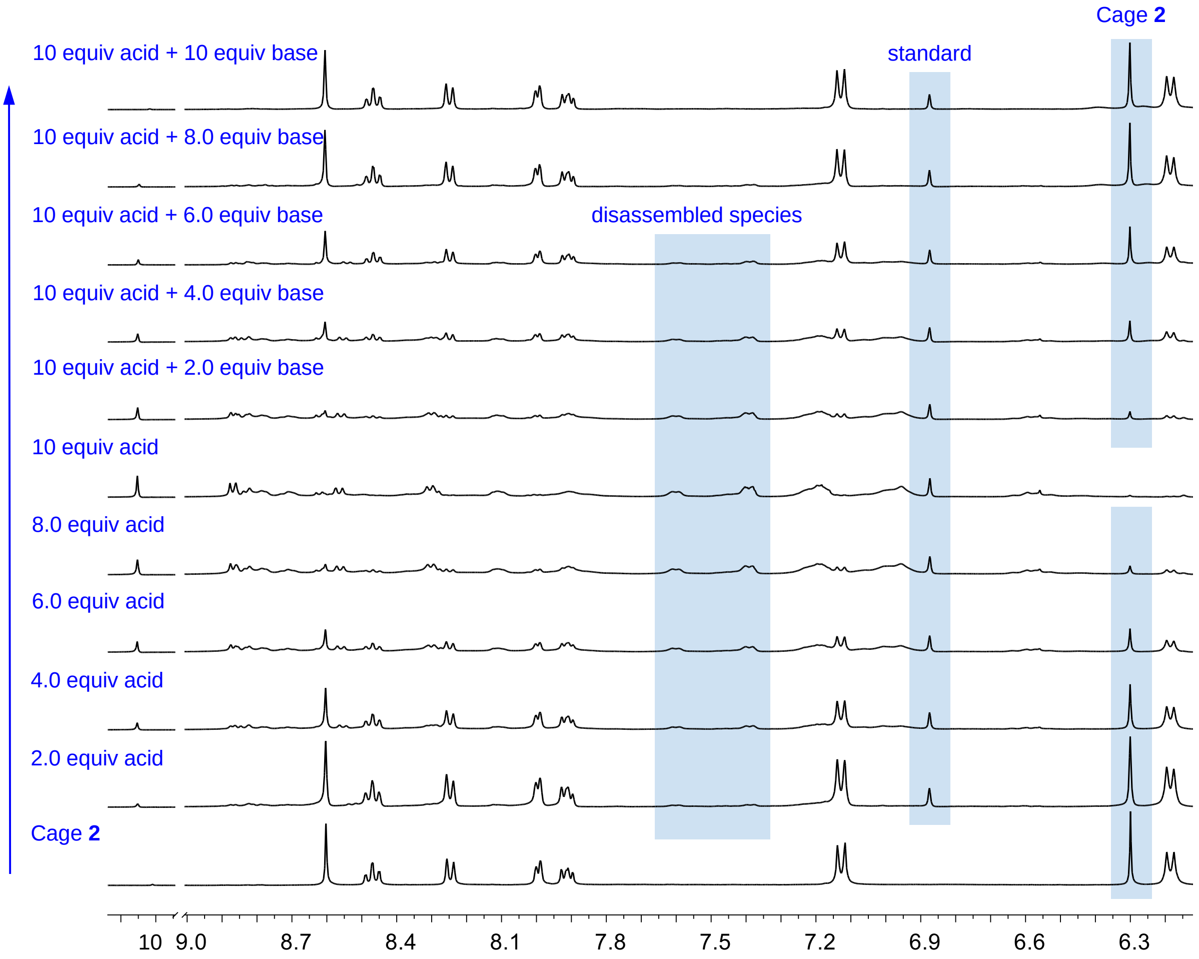

Figure S63. ^{1}H NMR spectra (CD_3CN, 400 MHz, 298 K) of **2** (3.3 mM) after addition of different equivalents of acid ($HNTf_2$) and base (N,N-diisopropylethylamine). Typical peaks from **2**, disassembled species, and the 1,3,5-triethylbenzene internal standard have been labelled.

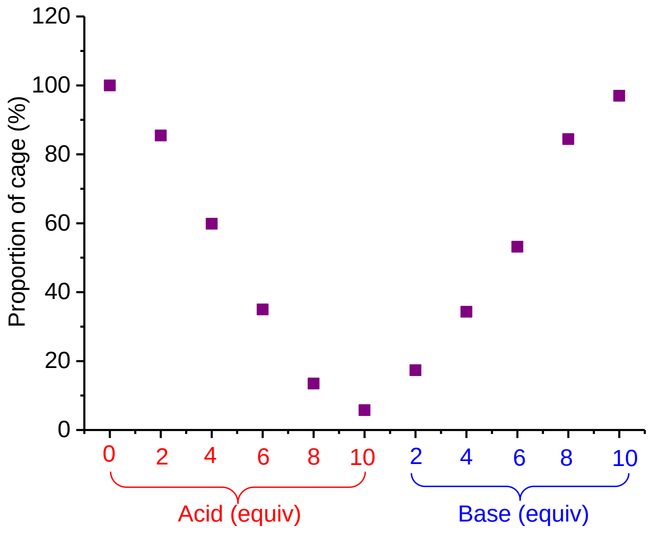

Figure S64. The proportion of cage **2** relative to its initial concentration as a function of the equivalents of the added acid ($HNTf_2$) and base (N,N-diisopropylethylamine) in Figure S63.

6.3 Selective cage disassembly and reassembly among a mixture of cages

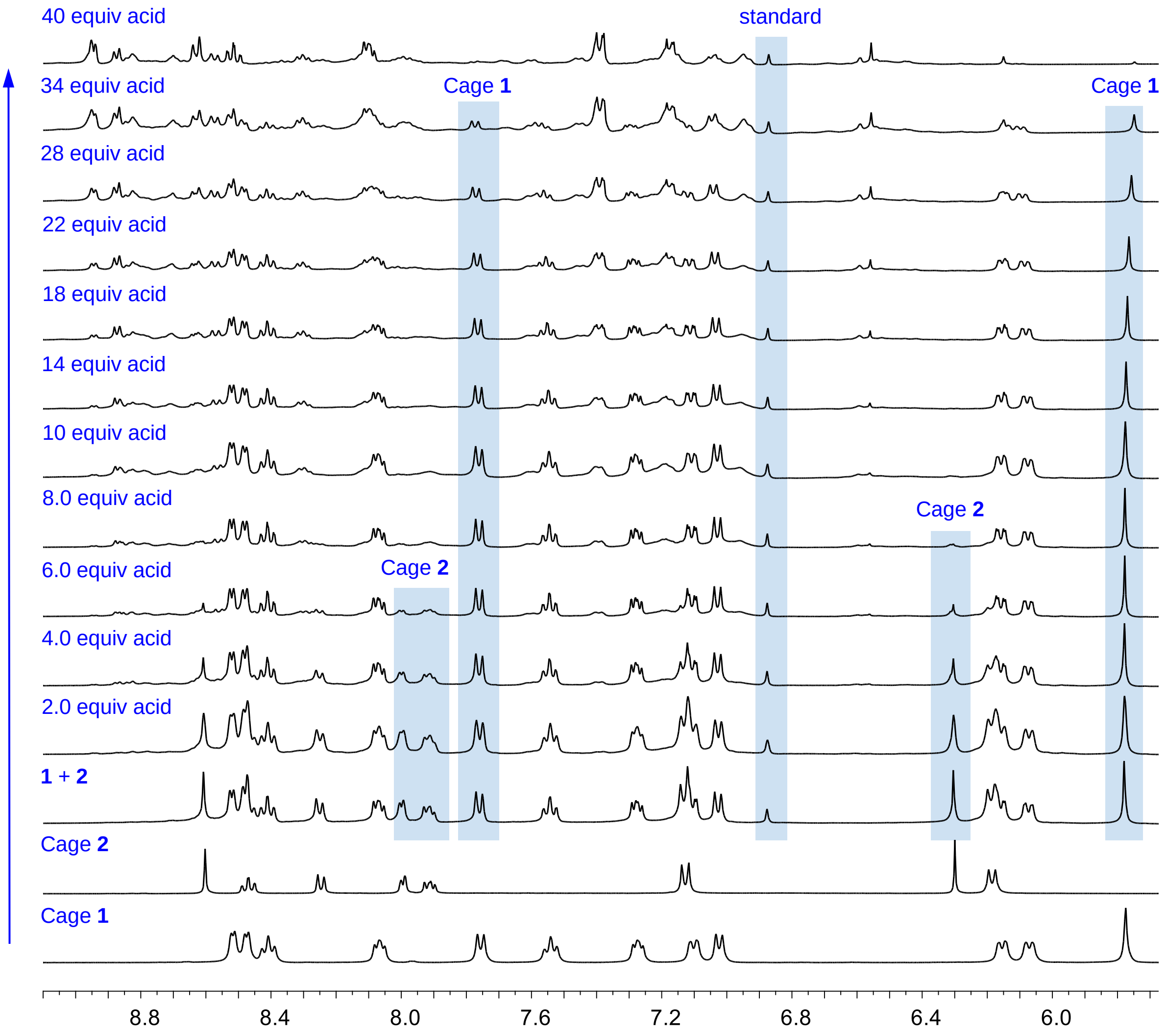

Figure S65. ^{1}H NMR spectra (CD_3CN, 400 MHz, 298 K) of an equimolar mixture of cages **1** and **2** (3.3 mM) after addition of different equivalents of triflimide acid. Typical peaks from **1**, **2**, and the 1,3,5-triethylbenzene internal standard have been labelled.

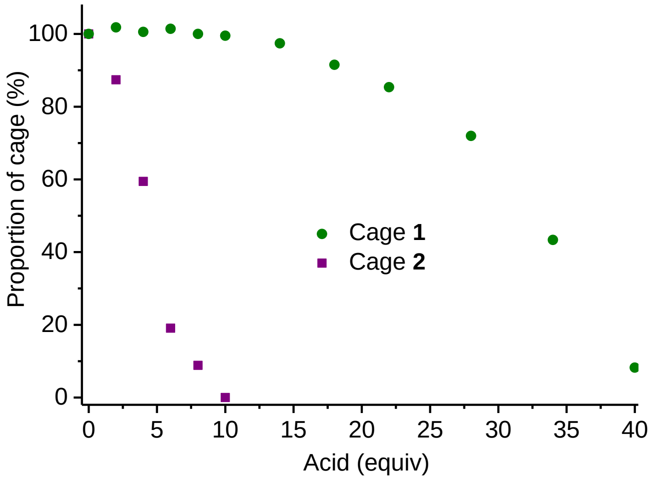

Figure S66. The proportions of cages **1** and **2** relative to their initial concentrations as a function of the equivalents of the added acid ($HNTf_2$) in Figure S65.

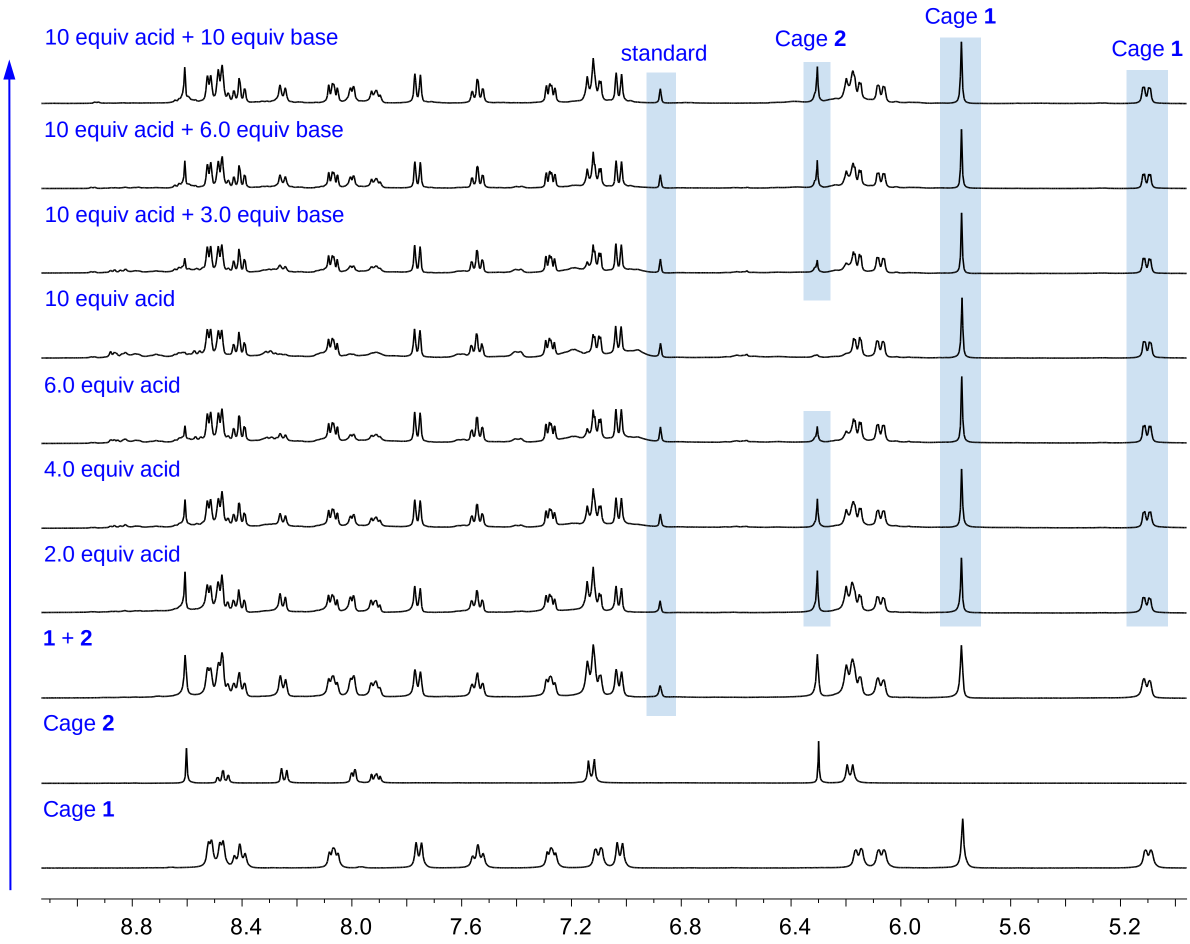

Figure S67. ^{1}H NMR spectra (CD_3CN, 400 MHz, 298 K) of an equimolar mixture of cages **1** and **2** (3.3 mM) after addition of different equivalents of acid ($HNTf_2$) and base (N,N-diisopropylethylamine). Typical peaks from **1**, **2**, and the 1,3,5-triethylbenzene internal standard have been labelled.

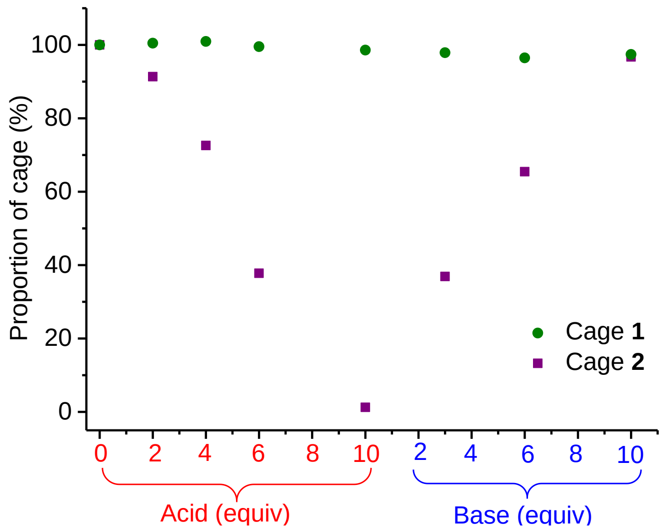

Figure S68. The proportions of cages **1** and **2** relative to their initial concentrations as a function of the equivalents of the added acid ($HNTf_2$) and base (N,N-diisopropylethylamine) in Figure S67.

7. Cargo delivery between cages 1 and 2

The experiments of SbF_6^- delivery from cage **2** to cage **1** using acid and base as chemical stimuli were carried out as following: To an NMR tube, a solution (500 μL) of SbF_6^-⊂**2** at 3 mM was prepared by adding SbF_6^- (1.0 equiv) to **2** (3 mM) in CD_3CN using 1,3,5-triethylbenzene as an internal standard. It was found that under these conditions, around 95% of the cage was occupied by the SbF_6^- guest with 5% of the cage being empty. Solid cage **1** (1.0 equiv) was then added into the solution to prepare an equimolar mixture of **1** and SbF_6^-⊂**2** (3 mM). Different equivalents of acid ($HNTf_2$, up to 9.0 equiv) and base (N,N-diisopropylethylamine, up to 9.0 equiv) were sequentially added and ^{1}H NMR spectra were recorded to monitor the SbF_6^- transfer from cage **2** to cage **1**. Control experiments without adding any acid or base into the solution were also performed to investigate the unassisted cargo delivery process from **2** to **1** by recording the ^{1}H NMR spectra of the mixture after different periods of time.

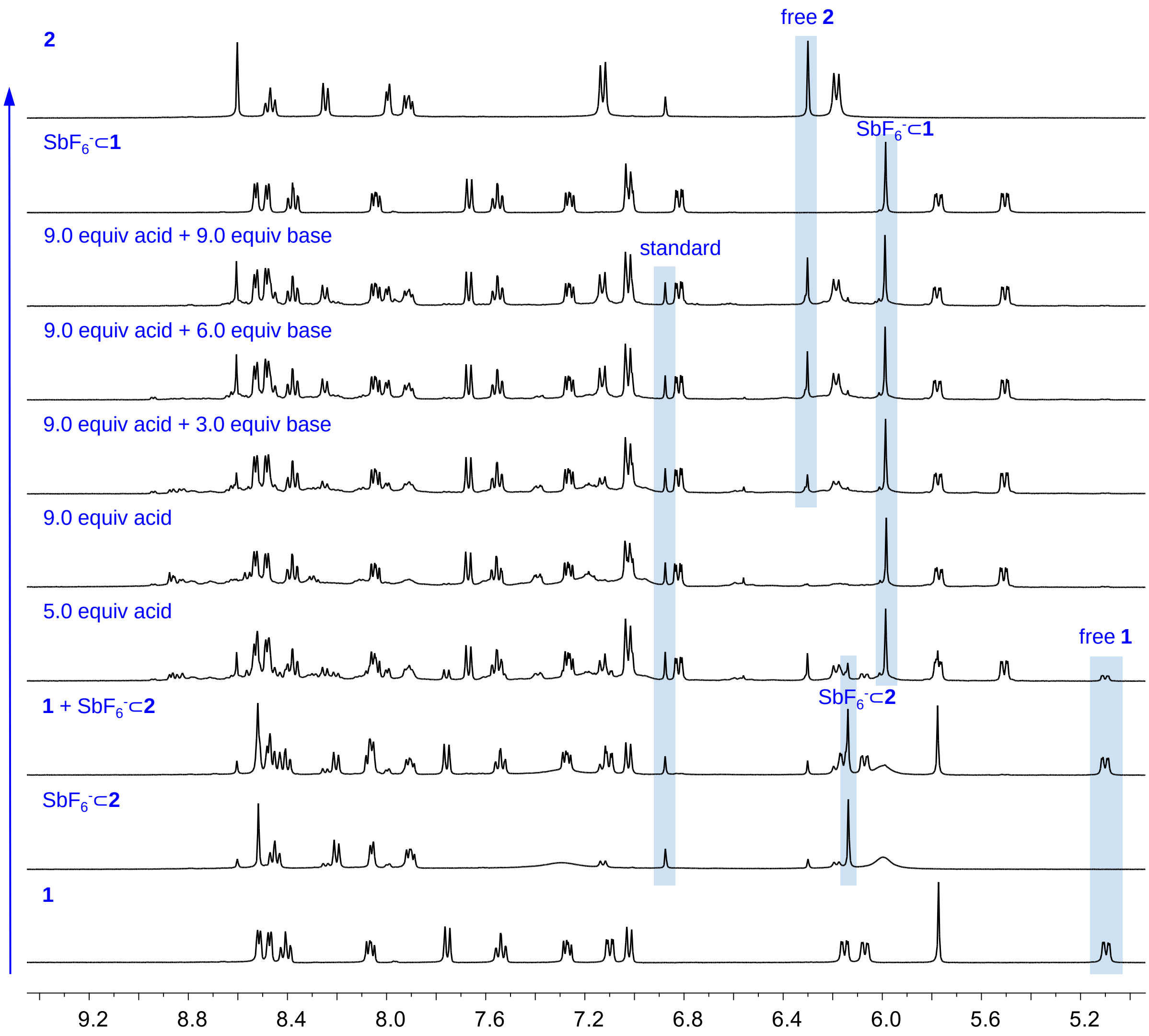

Figure S69. ^{1}H NMR spectra (CD_3CN, 400 MHz, 298 K) of an equimolar mixture of **1** and SbF_6^-⊂**2** (3.0 mM) when in the presence of different equivalents of acid ($HNTf_2$), followed by addition of different equivalents of base (N,N-diisopropylethylamine), and the ^{1}H NMR spectra (CD_3CN, 400 MHz, 298 K) of **1**, **2**, SbF_6^-⊂**1**, and SbF_6^-⊂**2** for comparison. Typical peaks from these species and the 1,3,5-tris(4'-aminophenyloxy)benzene internal standard have been labelled.

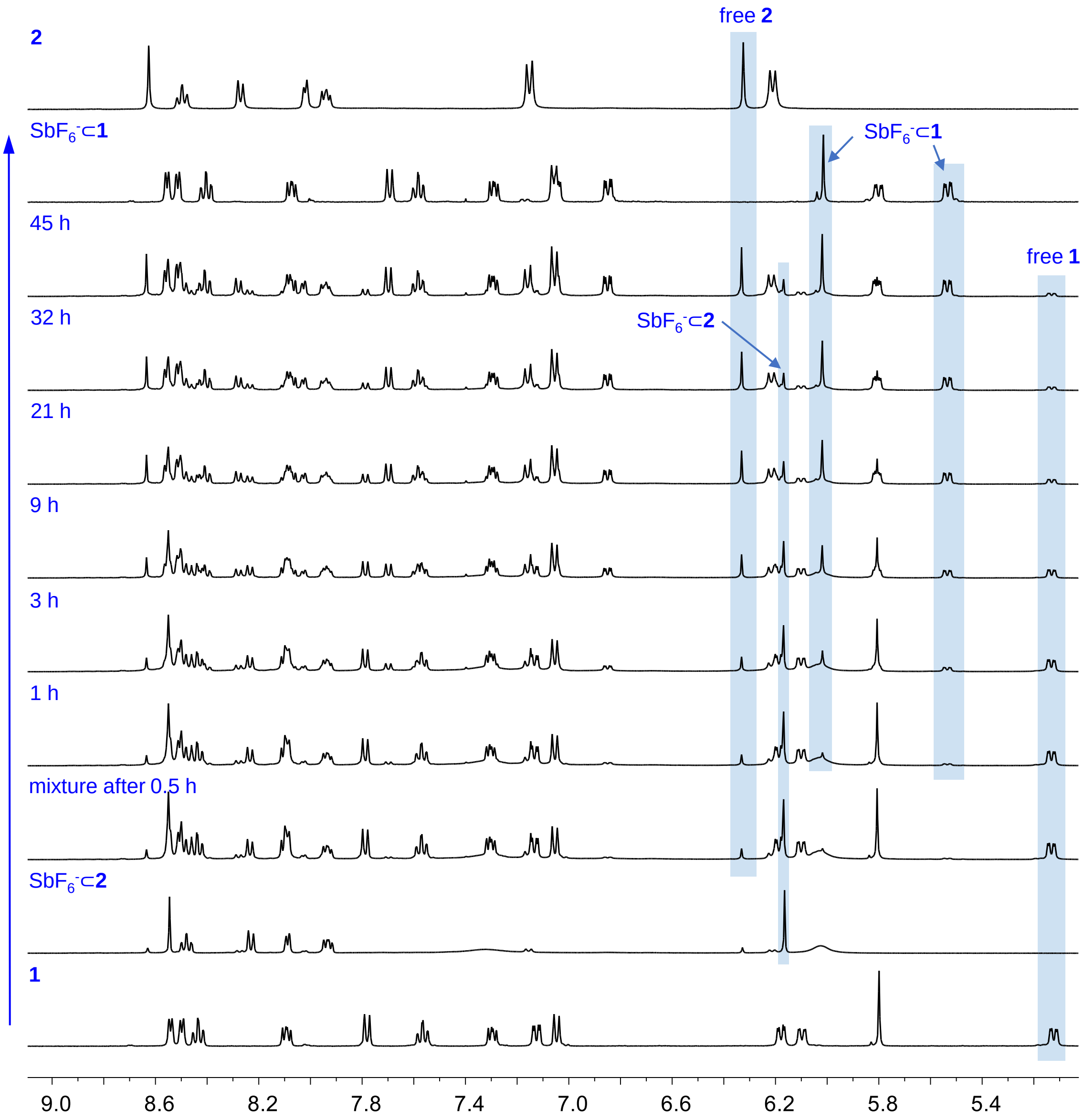

Figure S70. ^{1}H NMR spectra (CD_3CN, 400 MHz, 298 K) of an equimolar mixture of **1** and SbF_6^-⊂**2** (3.0 mM) after mixing for different periods of time, and the ^{1}H NMR spectra (CD_3CN, 400 MHz, 298 K) of **1**, **2**, SbF_6^-⊂**1**, and SbF_6^-⊂**2** for comparison. Typical peaks from these species have been labelled. The solution was found to take 45 hours at room temperature to reach a steady state after mixing **1** and SbF_6^-⊂**2**, which contained approximately 80% SbF_6^-⊂**1** and 20% SbF_6^-⊂**2**.

8. Cargo exchange between cages 1 and 2

The experiments of cargo exchange between SbF_6^-⊂**2** and 1,3-dioxane⊂**1** using acid and base as chemical stimuli were carried out as following: To an NMR tube, a solution (450 μL) of SbF_6^-⊂**2** at 3.3 mM was prepared by adding SbF_6^- (1.0 equiv) to **2** (3.3 mM) in CD_3CN using 1,3,5-triethylbenzene as an internal standard. It was found that under these conditions, around 95% of the cage was occupied by the SbF_6^- guest with 5% of the cage being empty. A solution (450 μL) of 1,3-dioxane⊂**1** (3.3 mM), which was prepared by mixing the 1,3-dioxane guest (40 equiv) and **1** (3.3 mM) in CD_3CN, was then added to the sample to prepare an equimolar mixture of 1,3-dioxane⊂**1** and SbF_6^-⊂**2** (1.7 mM). Different equivalents of acid ($HNTf_2$, up to 10 equiv) and base (N,N-diisopropylethylamine, up to 10 equiv) were sequentially added and ^1H NMR spectra were recorded to monitor the cargo exchange between the two cages. Control experiments without adding any acid or base into the solution were also performed to investigate the unassisted cargo exchange process between cages **1** and **2**, by recording the ^1H NMR spectra of the mixture after different periods of time.

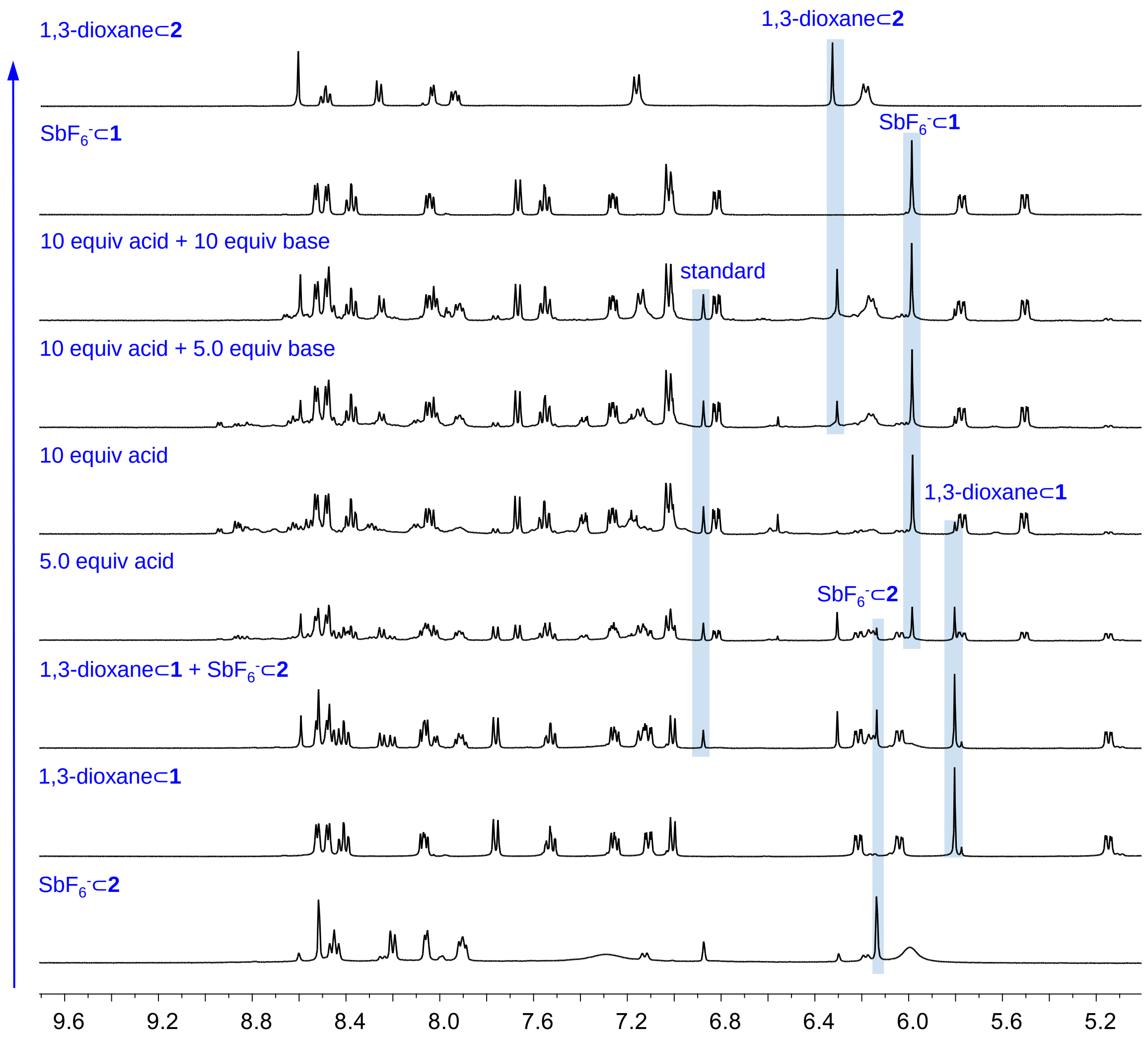

Figure S71. ^1H NMR spectra (CD_3CN, 400 MHz, 298 K) of an equimolar mixture of 1,3-dioxane⊂**1** and SbF_6^-⊂**2** (1.7 mM) when in the presence of different equivalents of acid ($HNTf_2$), followed by addition of different equivalents of base (N,N-diisopropylethylamine), and the ^1H NMR spectra (CD_3CN, 400 MHz, 298 K) of 1,3-dioxane⊂**1**, SbF_6^-⊂**1**,

1,3-dioxane⊂**2**, and SbF_6^-⊂**2** for comparison. Typical peaks from these species and the 1,3,5-tris(4'-aminophenyloxy)benzene internal standard have been labelled. It was found that after mixing 1,3-dioxane⊂**1** and SbF_6^-⊂**2** within 10 minutes, a small amount of complex 1,3-dioxane⊂**2** formed, indicated by the ^1H NMR spectrum of the mixture, due to the presence of the excess 1,3-dioxane guest (40 equiv) in the solution that displace the bound SbF_6^- within **2** weakly.

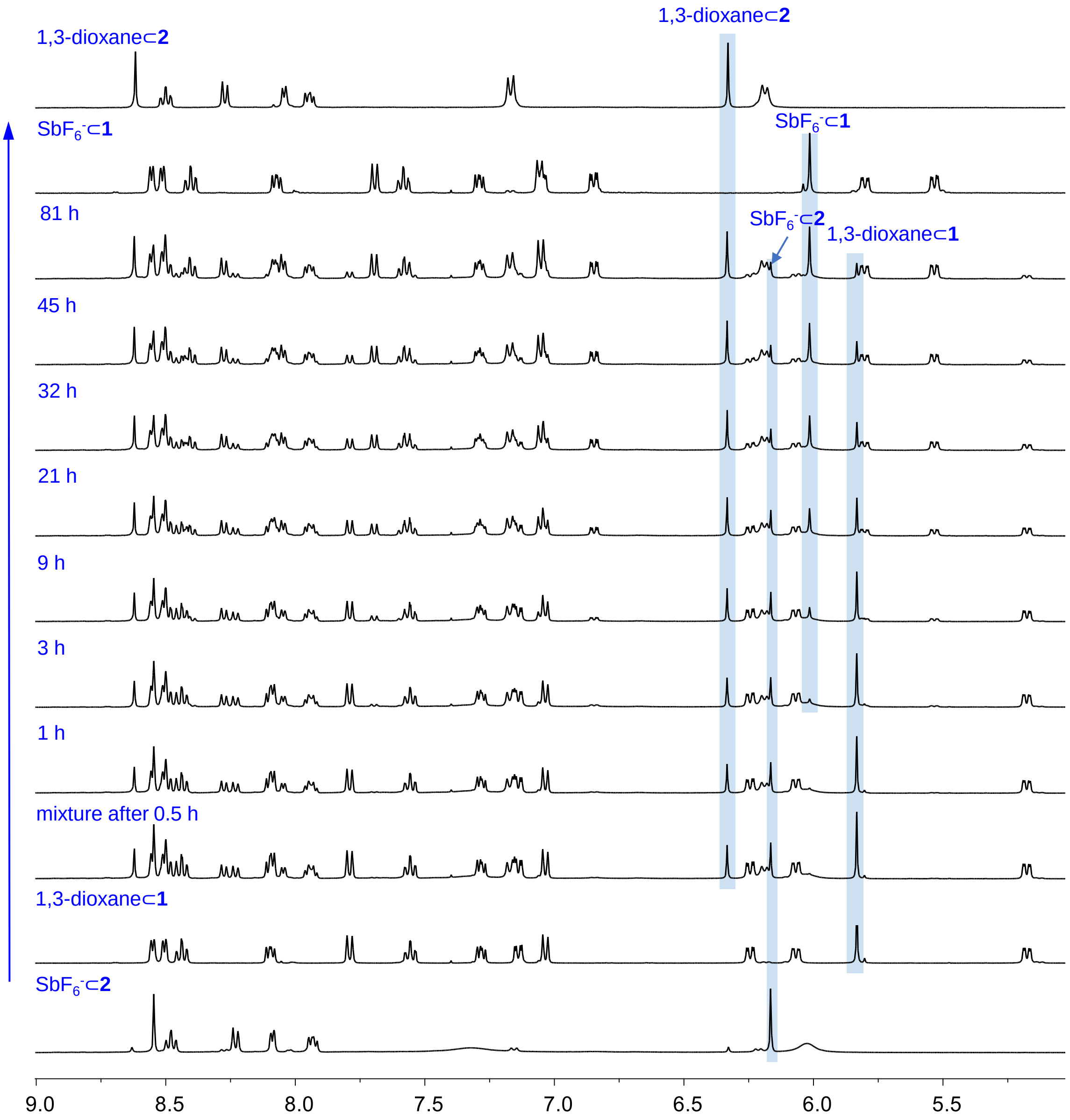

Figure S72. ^1H NMR spectra (CD_3CN, 400 MHz, 298 K) of an equimolar mixture of 1,3-dioxane⊂**1** and SbF_6^-⊂**2** (1.7 mM) after mixing for different periods of time, and the ^1H NMR spectra (CD_3CN, 400 MHz, 298 K) of 1,3-dioxane⊂**1**, SbF_6^-⊂**1**, 1,3-dioxane⊂**2**, and SbF_6^-⊂**2** for comparison. Typical peaks from these species have been labelled. The solution was found to take 81 hours at room temperature to reach a steady state after mixing 1,3-dioxane⊂**1** and SbF_6^-⊂**2**, which contained approximately 82% SbF_6^-⊂**1** and 18% SbF_6^-⊂**2** in terms of the SbF_6^- guest.

9. References

[1] M. Yamanaka, H. Fujii, *J. Org. Chem.* **2009**, *74*, 5390-5394.

[2] D. Allan, H. Nowell, S. Barnett, M. Warren, A. Wilcox, J. Christensen, L. Saunders, A. Peach, M. Hooper, L. Zaja, S. Patel, L. Cahill, R. Marshall, S. Trimnell, A. Foster, T. Bates, S. Lay, M. Williams, P. Hathaway, G. Winter, M. Gerstel, R. Wooley, *Crystals* **2017**, *7*, 336.

[3] a) Collaborative Computational Project, Number 4. *Acta Cryst.* **1994**, *D50*, 760; b) P. Evans, *Acta Cryst.* **2006**, *D62*, 72-82; c) G. Winter, *J. Appl. Cryst.* **2009**, *43*, 186-190.

[4] CrysalisPro; Agilent Technologies Yarton, Oxfordshire, UK, **2009-2016**.

[5] L. J. Farrugia, *J. Appl. Cryst.* **2012**, *45*, 849-854.

[6] P. R. Evans, G. N. Murshudov, *Acta Cryst.* **2013**, *D69*, 1204-1214.

[7] M. D. Winn, C. C. Ballard, K. D. Cowtan, E. J. Dodson, P. Emsley, P. R. Evans, R. M. Keegan, E. B. Krissinel, A. G. Leslie, A. McCoy, S. J. McNicholas, G. N. Murshudov, N. S. Pannu, E. A. Potterton, H. R. Powell, R. J. Read, A. Vagin, K. S. Wilson, *Acta Cryst.* **2011**, *D67*, 235-242.

[8] G. M. Sheldrick, *Acta Cryst.* **2015**, *A71*, 3-8.

[9] G. M. Sheldrick, *Acta Cryst.* **2015**, *C71*, 3-8.

[10] P. van der Sluis, A. L. Spek, *Acta Cryst.* **1990**, *A46*, 194-201.

[11] Spek, A. L. *PLATON: A Multipurpose Crystallographic Tool*; Utrecht University: Utrecht, The Netherlands, **2008**.

[12] Bricogne, G.; Blanc, E.; Brandle, M.; Flensburg, C.; Keller, P.; Paciorek, W.; Roversi, P.; Sharff, A.; Smart, O. S.; Vonrhein, C.; Womack, T. O. BUSTER; 2.11.2 ed.; Global Phasing Ltd.: Cambridge, United Kingdom, **2011**.

[13] Smart, O. S.; Womack, T. O. *Grade Web Server*; Global Phasing Ltd., **2014**.

[14] G. J. Kleywegt, T. A. Jones, *Acta Cryst.* **1994**, *D50*, 178-185.

[15] Y. R. Hristova, M. M. J. Smulders, J. K. Clegg, B. Breiner, J. R. Nitschke, *Chem. Sci.* **2011**, *2*, 638-641.

[16] A. M. Castilla, T. K. Ronson, J. R. Nitschke, *J. Am. Chem. Soc.* **2016**, *138*, 2342-2351.
